# Synthesis, Characterization, Antimicrobial Activity and Molecular Modeling Studies of Novel Indazole-Benzimidazole Hybrids

**DOI:** 10.3390/antibiotics14111150

**Published:** 2025-11-13

**Authors:** Redouane Er-raqioui, Sara Roudani, Imane El Houssni, Njabulo J. Gumede, Yusuf Sert, Ricardo F. Mendes, Dimitry Chernyshov, Filipe A. A. Paz, José A. S. Cavaleiro, Maria do Amparo F. Faustino, Rakib El Mostapha, Said Abouricha, Khalid Karrouchi, Maria da Graça P. M. S. Neves, Nuno M. M. Moura

**Affiliations:** 1 Laboratory of Molecular Chemistry Materials and Catalysis, Faculty of Sciences and Technics, Sultan Moulay Slimane University, B.P. 523, Beni-Mellal 23000, Morocco; redouane.er-raqoui@usms.ma (R.E.-r.); roudani.sara@usms.ma (S.R.); abouricha4@yahoo.fr (S.A.); 2 LAQV-REQUIMTE, Department of Chemistry, University of Aveiro, Campus Universitário de Santiago, 3810-193 Aveiro, Portugal; jcavaleiro@ua.pt (J.A.S.C.); faustino@ua.pt (M.d.A.F.F.); gneves@ua.pt (M.d.G.P.M.S.N.); 3 Higher School of Technology, Sultan Moulay Slimane University, B.P. 336, 23200, Fkih Ben Salah 23000, Morocco; 4 BVPRT, BER Center, Faculty of Sciences, Mohammed V University, Rabat 08007, Morocco; imane_elhoussni@um5.ac.ma; 5 Department of Chemical and Physical Sciences, Faculty of Natural Sciences, Walter Sisulu University (WSU), Private Bag X01, Mthatha 4099, Eastern Cape, South Africa; njgumede@wsu.ac.za; 6 Department of Physics, Yozgat Bozok University, 66100 Yozgat, Turkey; yusuf.sert@bozok.edu.tr; 7 CICECO—Aveiro Institute of Materials, Department of Chemistry, University of Aveiro, Campus Universitário de Santiago, 3810-193 Aveiro, Portugal; rfmendes@ua.pt (R.F.M.); filipe.paz@ua.pt (F.A.A.P.); 8 European Synchrotron Radiation Facility, SNBL CS40220, 38043 Grenoble CEDEX 9, France; dmitry.chernyshov@esrf.fr; 9 Laboratory of Analytical Chemistry and Bromatology, Faculty of Medicine and Pharmacy, Mohammed V University, Rabat 08007, Morocco; khalid.karrouchi@um5s.net.ma

**Keywords:** indazole, benzimidazole, hybrids, antimicrobial activity, molecular docking, in silico analysis

## Abstract

**Background/Objectives**: In this work, a series of six new indazole-benzimidazole hybrids (**M1**–**M6**) were designed, synthesized, and fully characterized. The design of these compounds was based on the combination of two pharmacophoric units, indazole and benzimidazole, both known for their broad spectrum of biological activities. **Methods**: The molecular hybridization strategy was planned to combine these scaffolds through an effective synthetic pathway, using 6-nitroindazole, two 2-mercaptobenzimidazoles, and 1,3- or 1,5-dihaloalkanes as key precursors, affording the desired hybrids in good yields and with enhanced biological activity. Quantum chemical calculations were performed to investigate the structural, electronic, and electrostatic properties of **M1**–**M6** molecules using Density Functional Theory (DFT) at the B3LYP/6-311++G(d,p) level. The antimicrobial activity efficacy of these compounds was assessed in vitro against four Gram-positive bacteria (*Staphylococcus aureus*, *Enterococcus faecalis*, *Bacillus cereus*, and *Lactobacillus plantarum*), four Gram-negative bacteria (*Salmonella enteritidis*, *Escherichia coli*, *Campylobacter coli*, *Campylobacter jejuni*), and four fungal strains (*Saccharomyces cerevisiae*, *Candida albicans*, *Candida tropicalis*, and *Candida glabrata*) using ampicillin and tetracycline as reference standard drugs. **Results**: Among the series, compound **M6** exhibited remarkable antimicrobial activity, with minimum inhibitory concentrations (MIC) of 1.95 µg/mL against *S. cerevisiae* and *C. tropicalis*, and 3.90 µg/mL against *S. aureus*, *B. cereus*, and *S. enteritidis*, while the standards Ampicillin (AmB) (MIC ≥ 15.62 µg/mL) and Tetracycline (TET) (MIC ≥ 7.81 µg/mL) exhibited higher MIC values. To gain molecular insights into the compounds, an in silico docking study was performed to determine the interactions of **M1**–**M6** ligands against the antimicrobial target beta-ketoacyl-acyl carrier protein (ACP) synthase III complexed with malonyl-COA (PDB ID: 1HNJ). Molecular modeling data provided valuable information on the structure-activity relationship (SAR) and the binding modes influencing the candidate ligand-protein recognition. Amino acid residues, such as Arg249, located in the solvent-exposed region, were essential for hydrogen bonding with the nitro group of the 6-nitroindazole moiety. Furthermore, polar side chains such as Asn274, Asn247, and His244 participated in interactions mediated by hydrogen bonding with the 5-nitrobenzimidazole moiety of these compound series. **Conclusions**: The hybridization of indazole and benzimidazole scaffolds produced compounds with promising antimicrobial activity, particularly **M6**, which demonstrated superior potency compared to standard antibiotics. Computational and docking analyses provided insights into the structure–activity relationships, highlighting these hybrids as potential candidates for antimicrobial drug development.

## 1. Introduction

Heterocyclic chemistry has attracted considerable interest from researchers due to its crucial role in both natural products and synthetic chemistry research during long periods. It is recognized for its significant impact on pharmaceuticals, agrochemicals, and advanced materials development [1,2,3]. Among the different heterocycles, natural derivatives bearing indazole, benzimidazole, and benzimidazole-indazole cores, have attracted particular attention for their wide-ranging biological activities, prompting ongoing efforts to design novel hybrid structures [4,5,6,7,8].

**Figure 1:**
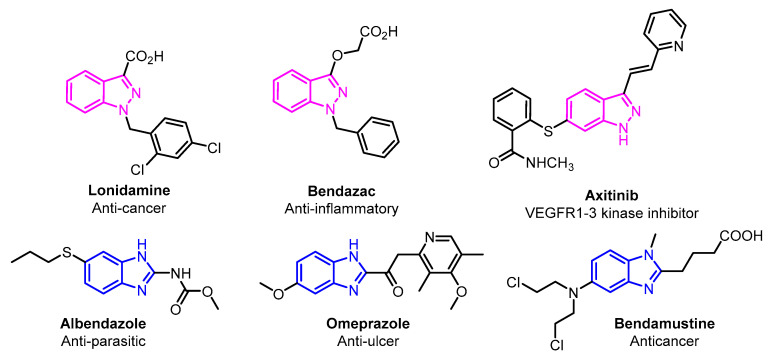
Chemical structures of selected biologically active molecules with indazole and benzimidazole nuclei.

**Figure 2:**
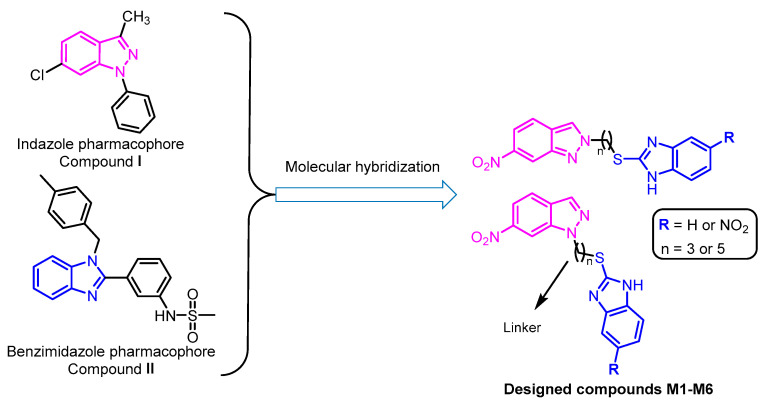
Rational design of the new indazole-benzimidazole hybrids **M1**–**M6**.

The indazole nucleus, characterized by a fused bicyclic system comprising a pyrazole and a benzene ring, forms the basis of a wide array of compounds with diverse pharmacological potentials [7,9]. Reported activities include anticancer [10,11], antiparasitic [12], antimicrobial [13,14], anti-tubercular [15], antidiabetic [16], antiprotozoal [17], anti-inflammatory [18], protein kinase inhibition [19,20] as well as inhibition of monoamine oxidase and nitric oxide synthase [21,22]. Many pharmaceutical drugs on the market are built on indazole scaffolds. For example, lonidamine is used as an anticancer in the treatment of brain tumors, bendazac as a non-steroidal anti-inflammatory agent, and axitinib, a second-generation tyrosine kinase inhibitor, that works by selectively inhibiting vascular endothelial growth factor receptors (VEGFR-1, VEGFR-2, VEGFR-3) (Figure 1). These examples demonstrate the value of this heterocyclic nucleus in various therapeutic areas. In 2024, Rayawgol and coworkers described compound **I** (Figure 2), a 1,3,6-trisubstituted indazole scaffold that possesses potent antifungal activity against *Candida albicans* and *Aspergillus niger* compared with Fluconazole and Amphotericin B [23].

Benzimidazoles represent another class of crucial bioactive and industrially relevant heterocycles. These compounds are recognized for their varied therapeutic properties, including antimicrobial [24], anti-inflammatory [25], anticancer [26], antioxidant [27], antiviral [28], anticonvulsant [29], and antihypertensive [30] properties. Also, several key pharmacologically bioactive drugs have a benzimidazole component in their structure, including albendazole, omeprazole, and bendamustine (Figure 1). Later, Dokla and coworkers [31] reported that Compound **II** (Figure 2) with a 1,2-disubstituted benzimidazole moiety bearing a sulfonamide group, possesses potent antibacterial activity against *E. coli.* (minimum inhibitory concentrations (MIC) value of 2 µg/mL compared to Gentamicin MIC = 0.5 µg/mL).

Molecular hybridization in drug discovery is a promising approach where combining multiple bioactive components can create new conjugates with enhanced potency [32]. By merging covalently the benefits of each pharmacophoric element, the resulting hybrids may offer improved selectivity, varied mechanisms of action, reduced side effects, enhanced resistance to multiple drugs, and increased safety [33,34,35].

Given the biological properties of indazole and benzimidazole derivatives, and as an extension of our research work on the development of new bioactive aza-heterocycles [36,37,38,39], we herein report the design, synthetic access and characterization of a new series of indazole-benzimidazole hybrids **M1**–**M6** (Figure 2). These hybrids feature alkyl chain linkers bridging the two heterocyclic rings. The in vitro antimicrobial activity of **M1**–**M6** was assessed against four Gram-positive bacteria (*S. aureus*, *E. faecalis*, *B. cereus*, *L. plantarum*), four Gram-negative bacteria (*S. enteritidis*, *E. coli*, *C. coli*, *C. jejuni*) and four fungi (*S. cerevisiae*, *C. albicans*, *C. tropicalis*, *C. Glabrata*). In addition, in silico molecular docking study against the antimicrobial target protein beta-ketoacyl-ACP synthase III-malonyl-COA complex (PDB ID: 1HNJ) was performed to elucidate ligand-receptor interactions of **M1**–**M6** ligands and structure–activity relationship.

## 2. Results and Discussion

### 2.1. Chemistry

**Scheme 1:**
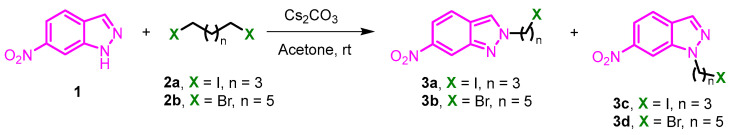
Synthesis of N-alkylated 6-nitroindazoles **3a**–**d**.

**Scheme 2:**
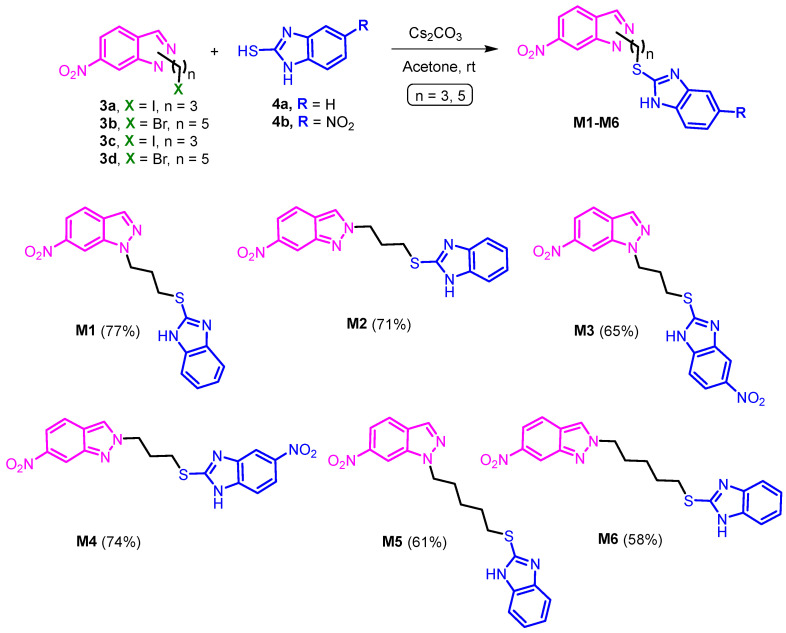
Synthetic route to prepare indazole–benzimidazole hybrids M1–M6.

The synthetic route to the new hybrids **M1**–**M6,** which required the preparation of *N*-alkylated 6-nitroindazoles **3a**–**d** (Scheme 1), is outlined in Scheme 2. These starting scaffolds were synthesized by alkylating 6-nitroindazole **1** with the appropriate 1,3- and 1,5-dihaloalkanes, according to the procedure depicted in Scheme 1. The 6-nitroindazole was effectively obtained by diazotization of 2-methyl-5-nitroaniline using the method described by Noelting [40]. Subsequently, the reaction of 6-nitroindazole with an excess of 1,3-diiodopropane or 1,5-dibromopentane in the presence of Cs_2_CO_3_ at room temperature afforded two main compounds. After chromatographic separation, these compounds were identified as the expected *N*-alkylated regioisomers **3a**/**3c** and **3b**/**3d**. The *N*2-alkylated derivatives **3a** and **3b** were isolated in yields of 34% and 28%, respectively, while the *N*1-alkylated derivatives **3c** and **3d** were isolated in yields of 58% and 52%, respectively. These results are consistent with our previously reported protocol [41].

The structures of all derivatives were unambiguously confirmed using 1D (^1^H and ^13^C spectra) and 2D [(^1^H, ^1^H) COSY, (^1^H, ^13^C) HSQC, and (^1^H, ^13^C) HMBC] NMR techniques, as well as mass spectrometry (Figures S1–S12). In the ^1^H-NMR spectra, the presence of bromopentyl and iodopropyl chains was readily confirmed by distinct signals in the aliphatic region. For isomers **3b** and **3d**, two triplets were observed, one at *ca* δ 3.4 ppm being assigned to the resonance of the CH_2_-Br protons and another at *ca* δ 4.5 ppm to the CH_2_ protons attached to the nitrogen atom. The remaining six CH_2_ protons appeared as three multiplets between 2.15 ppm and 1.46 ppm. Conversely, isomers **3a** and **3c** were found to display two triplets at *ca* δ 3.1 and 4.6 ppm ascribed to the CH_2_-I and *N*-CH_2_ protons, respectively, along with a multiplet at *ca* 2.5 ppm, due to the resonance of the remaining CH_2_ protons. In the ^13^C-NMR spectra, distinct signals were also observed in the aliphatic region, corresponding to the carbon resonances of the alkyl moieties. Isomers **3b** and **3d** showed five signals between *ca* δ 54.3 and δ 25.2 ppm, attributed to the five methylene carbons from the bromopentyl moiety. On the other hand, **3a** and **3c** were observed to exhibit three signals ranging from δ 54.1 and 2.1 ppm, corresponding to the three methylene carbons from the iodopropyl moiety.

Finally, the desired hybrids **M1**–**M6** were synthesized by nucleophilic substitution of each alkylated 6-nitroindazoles **3a**–**d** with the commercially available 2-mercaptobenzimidazole (**4a**) and 2-mercapto-5-nitrobenzimidazole (**4b**) (Scheme 2). These reactions were carried out in acetone at room temperature using Cs_2_CO_3_ as the base for 1.5 h. After the workup, the pure novel conjugates **M1**–**M6** were isolated by the conventional workup procedure, followed by recrystallization (petroleum ether) with yields ranging from 58% to 77%.

Structural confirmation of the hybrids was achieved by NMR and mass spectrometry (Figures S13–S50). All compounds show a peak in their mass spectra at the *m/z* value corresponding to the expected protonated molecular ion [M+H]^+^. The ^1^H-NMR spectra revealed a more complex profile in the aromatic region due to the resonance of the additional benzimidazole protons. In the aliphatic region, the profile is similar to that described for the precursors **3**. The most significant change is observed for the alkyl CH_2_ protons bonded to sulfur, which showed a downfield shift of approximately 0.2 ppm compared to those bonded to the related iodine group in the precursor. The number of carbon atoms in the ^13^C NMR spectrum further corroborated the assigned structure.

### 2.2. Crystal Structure Description

**Figure 3:**
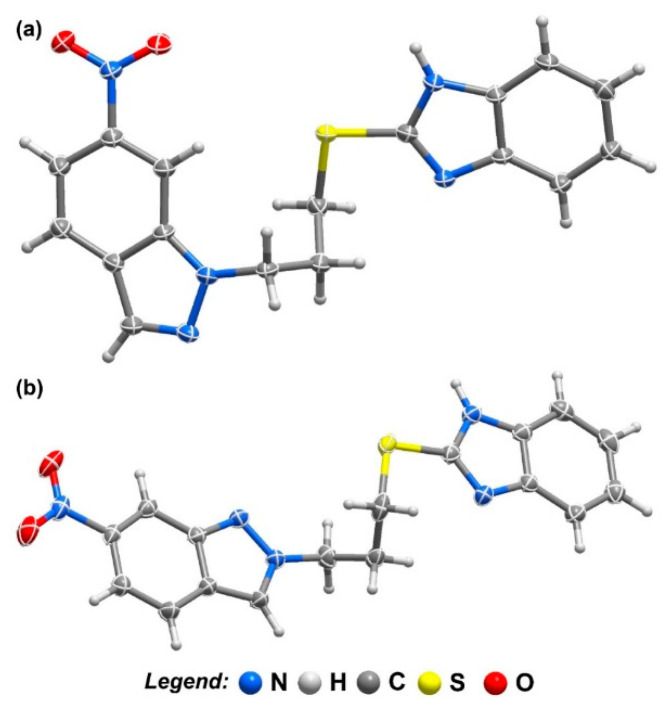
Molecular units present in compounds (**a**) **M1** and (**b**) **M2** obtained from single-crystal X-ray diffraction data. Non-hydrogen atoms are represented as ellipsoids drawn at the 50% probability level and hydrogen atoms are depicted as small spheres with arbitrary radii.

**Figure 4:**
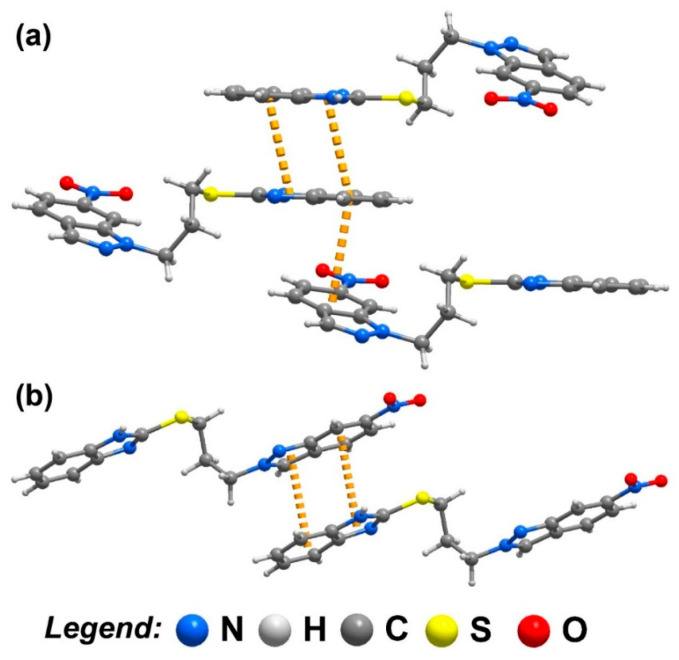
Schematic representation of the π-π interactions (dashed orange lines) present in compounds (**a**) **M1** and (**b**) **M2** (obtained from X-ray crystallographic data).

The crystal structures of compounds **M1** and **M2** were unequivocally determined by single-crystal X-ray diffraction. **M1** was found to crystallize in the centrosymmetric triclinic *P*-1 space group, with the asymmetric unit being composed of a whole molecular unit as depicted in Figure 3a. The close packing of this molecule was mediated by mostly weak intermolecular interactions, namely of the C–H···O kind [*d*_C···O_ found between 3.171(5) and 3.499(5) Å, and with <(CHO) interaction angles in the 122–156° range], and strong N–H···N contacts [*d*_N···N_ found at 2.943(4) Å with an <(NHN) interaction angle of 163°]. These supramolecular interactions are allied to strong π-π interactions between adjacent benzimidazole moieties, as depicted in Figure 4a (*d*_π···π_ found between ca. 3.55 and 3.86 Å).

Compound **M2**, on the other hand, was found to crystallize in the centrosymmetric monoclinic *P*2_1_/n space group, with the asymmetric unit also composed of a whole molecular unit (see Figure 3b). Because of the more linear nature of **M2** when compared to the previous compound, the close packing was mainly achieved by solely N–H···N interactions between adjacent indazole moieties [*d*_N···N_ found at 3.0340(16) Å with an <(NHN) interaction angle of 146°], as well as strong π-π interactions existing between the benzimidazole and indazole moieties as depicted in Figure 4b (*d*_π···π_ found between ca. 3.63 and 3.86 Å).

### 2.3. DFT Study

#### 2.3.1. Optimized Analysis

**Figure 5:**
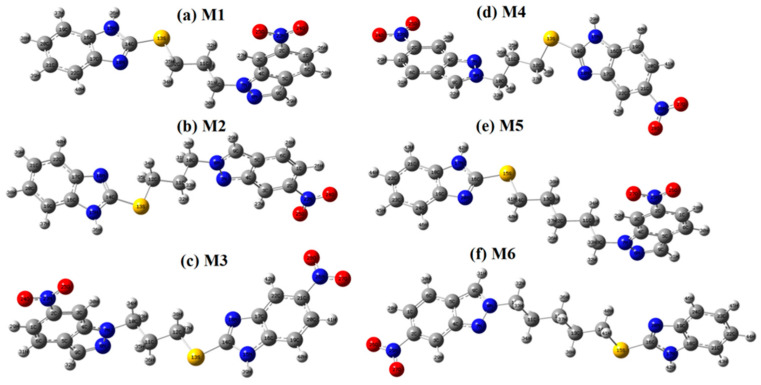
Optimized structures of **M1**–**M6** molecules.

The molecular geometries of **M1**–**M6** were optimized in the gas phase using the Gaussian 09W software package [42] and visualized through the GaussView 5.0 interface [43]. Geometry optimizations were carried out at the DFT/B3LYP level of theory [44,45], employing the 6-311++G(d,p) basis set [46,47,48], which is known for its accuracy in predicting electronic and structural properties. All quantum chemical calculations were carried out using neutral molecules in their ground-state singlet configuration. The optimized molecular structures are presented in Figure 5, and the corresponding total energy values, including electronic and zero-point energy contributions, are as follows: −1479.45784362, −1479.45255219, −1684.02274661, −1684.01733709, −1558.10812253, and −1558.10291724 a.u. for **M1** to **M6**, respectively. Geometry optimizations were performed starting from .cif-derived crystal geometries (**M1** and **M2**) or their crystallographically consistent modelled analogues (**M3**–**M6**), without imposing hypothetical all-trans conformations. Total energies are discussed only within each pair of constitutional isomers and not used to compare molecules with different chemical compositions. It is well established in the literature that gas-phase optimization may lead to slight deviations compared to experimentally observed solid-state structures [49,50,51]. These differences stem from intermolecular interactions present in the condensed phase, which are absent in isolated gas-phase calculations. However, the obtained gas-phase structures still provide valuable insights into the intrinsic stability and electronic properties of the molecules. A comparative analysis of the total energy values reveals that **M3** exhibits the lowest energy among the studied compounds. It is followed by its isomer **M4**, then by the pairs **M5**/**M6** and **M1**/**M2**. The energy differences within each pair are small but significant, with the *N*-1 isomer (**M1**, **M3**, **M5**) being more stable than its counterpart. For example, **M3** is about 14.2 kJ/mol more stable than **M4**. These results suggest that **M3** is thermodynamically more favorable, compared to the other structures, which aligns with fundamental quantum chemical principles of molecular stability.

#### 2.3.2. Frontier Molecular Orbital Analysis

**Figure 6:**
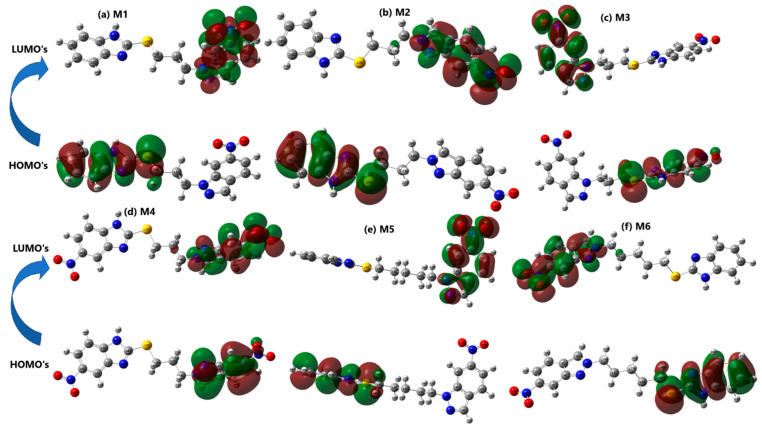
The HOMO-LUMO distributions of **M1**–**M6** molecules. Frontier molecular orbitals (HOMO and LUMO) are plotted at an isovalue of 0.02 a.u. using GaussView 5.0.

The frontier molecular orbitals (FMO), specifically the Highest Occupied Molecular Orbital (HOMO) and the Lowest Unoccupied Molecular Orbital (LUMO), play an important role in determining the reactivity, and selectivity of organic reactions, as well as the relative stability of reaction intermediates or products. The HOMO represents the highest energy level occupied by electrons and, in conjugated systems, is usually obtained from π orbitals. The LUMO is the lowest unoccupied energy level, typically corresponding to an antibonding molecular orbital. In chemical reactions, it plays a crucial role by accepting electrons during bond formation or cleavage [52,53,54]. To gain further insight into the electronic properties of the new hybrids **M1**–**M6**, their frontier orbitals were calculated in the gas phase using checkpoint (.chk) files generated from the optimized structures, using the same theory/functional and basis set. The resulting HOMO and LUMO shapes for the **M1**–**M6** molecules are presented in Figure 6. In addition, other global indicators (energy bandgap, ionization potential, electron affinity, chemical hardness, chemical softness, electronegativity, chemical potential, electrophilicity index and maximum charge transfer index) were calculated based on HOMO and LUMO energies in eV and are listed in Table 1. In the literature, small HOMO–LUMO energy gaps are generally associated with high chemical reactivity and low kinetic stability, due to more facile electronic transitions. In other words, when the energy difference between the HOMO and LUMO is small, electron transfer within the molecule can occur more readily. Such molecules are often referred to as ‘soft’, according to the principles of chemical hardness and softness [55].

An examination of Table 1 reveals that the HOMO-LUMO band gap (ΔE) is the highest for the **M4** molecule (3.893 eV) and the lowest for **M5** (3.043 eV). In general, a lower ΔE value indicates a softer (more reactive) molecule, meaning that electron transfer processes occur more easily. In this context, **M5** exhibits the highest chemical softness (0.329 eV) and the lowest hardness (1.522 eV), confirming that it is the most reactive molecule. Conversely, **M4** has the highest ΔE (3.893 eV) and hardness (1.947 eV), along with the lowest softness (0.257 eV). This suggests that **M4** is the most electronically stable and the least reactive molecule in the series. Considering chemical potential (μ) and electronegativity (χ), **M3** has the highest electronegativity (4.912 eV) and the lowest chemical potential (−4.912 eV), indicating that it exhibits the strongest electrophilic character among the studied molecules. Figure 6 presents the HOMO and LUMO distributions, showing how the orbitals are localized in different molecular regions. **M3** and **M5** exhibit the highest HOMO-LUMO orbital overlap, suggesting that these molecules may facilitate faster electron transitions, making them promising candidates for optoelectronic applications. From an electrophilicity index (ω) perspective, **M1** and **M5** have the highest values (6.628 eV and 6.592 eV, respectively), making them more susceptible to interacting with electrons. In contrast, **M6** has the lowest electrophilicity index (5.813 eV), suggesting a relatively lower tendency for such interactions. Regarding the maximum charge transfer capacity (ΔN_max_), **M5** has the highest value (2.944), indicating that it is the most favorable for charge transfer processes.

#### 2.3.3. MEP Analysis

**Figure 7:**
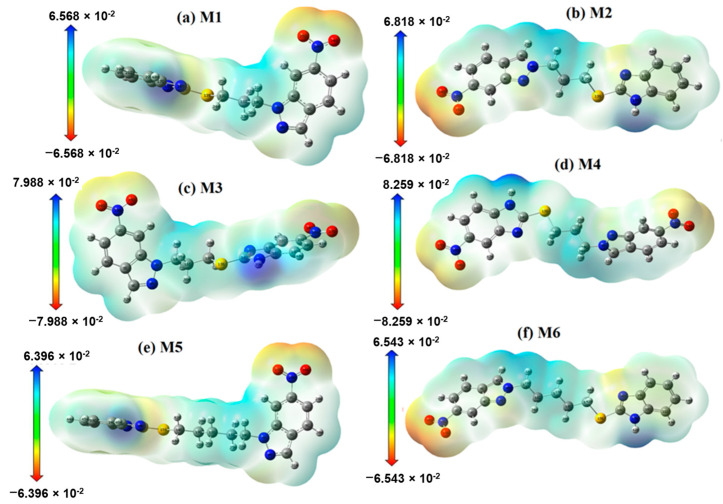
The MEP distributions of **M1**–**M6** molecules. MEP values are given in atomic units (a.u.), where 1 a.u. = 27.2114 eV.

Molecular electrostatic potential (MEP) reflects the intensity of interactions between adjacent charges, namely nuclei and electrons, at a given location. MEP maps provide critical insights into the electronic distribution and potential reactivity sites of a molecule and in biochemistry and pharmacology, and are widely used to determine distinctive patterns of positive and negative potentials, which can either stimulate or impede specific types of biological activity [56,57]. Considering this, the MEP of each molecule (**M1**–**M6**) was calculated in the gas phase using the .chk* files generated from their optimized structures, applying the same theory/functional and basis set. The resulting 3D-MEP surfaces for the **M1**–**M6** molecules are presented in Figure 7. As illustrated in this figure, the MEP surfaces exhibit distinct variations in electrostatic potential, ranging from highly negative (red regions) to highly positive (blue regions) areas. These variations are primarily influenced by the presence and position of electronegative atoms such as oxygen, nitrogen, and sulfur within the molecular structures. The MEP scale values obtained for each molecule were: −6.568 × 10^−2^ to 6.568 × 10^−2^ (**M1**), −6.818 × 10^−2^ to 6.818 × 10^−2^ (**M2**), −7.988 × 10^−2^ to 7.988 × 10^−2^ (**M3)**, −8.259 × 10^−2^ to 8.259 × 10^−2^ (**M4**), −6.396 × 10^−2^ to 6.396 × 10^−2^ (**M5**) and −6.543 × 10^−2^ to 6.543 × 10^−2^ (**M6**). The observed charge distribution in Figure 7 suggests that **M1**, **M3**, and **M5** molecules exhibit more pronounced electrophilic regions, which could make them potential candidates for electron-rich interactions, such as in nucleophilic addition reactions. Conversely, the relatively higher nucleophilic character of **M2**, **M4**, and **M6** suggests their preference for electrophilic attack, which may influence their chemical reactivity and intermolecular interactions. All MEPs were computed at the same theory level, and although GaussView assigns automatic scaling for each molecule’s visualization, the electrostatic potential values are quantitatively comparable. Furthermore, the analysis of MEP distributions provides valuable implications regarding the stability and potential applications of the molecules. The distinct charge separations observed in **M3** and **M5** suggest a higher tendency for strong intermolecular interactions, which may be beneficial for supramolecular assemblies or coordination chemistry. Additionally, considering that electrostatic interactions play a significant role in biological recognition processes, the observed variations in MEP distributions highlight the possibility of selective binding properties for these molecules. In particular, the strong electrophilic potential of **M3** may indicate enhanced interactions with nucleophilic biological targets, which could be further explored in computational docking studies for pharmaceutical applications. Comparing these findings with existing literature [56,57,58,59], the correlation between MEP distributions and chemical reactivity aligns well with previously reported studies on similar molecular frameworks. The strong negative electrostatic potential regions in certain molecules suggest that electron density is predominantly localized around specific functional groups, reinforcing their potential role in catalytic or redox-related processes. This study, therefore, contributes to a deeper understanding of the relationship between electrostatic properties and molecular stability, offering insights that could aid in the rational design of functionally active organic compounds.

### 2.4. Antimicrobial Activity

The increasing resistance of microorganisms to antibiotics highlights the critical need for synthesizing new antimicrobial compounds. Consequently, exploring novel compounds with antimicrobial properties has gained considerable attention recently [60,61]. In this study, the antibacterial potential of compounds **M1**–**M6** was qualitatively evaluated using the agar diffusion method. This approach provided a preliminary estimation of their potential to inhibit the growth of four Gram-positive bacteria (*S. aureus*, *E. faecalis*, *B. cereus*, *L. plantarum*) and four Gram-negative bacteria (*S. enteritidis*, *E. coli*, *C. coli*, *C. jejuni*).

The inhibitory zone diameter (IZD) values, as presented in Table S1, revealed that all the studied bacterial strains exhibited varying sensitivities to the analyzed compounds **M1**–**M6**. Compounds **M1** and **M4** demonstrated significant antibacterial activity against all tested Gram-positive bacteria, with IZDs ranging from 23 mm to 34 mm, compared to Tetracycline (TET) (IZDs = 23 mm to 29 mm) used as a reference antibiotic. Compounds **M2** and **M5** displayed similar inhibition, showing moderate sensitivity (IZD = 19 mm) against *B. cereus* ATCC 14579 and *L. plantarum growth*. Compounds **M3** and **M6** also demonstrated moderate sensitivity against *E. faecalis*, with IZDs of approximately 16 mm and 18 mm, respectively. In contrast, compounds **M5** and **M6** displayed weak growth inhibition of *L. plantarum* and *E. faecalis*.

For the inhibitory effect of the new hybrids against Gram-negative bacteria, compounds **M1**, **M3**, and **M5** demonstrated moderate inhibitory effects against *S. enteritidis*, displaying IZDs ranging from 15 mm to 19 mm. In addition, significant inhibition was observed against *E. coli*, with IZDs ranging from 20 mm to 29 mm for all the compounds analyzed. Regarding *C. coli* and *C. jejuni* species, compounds **M1** and **M3–M5** were significantly effective compared to TET, while compounds **M2** and **M6** moderately inhibited the growth of these bacterial species. This efficacy highlights the potential relevance of the compounds **M1**–**M6** in the treatment of foodborne infections caused by these *Campylobacter* species, which are responsible for various gastrointestinal diseases such as gastroenteritis and colitis, as well as severe complications including Guillain-Barre syndrome [62,63]. These results encourage further investigation to determine the underlying mechanisms of action, paving the way for developing more targeted treatments.

Regarding the antifungal potential of compounds **M1**–**M6**, their effectiveness was evaluated against six yeast species from the genera *Saccharomyces* and *Candida*, recognized for their medical importance as opportunistic pathogens in humans. The results reported in Table S2 revealed significant antifungal activity of compounds **M1**, **M3**, and **M6** against all tested yeasts with IZDs ranging from 20 mm to 35 mm, compared with Amphotericin B (AmB). In contrast, compound **M4** showed weaker inhibitory activity. Furthermore, compounds **M2** and **M5** exhibited moderate antifungal activity against *C. albicans* and *C. tropicalis*. These results suggest that compounds **M1**, **M3**, and **M6** could be considered promising candidates for developing new antifungal therapies, while compound **M4** would require further investigations to enhance its inhibitory potential.

The results of the antimicrobial screening of compounds **M1**–**M6** were corroborated by determining the corresponding minimum inhibitory concentrations (MICs), providing an accurate and complementary assessment of their efficacy against the microbial species studied. The analysis of the concentrations mentioned in Table 2 and Table 3 revealed that compounds inducing the widest IZDs have the lowest MICs. Certain compounds have demonstrated notable effectiveness at equivalent concentrations in inhibiting the growth of various microbial strains studied, performing comparably to Tetracycline (**TET)** and Ampicillin (**AmB)** used as reference drugs. At 3.90 μg/mL, the compounds demonstrated distinct antimicrobial activity. **M6** showed the broadest spectrum, inhibiting *S. aureus*, *B. cereus*, *S. enteritidis*, and *C. glabrata*. **M3** was active against *S. aureus* and *C. jejuni*, while **M1** and **M4** inhibited *L. plantarum*, with **M4** also active against *E. coli*. At 1.95 μg/mL, **M6** also showed activity towards *S. cerevisiae* and *C. tropicalis*, while **M3** inhibited *L. plantarum*, and **M1** was active against *C. jejuni*.

Determining minimum bactericidal concentrations (MBCs) and minimum fungicidal concentrations (MFCs) provides a complementary perspective on the ability of analyzed compounds **M1**–**M6** to act as inhibitors while highlighting their capacity to act as bactericidal and fungicidal agents. The analysis of the concentrations listed in Table 2 and Table 3 revealed that some of the analyzed compounds exhibited bactericidal and fungicidal activity at concentrations equal to the MICs.

Examples include **M6**, which exhibited both bactericidal and fungicidal effects at 3.90 μg/mL against *S. aureus* and *C. glabrata*. At the same concentration, **M1** exhibited a bactericidal effect against *L. plantarum* and **M4** against *E. coli*. At 7.81 μg/mL, **M1** displayed a fungicidal effect against *S. cerevisiae*, while at 15.62 μg/mL, **M3** demonstrated activity against *C. albicans*. At approximately 31.25 μg/mL, **M4** exhibited activity against *S. aureus* and **M5** against *E. coli* and *C. glabrata*. However, compounds **M1**, **M4**, and **M3** were effective at concentrations 7.81 μg/mL against *B. cereus* (**M1** and **M3**), *S. enteritidis* (**M4**), and *C. tropicalis* (**M3**).

### 2.5. Molecular Docking Studies

The molecular modeling computational studies were started by uploading the X-ray crystallographic structure of the beta-ketoacyl-ACP synthase III protein (PDB ID: 1HNJ) from the Protein Data Bank [64]. Protein and ligand preparation were then performed, followed by Induced Fit Docking (IFD), which accounts for the flexibility of both receptor and ligands, and optimization of the highest-scoring docking poses using Molecular Mechanics/Generalized Born Surface Area (MM-GB/SA) calculations to estimate binding energies. The active site of 1HNJ consists of a catalytic triad comprising acetylated Cys112, Asn274, and His244, consistent with the observations made by Qiu and coworkers [65]. The energetics derived from IFD and MM-GB/SA calculations for the candidate compounds **M1**–**M6** and the positive control inhibitors (Ampicillin and Tetracycline) bound to 1HNJ are presented in Table 4 and ranked based on their IFD scores.

For the newly designed series, a correlation between IFD and Glide eModel scores was generally observed, with lower eModel values corresponding to more favorable IFD scores, except for **M1** and **M4**. These two compounds deviated from the trend, suggesting that pose stability does not always reflect receptor adaptability.

The docking of ampicillin to 1HNJ generated eight poses, with the top-scoring one achieving a docking score of −13.464 kcal/mol and an IFD score of −1416.08 kcal/mol. The difference between the docking and the XP G scores arises from the oxidation state penalty attributed to the negative charge of this pose (Table 4). MM-GB/SA refinement of this pose gave a ΔG binding energy of −62.92 kcal/mol (Table 4). In the active site of beta-ketoacyl-ACP synthase III (Figure S51), its 3,3-dimethyl-7-oxo-4-thia-1-azabicyclo [3.2.0] heptane-2-carboxyl moiety is situated in the solvent-exposed region (Figure S51a), while the 6-([(2*R*)-2-amino-2-phenylacetyl]amino) unit is embedded within the catalytic binding site (Figure S51a). The electrostatic potential map indicates that in this region the amino acid residues are predominantly positively charged, facilitating a salt bridge interaction between Arg249 and the negatively charged carboxyl group of ampicillin (Figure S51a,b). Additionally, a hydrogen bond between Arg36, acting as a hydrogen bond donor, and the negatively charged carboxyl group of ampicillin is detected in the solvent-exposed region. Within the catalytic binding site, Asn247 establishes two hydrogen bonds with the carbonyl and the amino groups of ampicillin 6-([(2*R*)-2-amino-2-phenylacetyl]amino) moiety (Figure S51b).

IFD calculations for tetracycline binding to 1HNJ identified ten distinct conformations, with the highest-ranking conformation showing a docking score of −12.188 kcal/mol and an IFD score of −1415.52 kcal/mol. The difference between the docking and XPGlide scores arises from an oxidation state penalty linked to the negative charge of this pose. Prime MM-GB/SA refinement indicated an estimated binding free energy of −5.95 kcal/mol (Table 4). In the active site cavity of the beta-ketoacyl-ACP synthase III protein (Figure S52), the carboxamide group of tetracycline is located within the solvent-exposed region, while the 4-(dimethylamino)-1,6,10,11,12a-pentahydroxy-6-methyl-3,12-dioxo-4,4a,5,5a-tetrahydrotetracene is embedded within the catalytic binding site (Figure S52a). The electrostatic potential map indicates the presence of several positively charged amino acids in both the solvent-exposed region and the catalytic binding site, facilitating a salt bridge interaction between Arg249 and the negatively charged oxygen atom of tetracycline 10-hydroxy group (Figure S52a,b). Additionally, two hydrogen bonds are formed between Asn210, acting as a hydrogen bond donor, and the carbonyl groups of tetracycline in the solvent-exposed region. Within the catalytic binding site, a hydrogen bond is also evident between Asn247, serving as the hydrogen bond donor, and the oxygen atom of the 6-hydroxyl group of tetracycline (Figure S52b).

**Figure 8:**
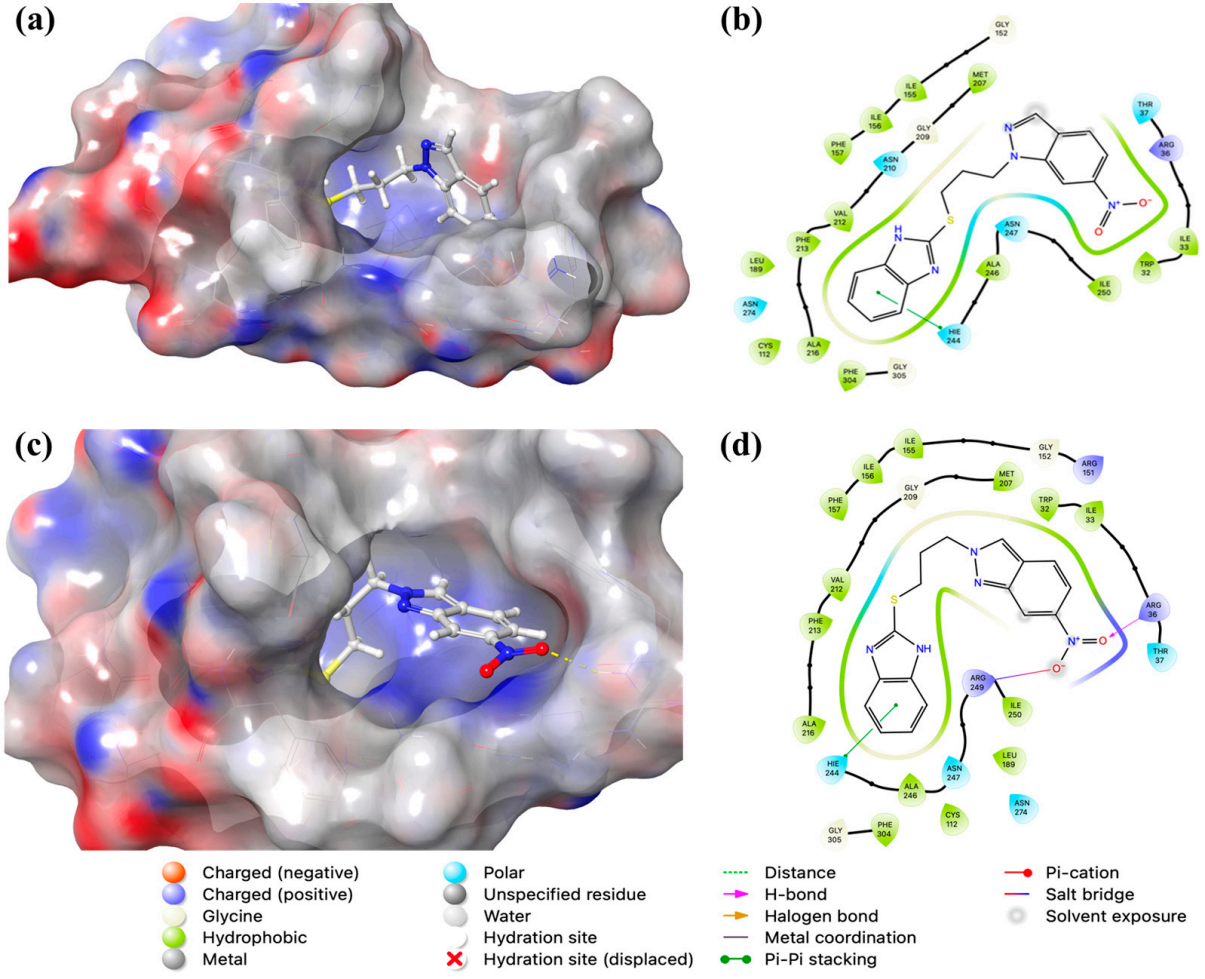
MM-GB/SA refined IFD poses of compounds **M1** and **M2** within the active site cavity of beta-ketoacyl-ACP synthase III protein (1HNJ). The electrostatic potential map of the protein’s active site is depicted, where blue spheres denote regions of low electronic density, red spheres indicate regions of high electronic density, and grey spheres represent hydrophobic regions. The ligands’ bound conformations are displayed using a ball and stick model, with grey denoting carbon, blue representing nitrogen, red for oxygen, and yellow for sulfur atoms. (**a**) The binding conformation of **M1** in the active site cavity of the beta-ketoacyl-ACP synthase III protein reveals that the 6-nitro-1*H*-indazole moiety of **M1** is situated in the solvent-exposed region. (**b**) The ligand interaction diagram of the **M1**-protein complex docking pose highlights significant π-π interactions, the solvent-exposed region of **M1**, and hydrophobic interactions. (**c**) The binding conformation of **M2** in the active site cavity of the beta-ketoacyl-ACP synthase III protein shows that the 6-nitro-2H-indazole moiety of **M2** is in the solvent-exposed region. (**d**) The ligand interaction diagram of the **M2**-protein complex docking pose emphasizes crucial hydrogen bonds, π-π interaction, the solvent-exposed region, and hydrophobic interactions.

For the new series of derivatives, IFD calculations for regioisomers **M1** and **M2** against the 1HNJ beta-ketoacyl-ACP synthase III protein yielded nine binding poses each. The highest scoring poses exhibited docking scores of −8.085 kcal/mol (**M1**) and −8.149 kcal/mol (**M2**) and IFD scores of −1409.01 kcal/mol and −1408.80 kcal/mol, respectively (Table 4). The discrepancies between the docking score and the XP G scores can be attributed to the oxidation state penalties imposed by the positive and negative charges of the nitro group in this pose, despite the compounds’ overall neutrality. MM-GB/SA refinement estimated a shift in ΔG binding energy of −28.34 kcal/mol for **M1** and −51.54 kcal/mol for **M2**, suggesting that **M1** is likely inactive, whereas **M2** may exhibit moderate activity against the beta-ketoacyl-ACP synthase III protein. In both compounds, the 6-nitro-indazole moieties are solvent-exposed, while the remaining regions occupy the catalytic site (Figure 8). The key interactions vary between the regioisomers **M1** and **M2**. For instance, the IFD pose of **M1** demonstrates a linear binding conformation (Figure 8b), whereas the IFD pose of **M2** displays a slightly folded binding conformation (Figure 8d). In **M1**, amino acids in both regions are predominantly hydrophobic, with no hydrogen bonds formed to the 6-nitro-1*H*-indazole moiety, and a π-π interaction occurs between the benzimidazole moiety and HIE244 in the catalytic site (Figure 8a,b). In **M2**, positively charged residues facilitate additional interactions: a salt bridge forms between Arg249 and the nitro oxygen, a hydrogen bond occurs between Arg34 and the neutral nitrogen of the 6-nitro-2*H*-indazole moiety, and a π-π interaction is observed between the benzimidazole moiety and HIE244 (Figure 8c,d). The salt bridge interaction and hydrogen bonding observed in **M2** are suggested to contribute to its slightly folded conformation. In contrast, these interactions are absent in **M1**, which consequently exhibits a linear conformation.

**Figure 9:**
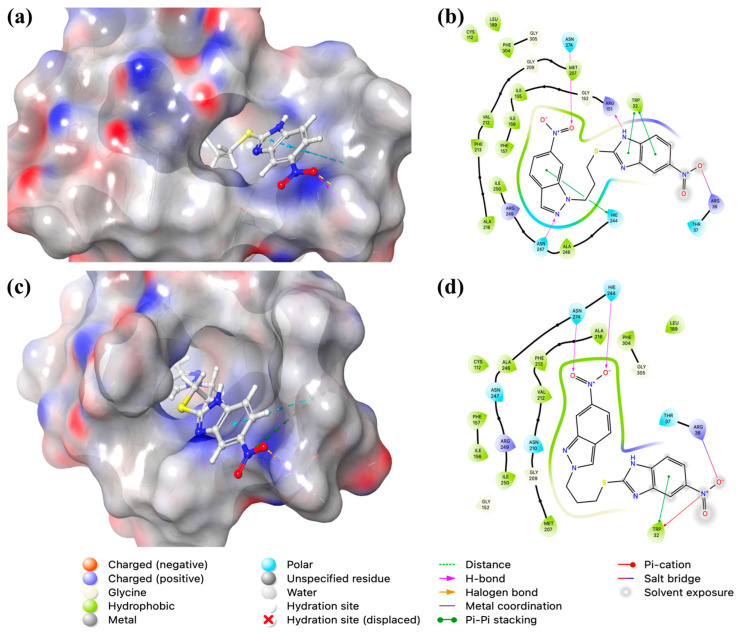
MM-GB/SA refined IFD poses of compounds **M3** and **M4** within the active site cavity of beta-ketoacyl-ACP synthase III protein (1HNJ). The electrostatic potential map of the protein’s active site is depicted, where blue spheres denote regions of low electronic density, red spheres indicate regions of high electronic density, and grey spheres represent hydrophobic regions. The ligands’ bound conformations are displayed using a ball and stick model, with grey denoting carbon, blue representing nitrogen, red for oxygen, and yellow for sulfur atoms. (**a**) The binding conformation of **M3** in the active site cavity of the beta-ketoacyl-ACP synthase III protein positions shows that the 6-nitro-1H-indazole moiety of **M3** is in the solvent-exposed region. (**b**) The ligand interaction diagram of the **M3**-protein complex docking pose identifies essential hydrogen bonds, the solvent-exposed region, salt bridge, π-π interactions, and hydrophobic interactions. (**c**) The binding conformation of **M4** in the active site cavity of the beta-ketoacyl-ACP synthase III protein locates the 6-nitro-2*H*-indazole moiety of **M4** in the solvent-exposed region. (**d**) The ligand interaction diagram of the **M4**-protein complex’s docking pose delineates significant salt bridge, π-π, π-cationic, and hydrophobic interactions.

IFD calculations produced 10 distinct poses for the ligand **M3** and 9 poses for its regioisomers **M4** in the complex with the protein 1HNJ. The poses with the highest score reported docking scores of −7.750 kcal/mol (**M3)** and −7.428 kcal/mol (**M4**), with IFD scores of −1410.57 and −1409.45 kcal/mol, respectively (Table 4). As with the other regioisomers, discrepancies between the docking score and the XP Glide scores are attributed to oxidation state penalties linked to the nitro groups. Further refinement of these poses through Prime MM-GB/SA calculations estimated binding free energies of −49.25 kcal/mol (**M3**) and −62.27 kcal/mol (**M4**), suggesting moderate activity for both compounds against the beta-ketoacyl-ACP synthase III enzyme. In both cases, the nitro-indazole moieties are positioned in the solvent-exposed region, while the remaining portions occupy the catalytic site (Figure 9). For **M3**, a salt bridge is evident between Arg36 and the negatively charged oxygen atom of the 6-nitro-1*H*-indazole moiety (Figure 9a,b). Within the catalytic site, a hydrogen bond occurs between Asn274 and the neutral nitrogen groups of the embedded 5-nitro-1*H*-benzo[*d*]imidazol-2-yl moiety. Another hydrogen bond is formed between Asn247, acting as donor, and the nitrogen of the **M3** indazole moiety (Figure 9b). In addition, π-π interactions between the indazole moiety in the solvent-exposed region and Trp32 are present. Finally, HIE244 engages in a π-π interaction with the 5-nitro-1*H*-benzo[*d*]imidazol-2-yl moiety located within the catalytic binding site. For **M4**, a salt bridge interaction between Arg36 and the negatively charged oxygen atom of the 6-nitro-2*H*-indazole moiety is evident (Figure 9c,d). A π-cationic interaction between Trp32 and the positively charged nitrogen atom of the 6-nitro-2*H*-indazole moiety embedded within the solvent-exposed region is also observed. In the catalytic binding site, a hydrogen bond forms between Asn247, serving as donor, and the nitrogen atom of the 5-nitro-1*H*-benzo[*d*]imidazol-2-yl moiety (Figure 9d). Furthermore, HIE244 interacts via a hydrogen bond with the negatively charged oxygen atom of the nitro group in the 5-nitro-1*H*-benzo[*d*]imidazol-2-yl moiety of **M4** located within the catalytic site.

**Figure 10:**
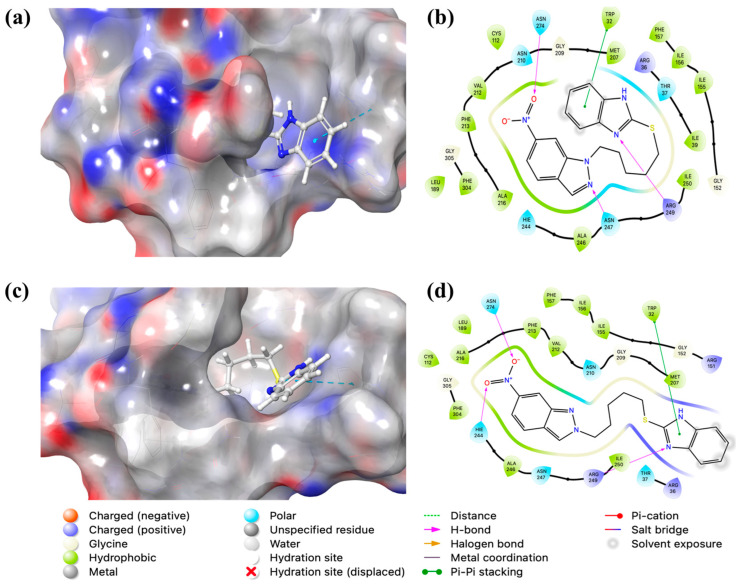
MM-GB/SA refined IFD poses compounds **M5** and **M6** within the active site cavity of beta-ketoacyl-ACP synthase III protein (1HNJ). The electrostatic potential map of the protein’s active site is depicted, where blue spheres denote regions of low electronic density, red spheres indicate regions of high electronic density, and grey spheres represent hydrophobic regions. The ligands’ bound conformations are displayed using a ball and stick model, with grey denoting carbon, blue representing nitrogen, red for oxygen, and yellow for sulfur atoms. (**a**) The binding conformation of **M5** in the active site cavity of the beta-ketoacyl-ACP synthase III protein locates the 6-nitro-1H-indazole moiety of M5 in the solvent-exposed region. (**b**) The ligand interaction diagram of the **M5**-protein complex docking pose features important hydrogen bonds, the solvent-exposed region, π-π and hydrophobic interactions. (**c**) The binding conformation of **M6** in the active site cavity of the beta-ketoacyl-ACP synthase III protein identifies the 6-nitro-1*H*-indazole moiety of **M6** as being in the solvent-exposed region. (**d**) The ligand interaction diagram of the **M6**-protein complex docking pose reveals significant hydrogen bonds, the solvent-exposed region, π-π interaction, and hydrophobic interactions.

For the remaining pair of regioisomers **M5** and **M6**, IFD calculations produced five distinct poses for both complexes with the protein 1HNJ. The poses with the highest score reported docking scores of −8.612 kcal/mol (**M5**) and −7.632 kcal/mol (**M6**), with IFD scores of −1409.68 and −1408.75 kcal/mol, respectively (Table 4). As before, discrepancies between docking and XP Glide scores are attributed to oxidation state penalties from the nitro groups, despite the overall neutrality of both compounds. Prime MM-GB/SA refinement estimated binding free energies of −82.85 kcal/mol for **M5** and −63.53 kcal/mol for **M6**, suggesting in this series a higher activity of **M5** against the beta-ketoacyl-ACP synthase III enzyme. In both compounds, the 1*H*-indazole moieties occupy the solvent-exposed region, while the remaining portions reside in the catalytic site (Figure 10). For **M5**, Arg249 interacts via a hydrogen bond with the nitrogen atom of the 1*H*-indazole moiety in the solvent-exposed region (Figure 10a,b), accompanied by a π-π interaction of the benzene ring with Trp32. Within the catalytic site, hydrogen bonds are observed between Asn247 and the nitrogen of the 5-nitro-1*H*-benzo[*d*]imidazol-2-yl moiety, and between Asn274 and the neutral oxygen of its nitro group. For **M6**, similar interactions are observed (Figure 10c,d), including a hydrogen bond between Arg249 and the indazole nitrogen in the solvent-exposed region, along with a π-π interaction with Trp32. Within the catalytic site, Asn274 interacts via a hydrogen bond with the nitrogen of the 5-nitro-1*H*-benzo[*d*]imidazol-2-yl moiety, while HIE244 interacts via a hydrogen bond with the oxygen atom of its nitro group. Notably, **M5** exhibits a conformation characterized by a slight folding, which is induced by the rotation of the five-carbon alkyl chain, resulting in the imidazole moieties adopting distinct conformations. Additionally, the amino acids within the active site cavities exhibit flexibility, contributing to the observed conformational change. Conversely, **M6** displays a linear conformation, which can be attributed to the hydrogen bond formation between Ile250 and the nitrogen atom of the 1*H*-indazole moiety.

The structure-activity relationship for this series of compounds (**M1**–**M6**) against beta-ketoacyl-ACP synthase III protein reveals that compounds **M1** and **M2** follow a similar binding mode. Both possess the 6-nitroindazole moiety in the solvent-exposed region, with a three-carbon alkyl chain linking it to the benzimidazole moiety, which is buried inside the active site cavity of 1HJN (Figure 8a–d). The MMGB/SA analysis suggests that both compounds show low predicted activity against beta-ketoacyl-ACP synthase III protein, which is apparent in the binding modes observed.

On the other hand, compounds **M3** and **M4** are distinct from the others due to the presence of an electron-withdrawing nitro group on both fused aromatic rings, linked by a three-carbon alkyl chain. Their binding modes differ from those of **M1** and **M2**, as the conformational change caused by the alkyl change enables the benzimidazole moiety to be positioned inside the catalytic binding site, where it forms hydrogen bonds with polar amino acids (Figure 9b). Therefore, compound **M3** is predicted to be moderately active against the beta-ketoacyl-ACP synthase III protein, as revealed by its binding modes. Conversely, **M4** is predicted to exhibit higher activity due to additional hydrogen bonds, complemented by salt bridge and π-π interaction.

Compounds **M5** and **M6** differ from the others by having a five-carbon chain between the 6-nitroindazole and benzimidazole moieties. Interestingly, the optimized IFD pose by MM-GB/SA for **M5** shows a high binding energy of −82.85 kcal/mol, while **M6** exhibits a more modest binding energy of −63.53 kcal/mol. Even though both compounds exhibit a similar binding mode, conformational differences between **M5** and **M6** in the solvent-exposed region likely account for the variation in the predicted activity.

## 3. Experimental Section

### 3.1. Generalities

Chromatographic purifications were performed on silica gel 60 (Merck, 0.063–0.200 mm). Analytical thin-layer chromatography (TLC) used silica gel 60 F_254_ plates (Merck, 0.063–0.200 mm) visualized under UV light at 254 nm and 365 nm. All solvents and reagents were purchased at analytical grades and used as received without further purification. NMR spectra (^1^H and ^13^C) were recorded on Bruker Avance 300 (at 300.13 MHz for ^1^H and at 75.47 MHz for ^13^C) and Avance 500 (^1^H at 500.13 MHz and ^13^C at 125.76 MHz) spectrometers using CDCl_3_ or CDCl_3_/CD_3_OD as solvent and tetramethylsilane (TMS) as the internal standard. Chemical shifts (δ) are reported in parts per million (ppm), and coupling constants (J) are given in hertz (Hz). Proton assignments were confirmed by 2D COSY experiments, while carbon resonances were assigned using HSQC and HMBC (optimized for a long-range coupling constant of 7 Hz). High-resolution mass spectra (HRMS) were obtained on a Micromass Q-Tof 2 instrument (Waters, Manchester, UK) operating in positive-ion mode using methanol sample solutions. Melting points were measured on a Büchi-Tottoli apparatus.

### 3.2. Chemical Procedures 

#### 3.2.1. General Procedure for Preparation of N-Alkylated 6-Nitroindazoles **3a**–**d**

To a solution of 6-nitroindazole (**1**) (100 mg, 0.62 mmol) and cesium carbonate (3 equiv.) in acetone 10 mL, 1,3-diiodopropane (**2a**) or 1,5-dibromopentane (**2b**) (1.1 equiv.) was added dropwise. The reaction was monitored by TLC. The resulting reaction mixture was stirred at room temperature for 1h. Following this, the solvent was evaporated, and the crude product was purified by column chromatography on silica gel, using a hexane:ethyl acetate mixture (7:3) as eluent.

**2-(3-iodopropyl)-6-nitro-2*H*-indazole, 3a**

Yellow solid. Yield: 34% (70 mg). M.p.: 284.3–286.2 °C. ^1^H NMR (300 MHz, CDCl_3_): δ 8.71–8.70 (m, 1H, H-7), 8.14 (d, *J* = 1.1 Hz, 1H, H-3), 7.94 (dd, *J* = 9.2, 1.9 Hz, 1H, H-5), 7.78 (d, *J* = 9.2 Hz, 1H, H-4), 4.64 (t, *J* = 6.4 Hz, 2H, *N*CH_2_-), 3.09 (t, *J* = 6.4 Hz, 2H, CH_2_I), 2.58–2.49 (m, *J* = 6.4 Hz, 2H, -CH_2_-) ppm. ^13^C NMR (75 MHz, CDCl_3_): δ 147.3, 146.7, 124.6, 124.1, 121.5, 116.0, 115.6, 54.1, 33.1, 2.1 ppm. MS-ESI(+): *m/z* 332.1 [M+H]^+^.

**2-(5-bromopentyl)-6-nitro-2H-indazole, 3b**

Yellow solid. Yield: 28% (54 mg). M.p.: 248.0–250.1 °C. ^1^H NMR (300 MHz, CDCl_3_): δ 8.71–8.70 (m, 1H, H-7), 8.05 (d, *J* = 0.7 Hz, 1H, H-3), 7.91 (dd, *J* = 9.2, 1.9 Hz, 1H, H-5), 7.76 (d, *J* = 9.2 Hz, 1H, H-4), 4.51 (t, *J* = 7.1 Hz, 2H, *N*CH_2_-), 3.40 (t, *J* = 6.6 Hz, 2H, -CH_2_Br), 2.15–2.05 (m, 2H, NCH_2_C*H*_2_), 1.96–1.87 (m, 2H, -C*H*_2_CH_2_Br), 1.56–1.46 (m, 2H, -CH_2_-) ppm. ^13^C NMR (75 MHz, CDCl_3_): δ 146.9, 146.5, 124.3, 123.7, 121.4, 115.8, 115.5, 54.3, 33.2, 32.0, 29.7, 25.2 ppm. MS-ESI(+): *m/z* 312.1 [M+H]^+^.

**1-(3-iodopropyl)-6-nitro-1H-indazole, 3c**

Yellow solid. Yield: 58% (119 mg). M.p.: 278.6–280.5 °C. ^1^H NMR (300 MHz, CDCl_3_): δ 8.52–8.50 (m, 1H, H-7), 8.15 (d, *J* = 1.0 Hz, 1H, H-3), 8.04 (dd, *J* = 8.9, 1.9 Hz, 1H, H-5), 7.85 (d, *J* = 8.9, 0.9 Hz, 1H, H-4), 4.60 (t, *J* = 6.4 Hz, 2H, *N*CH_2_-), 3.13 (t, *J* = 6.5 Hz, 2H, -CH_2_I), 2.55–2.46 (m, *J* = 6.5 Hz, 2H, -CH_2_-) ppm. ^13^C NMR (75 MHz, CDCl_3_): δ 146.6, 138.6, 134.0, 127.0, 122.0, 115.6, 105.9, 49.1, 33.1, 2.1 ppm. MS-ESI(+): *m/z* 312.1 [M+H]^+^.

**1-(5-bromopentyl)-6-nitro-1H-indazole, 3d**

Yellow solid. Yield: 52% (101 mg). M.p.: 280.6–282.1 °C. ^1^H NMR (300 MHz, CDCl_3_): δ 8.40–8.39 (m, 1H, H-7), 8.13 (d, *J* = 0.9 Hz, 1H, H-3), 8.03 (dd, *J* = 8.9, 1.9 Hz, 1H, H-5), 7.86 (dd, *J* = 8.9, 0.5 Hz, 1H, H-4), 4.49 (t, *J* = 7.1 Hz, 2H, *N*CH_2_-), 3.39 (t, *J* = 6.7 Hz, 2H, -CH_2_Br), 2.07–1.92 (m, 2H, *N*CH_2_C*H*_2_), 1.96–1.86 (m, 2H, -C*H*_2_CH_2_Br), 1.55–1.45 (m, *J* = 10.3, 6.4 Hz, 2H, -CH_2_-) ppm. ^13^C NMR (75 MHz, CDCl_3_): δ 146.5, 138.1, 133.4, 127.1, 122.0, 115.4, 105.8, 49.2, 33.2, 32.1, 29.0, 25.4 ppm. MS-ESI(+): *m/z* 232.2 [M-Br]^+^.

#### 3.2.2. General Procedure for the Preparation of the Hybrids **M1**–**M6**

A mixture of N-alkylated 6-nitroindazoles (3a-d) (0.3 mmol), cesium carbonate (1.1 equiv.) and mercaptobenzimidazole (4a) or 2-mercapto-5-nitrobenzimidazole (4b) (1 equiv.) in acetone (10 mL) was stirred at room temperature for 1.5 h. The reaction was monitored by TLC. Then, the reaction mixture was washed with water, and the desired product was extracted with diethyl ether. The organic layers were separated, dried over Na_2_SO_4_, and the solvent was evaporated under reduced pressure. The resulting residue was crystallized from petroleum ether, affording the pure compound.

**1-(3-((1*H*-benzo[*d*]imidazol-2-yl)thio)propyl)-6-nitro-1*H*-indazole, M1**

Yellow solid. Yield: 77% (82 mg). M.p.: 131.9–133.6 °C. ^1^H NMR (300 MHz, CDCl_3_): δ 8.44–8.43 (m, 1H, H-7), 8.17 (d, *J* = 0.9 Hz, 1H, H-3), 7.99 (dd, *J* = 8.8, 1.9 Hz, 1H, H-5), 7.84 (dd, *J* = 8.8, 0.7 Hz, 1H, H-4), 7.52–7.49 (m, 2H, H-4′ and H-7′), 7.23–7.20 (m, 2H, H-5′ and H-6′), 4.68 (t, *J* = 6.5 Hz, 2H, *N*CH_2_-), 3.35 (t, *J* = 6.8 Hz, 2H, -CH_2_S-), 2.57–2.48 (m, 2H, -CH_2_-) ppm.^13^C NMR (75 MHz, CDCl_3_): δ 149.3, 146.6, 138.4, 133.7, 127.0, 122.7, 121.7, 114.4, 114.2, 114.2, 114.1, 105.9, 47.2, 29.49, 29.46 ppm. MS-ESI(+): *m/z* 354.2 [M+H]^+^. HRMS-ESI(+): *m/z* calculated for C_17_H_15_N_5_O_2_S 354.1019 [M + H]^+^; found 354.1016.

**2-(3-((1*H*-benzo[*d*]imidazol-2-yl)thio)propyl)-6-nitro-2*H*-indazole, M2**

Yellow solid. Yield: 71% (75 mg). M.p.: 176.4–178.3 °C. ^1^H NMR (500 MHz, CDCl_3_): δ 8.72–8.71 (m, 1H, H-7), 8.15 (d, *J* = 0.9 Hz, 1H, H-3), 7.94 (dd, *J* = 9.1, 1.9 Hz, 1H, H-5), 7.78 (dd, *J* = 9.1, 0.9 Hz, 1H, H-4), 7.57–7.46 (m, 2H, H-4′ and H-7′), 7.25–7.23 (m, 2H, H-5′ and H-6′), 4.72 (t, *J* = 6.7 Hz, 2H, NCH_2_-), 3.35 (t, *J* = 6.7 Hz, 2H, -CH_2_S), 2.62–2.56 (m, 2H, -CH_2_-) ppm. ^13^C NMR (125 MHz, CDCl_3_): δ 149.2, 147.0, 146.8, 124.5, 124.3, 122.8, 121.6, 116.0, 115.3, 52.3, 30.4, 29.3 ppm. MS-ESI: *m/z* 354.2 [M+H]^+^. HRMS-ESI(+): *m/z* calculated for C_17_H_15_N_5_O_2_S 354.1019 [M + H]^+^; found 354.1017.

**6-nitro-1-(3-((5-nitro-1*H*-benzo[*d*]imidazol-2-yl)thio)propyl)-1*H*-indazole, M3**

Yellow solid. Yield: 65% (78 mg). M.p.: 159.5–161.4 °C. ^1^H NMR (500 MHz, CDCl_3_/CD_3_OD): δ 8.47 (s, 1H, H-7), 8.33 (s, 1H, H-4′), 8.18 (s, 1H, H-3), 8.12 (dd, *J* = 8.9, 2.1 Hz, 1H, H-5), 7.99 (dd, *J* = 8.9, 2.1 Hz, 1H, H-4), 7.87 (d, *J* = 8.9 Hz, 1H, H-6′), 7.57–7.1 (m, 1H, H-7′), 4.70 (t, *J* = 6.8 Hz, 2H, 2H, NCH_2_-), 3.33 (t, *J* = 6.8 Hz, 2H, -CH_2_S), 2.56–2.51 (m, 2H, -CH_2_-) ppm. ^13^C NMR (125 MHz, CDCl_3_/CD_3_OD): δ 146.6, 143.0, 138.4, 134.3, 126.9, 122.7, 118.1, 115.5, 105.8, 47.2, 29.4, 28.5 ppm. MS-ESI(+): *m/z* 399.1 [M+H]^+^. HRMS-ESI(+): *m/z* calculated for C_17_H_14_N_6_O_4_S 399.0870 [M + H]^+^; found 399.0866.

**6-nitro-2-(3-((5-nitro-1*H*-benzo[*d*]imidazol-2-yl)thio)propyl)-2*H*-indazole, M4**

Yellow solid. Yield: 74% (88 mg). M.p.: 164.6–166.7 °C. ^1^H NMR (300 MHz, CDCl_3_): δ 8.73 (s, 1H, H-7), 8.41 (s, 1H, H-4′), 8.19–8.16 (m, 2H, H-3 and H-6′), 7.96 (dd, *J* = 9.2, 2.0 Hz, 1H, H-5), 7.80 (d, *J* = 9.2 Hz, 1H, H-4), 7.61–7.44 (m, 1H, H-7′), 4.73 (t, *J* = 6.6 Hz, 2H, *N*CH_2_-), 3.42 (t, *J* = 6.7 Hz, 2H, -CH_2_S), 2.68–2.59 (m, 2H, -CH_2_-) ppm. ^13^C NMR (125 MHz, CDCl_3_): δ 147.0, 146.9, 143.7, 124.5, 124.3, 121.6, 118.6, 116.2, 115.2, 109.6, 105.7, 52.4, 30.5, 29.7 ppm. MS-ESI(+): *m/z* 399.1 [M+H]^+^. HRMS-ESI(+): *m/z* calculated for C_17_H_14_N_6_O_4_S 399.0870 [M + H]^+^; found 399.0866.

**1-(5-((1*H*-benzo[*d*]imidazol-2-yl)thio)pentyl)-6-nitro-1*H*-indazole, M5**

Yellow solid. Yield: 61% (70 mg). M.p.: 119.6–121.3 °C. ^1^H NMR (300 MHz, CDCl_3_): δ 8.38–8.37 (m, 1H, H-7), 8.11 (d, *J* = 1.1 Hz, 1H, H-3), 8.01 (dd, *J* = 8.9, 1.9 Hz, 1H, H-5), 7.83 (dd, *J* = 8.9, 0.7 Hz, 1H, H-4), 7.53–7.45 (m, 2H, H-4′ and H-7′), 7.22–7.17 (m, 2H, H-5′ and H-6′), 4.46 (t, *J* = 7.1 Hz, 2H, *N*CH_2_-), 3.29 (t, *J* = 7.1 Hz, 2H, -CH_2_S), 2.05–1.96 (m, 2H, NCH_2_C*H*_2_-), 1.87–1.77 (m, 2H, -C*H*_2_-CH_2_S), 1.53–1.45 (m, 2H, -CH_2_) ppm. ^13^C NMR (75 MHz, CDCl_3_): δ 150.0, 146.5, 138.1, 133.3, 127.0, 122.4, 122.0, 115.4, 105.9, 49.1, 32.4, 29.2, 29.0, 25.6 ppm. MS-ESI(+): *m/z* 382.2 [M+H]^+^. HRMS-ESI(+): *m/z* calculated for C_19_H_20_N_5_O_2_S 382.1332 [M + H]^+^; found 382.1328.

**2-(5-((1H-benzo[d]imidazol-2-yl)thio)pentyl)-6-nitro-2H-indazole, M6**

Yellow solid. Yield: 58% (66 mg). M.p.: 112.4–114.2 °C. ^1^H NMR (300 MHz, CDCl_3_): δ 8.38–8.37 (m, 1H, H-7), 8.11 (d, *J* = 1.2 Hz, 1H, H-3), 8.00 (dd, *J* = 8.8, 1.9 Hz, 1H, H-5), 7.83 (d, *J* = 8.8 Hz, 1H, H-4), 7.52–7.49 (m, 2H, H-4′ and H-7′), 7.23–7.17 (m, 2H, H-5′ and H-6′), 4.46 (t, *J* = 7.1 Hz, 2H, NCH_2_-), 3.29 (t, *J* = 7.1 Hz, 2H, -CH_2_S), 2.05–1.95 (m, 2H, *N*CH_2_C*H*_2_-), 1.87–1.77 (m, 2H, -C*H*_2_CH_2_S), 1.52–1.42 (m, 2H, -CH_2_-) ppm. ^13^C NMR (75 MHz, CDCl_3_): δ 150.0, 146.5, 138.1, 133.3, 127.0, 122.4, 122.0, 115.4, 105.8, 49.1, 32.4, 29.2, 29.0, 25.6 ppm. MS-ESI(+): *m/z* 382.2 [M+H]^+^. HRMS-ESI(+): *m/z* calculated for C_19_H_20_N_5_O_2_S 382.1332 [M + H]^+^; found 382.1329.

### 3.3. Single-Crystal X-Ray Diffraction Studies

Single crystals of compounds **M1** and **M2** were manually harvested from the crystallization vial and immersed in highly viscous FOMBLIN Y perfluoropolyether vacuum oil (LVAC 140/13, Sigma-Aldrich) to avoid degradation caused by the evaporation of the solvent [66]. Crystals were mounted on either Hampton Research CryoLoops or MiTeGen MicroLoops, typically with the help of a Stemi 2000 stereomicroscope equipped with Carl Zeiss lenses.

Crystal data for **M1** was collected using synchrotron radiation with wavelength λ = 0.69437 Å at 100 K [67]. The PILATUS@SNBL Hybrid Pixel Array Detector at the Swiss-Norwegian Beam Lines, European Synchrotron Radiation Facility [68] was used for the data collection. CrysAlisPro program package was employed for the single-crystal experimental data processing [69].

Crystal data for compound **M2** was collected at 150(2)K on a Bruker D8 QUEST equipped with Mo Kα sealed tube (λ = 0.71073 Å), a multilayer TRIUMPH X-ray mirror, a PHOTON III detector, and an Oxford Instruments Cryostrem 700+ Series low temperature device. Diffraction images were processed using the software package SAINT+ [70], and data were corrected for absorption by the multiscan semi-empirical method implemented in SADABS 2016/2 [71].

The structures were solved using the algorithm implemented in SHELXT-2014/5 [72], which allowed the immediate location of almost all of the heaviest atoms composing their molecular unit. The remaining missing and misplaced non-hydrogen atoms were located from difference Fourier maps calculated from successive full-matrix least-squares refinement cycles on *F*^2^ using the latest SHELXL from the 2018/3 release [73]. All structural refinements were performed using the graphical interface ShelXle [74].

Hydrogen atoms bound to carbon were placed at their idealized positions using *HFIX* instructions in SHELXL: *43* for aromatic carbon atoms and *23* for the –CH_2_ groups. These hydrogen atoms were included in subsequent refinement cycles with isotropic thermal displacements parameters (*U*_iso_) fixed at 1.2 × *U*_eq_ of the parent carbon atoms.

The last difference Fourier map synthesis showed: for compound **M1** the highest peak (0.719 eÅ^−3^) and the deepest hole (−0.913 eÅ^−3^) located at 0.75 and 0.80 Å from S1, respectively; for compound **M2** the highest peak (0.518 eÅ^−3^) and the deepest hole (−0.512 eÅ^−3^) located at 0.79 and 0.63 Å from S1, respectively. All structural drawings have been created using the software package Crystal Impact Diamond V3.2f [75].

Crystallographic data (including structure factors) for the two crystals structures have been deposited with the Cambridge Crystallographic Data Centre. Copies of the data can be obtained free of charge on application to CCDC, 12 Union Road, Cambridge CB2 2EZ, U.K. FAX: (+44) 1223 336033. E-mail: deposit@ccdc.cam.ac.uk.

Crystal data for **M1**: C_17_H_15_N_5_O_2_S, *M* = 353.40, triclinic, space group *P*-1, *Z* = 2, *a* = 8.6728(6) Å, *b* = 9.6699(8) Å, *c* = 10.7257(8) Å, *α* = 92.347(6)°, *β* = 98.401(6)°, *γ* = 114.658(7)°, *V* = 803.51(11) Å^3^, *μ*(Mo-Kα) = 0.208 mm^−1^, colourless plate with crystal size of 0.10 × 0.09 × 0.03 mm^3^. Of a total of 7952 reflections collected, 4089 were independent (*R*_int_ = 0.0939). Final *R*1 = 0.1008 [*I* > 2*σ*(*I*)] and *wR*2 = 0.3223 (all data). Data completeness to theta = 24.62°, 86.0%. CCDC 2471053.

Crystal data for **M2**: C_17_H_15_N_5_O_2_S, *M* = 353.40, monoclinic, space group *P*2_1_/*n*, *Z* = 4, *a* = 10.9967(11) Å, *b* = 10.1332(9) Å, *c* = 14.9104(13) Å, *β* = 107.529(3)°, *V* = 1584.3(3) Å^3^, *μ*(Mo-Kα) = 0.227 mm^−1^, orange block with crystal size of 0.17 × 0.15 × 0.10 mm^3^. Of a total of 41,473 reflections collected, 4261 were independent (*R*_int_ = 0.0324). Final *R*1 = 0.0370 [*I* > 2*σ*(*I*)] and *wR*2 = 0.1180 (all data). Data completeness to theta = 25.24°, 99.6%. CCDC 2471054.

### 3.4. DFT Details

In this study, the structural optimizations, frontier molecular orbital (HOMO-LUMO) distributions, and molecular electrostatic potential (MEP) analyses of the **M1**–**M6** molecules were conducted using the Gaussian 09W software package and visualized through the GaussView 5.0 interface. Density Functional Theory (DFT) calculations were performed employing the hybrid B3LYP exchange-correlation functional [44,45] in conjunction with the 6-311++G(d,p) basis set [46,47,48], which is well-regarded for providing reliable electronic structure predictions. The optimization process ensured that the molecular geometries correspond to the local energy minimum by verifying the absence of imaginary frequencies in the vibrational analysis. Conceptual DFT parameters, including ionization potential, electron affinity, electronegativity, chemical hardness, softness, chemical potential, electrophilicity index, and maximum charge transfer capability, were determined according to the formulations proposed by Parr and Pearson [76]. Following this, the electronic properties were examined by computing the HOMO-LUMO energy levels, which provide insights into molecular stability and reactivity. Additionally, the MEP surfaces were generated to elucidate the charge distribution and potential reactive sites within the studied molecules. Since theoretical calculations are performed in the gas phase, minor discrepancies between computed and experimental solid-state data are expected, as reported in previous theoretical studies. Nevertheless, such quantum chemical calculations offer valuable predictions regarding molecular behavior, reactivity trends, and electronic characteristics, thereby contributing to a deeper understanding of the studied compounds.

### 3.5. In Silico Details

All molecular modeling calculations were conducted utilizing Schrödinger’s Life-Sciences suite, version 2024-3 [77]. These computations were executed on a local host equipped with a MacBook Pro workstation featuring an Apple M2 Max chip and 32 GB of RAM. Access to the remote license server was facilitated through the Centre for High Performance Computing (CHPC) Lengau Cluster.

#### 3.5.1. Ligand Preparation

The structures of the ligands **M1**–**M6** were initially sketched in two dimensions using the Maestro software, specifically Schrödinger’s 2024-3 release [77]. Subsequent preparation of these structures as three-dimensional coordinates was accomplished with LigPrep [78]. Energy minimization employed the OPLS4 force field [79,80]. The ionization states of the compounds were simulated using Epik at a physiological pH of 7.4 ± 2. For each analog, all possible stereoisomeric and tautomeric forms were computed.

#### 3.5.2. Protein Preparation

The PDB structure of the antimicrobial protein receptor, beta-ketoacyl-ACP synthase III bound to malonyl-COA (PDB: 1HNJ), at a resolution of 1.46 Å, was retrieved from the RCSB Protein Data Bank. Preprocessing of the 3D structure of 1HNJ involved the assignment of bond orders and the completion of missing loops using Prime within the protein preparation wizard. Additionally, water molecule orientations were sampled, while hydrogen atoms in altered species were optimized based on crystal symmetry and PROPKA at pH 7.4 [81]. Waters located within a 5 Å radius of the ligands were removed, and comprehensive minimization of all atoms was executed with an Root Mean Square Deviation (RMSD) set at 0.30 Å using the OPLS4 force field [79,80].

#### 3.5.3. Induced Fit Docking (IFD)

An Induced Fit Docking protocol [82] was employed owing to the protein’s flexibility and to appropriately address potential induced fit effects. The IFD protocol was initiated by importing the pre-processed 3D structure of 1HNJ into the IFD panel on Maestro [77]. The co-crystallized ligand, malonyl-coenzyme A, was selected and restrained to designate the active site cavity centroid of 1HNJ. Subsequently, the candidate ligands **M1**–**M6** were chosen and incorporated into the IFD panel. The applied IFD protocol did not impose any constraints. Initial Glide docking and Prime refinement were performed with default settings. Additionally, a Glide redocking was conducted using the extra precision mode under default parameters.

#### 3.5.4. Molecular Mechanics Generalized Born Surface Area (MM-GB/SA) Calculations

For Prime [83] MM-GB/SA refinement, the top scoring IFD poses served as the starting structures. The MM-GB/SA method was implemented to predict the binding free energy change associated with the docking poses, with the variable dielectric surface generalized Born (VSGB) serving as the implicit solvation model. This procedure was succeeded by a minimization step utilizing the OPLS4 force field [79,80]. Protein flexibility was considered, particularly in amino acids situated within 5.5 Å of the ligands.

### 3.6. Antimicrobial Activity Assays

#### 3.6.1. Test Microorganisms

The bacterial and fungal strains selected for the antimicrobial assays were obtained from the American Type Culture Collection (ATCC) and from a variety of clinical and pathological samples supplied by the Laboratory of Bacteriology, Serology, and Hygiene at Ibn Sina University Hospital Center in Rabat, Morocco. A detailed list of these microbial species is provided in Table 5. The selection of these microorganisms aimed to encompass a broad spectrum of clinically significant strains, allowing for a comprehensive assessment of antimicrobial efficacy.

#### 3.6.2. Antimicrobial Screening

The antimicrobial activity of compounds **M1**–**M6** was assessed using the standard agar well-diffusion technique. Blood agar Base (Oxoid CM0271) enriched with Laked Horse Blood (Oxoid SR0048C) was employed for *Campylobacter* species, Mueller-Hinton (Oxoid CM0337) for other bacterial strains, and Sabouraud Dextrose (Oxoid CM0041) for yeasts species, in accordance with the Clinical Laboratory and Standards Institute guidelines [84]. Briefly, microbial suspensions were prepared in sterile physiological saline solution (0.9% NaCl, *w*/*v*) to achieve approximately 10^6^ CFU/mL for bacteria and 10^5^ CFU/mL for yeasts. These suspensions were uniformly spread on the respective agar surfaces. Wells of 5 mm diameter were made in the agar, and 50 µL of each compound at varying concentrations was introduced into each well. The plates were then incubated for 24 h at 37 °C for bacterial strains and at 30 °C for yeasts. After incubation, the diameters of the inhibition zones were recorded using a digital Vernier caliper (ZJchao 200 mm LCD, precision 0.02 mm) to quantify antimicrobial activity. Tetracycline (TET) and Amphotericin B (AmB) served as reference drugs for antibacterial and antifungal evaluations, respectively. Solvent controls were included in all assays, with 10% (*v*/*v*) DMSO used to dissolve the compounds; no inhibition zones were observed for DMSO alone, confirming that it did not affect microbial growth.

#### 3.6.3. Minimum Inhibitory (MIC), Bactericide (MBC) and Fungicide (MFC) Concentrations

The MICs of the compounds **M1**–**M6** were evaluated using the microdilution method in microplates, following the procedure described by Eloff [85]. In the first row of each microplate, 100 μL of broth medium [Campylobacter Enrichment Broth Base (Liofilchem REF 610131)] for *Campylobacter* species, Brain Heart Infusion (BHI) (Oxoid CM1135) for the other bacterial strains, and Sabouraud Dextrose Broth (SDB) (Oxoid CM0147) for yeast, was combined with 100 μL of each compound stock solution. Serial dilutions were then prepared along the row. Each well received 10 μL of each of the test microbial suspension, after which the plates were incubated for 24 h at 37 °C for bacteria and 30 °C for yeast strains. Following incubation, 10 μL of a freshly prepared solution of 3-(4,5-dimethylthiazol-2-yl)-2,5-diphenyl tetrazolium bromide (MTT, 0.4 mg/mL in sterile 0.9% NaCl *m*/*v*) was added to each well to visualize microbial viability. The plates were incubated again for 10 to 30 min at 37 °C. Wells exhibiting microbial growth developed blue-violet coloration. The MIC corresponded to the lowest compound concentration that completely inhibited visible microbial growth. Negative controls were prepared with 100 μL of 10% (*v*/*v*) DMSO instead of the tested compounds.

To determine the MBC and MFC values, 50 µL aliquots from wells showing no visible growth were subcultured onto nutrient agar (Oxoid CM0003) plates and incubated at 37 °C for 24 h. The lowest concentration that produced no microbial colonies was recorded as the MBC or MFC.

## 4. Conclusions

In this work, six novel indazole-benzimidazole hybrids **M1**–**M6** were synthesized, fully characterized and evaluated as antimicrobial agents. DFT optimization results confirmed that all molecular structures correspond to the local energy minimum, with **M3** exhibiting the lowest total energy, indicative of its thermodynamic stability. Additionally, frontier molecular orbital (FMO) analysis revealed that **M5** has the narrowest HOMO-LUMO bandgap (3.043 eV), suggesting its higher chemical reactivity, whereas **M4** displayed the largest energy gap (3.893 eV), consistent with greater kinetic stability and lower chemical reactivity. Investigation of antimicrobial activity showed that these compounds have potent antimicrobial activity. Remarkable antimicrobial activity was demonstrated by compound **M6** with MICs of 1.95 µg/mL against *S. cerevisiae* and *C. tropicalis*, and 3.90 µg/mL against *S. aureus*, *B. cereus* and *S. enteritidis*. Hybrid **M6** significantly outperformed the antimicrobial activity of the standards AmB and TET, which exhibited MIC values ranging from 15.62 to 62.50 µg/mL and 7.81 and 15.62 µg/mL, respectively. Molecular modeling data provided insight into the structure-activity relationship (SAR) and the binding modes influencing candidate compounds-protein recognition. Compounds **M1** and **M2** exhibited a distinct binding mode influenced by the nitro group at C6 of the indazole ring. Compounds **M3** and **M4**, which feature nitro substituents on both aromatic moieties and a propyl linker, yielded moderate estimated binding free energies. In contrast, compounds **M5** and **M6** have the same binding mode, but **M5** exhibits enhanced affinity due to conformational differences in its pentyl linker. It should be noted that the introduction of a nitro group at C5 of the polar benzimidazole moiety in **M4** enables additional hydrogen bonds with other polar side chains, contributing to increased predicted potency.

Future studies should include inserting functional groups into the solvent-exposed region that displaces unfavorable water molecules. Additionally, it will be interesting to explore the prospect of inserting R-groups into the site bound to polar amino acids in the catalytic binding site containing electron-donating functional groups. Furthermore, it would also be interesting to include heterocyclic groups as linkers to extend the chains. Finally, it would also be worth exploring the possibility of including a functional group in the benzimidazole moiety that will bring hydrogen bond with Cys112 to mimic the substrate binding mechanism to improve the binding affinities of the compounds in this series.

## Figures and Tables

**Table 1:** Some global reactivity descriptors computed for compounds **M1**–**M6**.

Parameters (eV)	M1	M2	M3	M4	M5	M6
*E*_LUMO_	−2.986	−2.770	−3.090	−2.866	−2.957	−2.734
*E*_HOMO_	−6.102	−6.180	−6.733	−6.760	−6.000	−6.058
Energy bandgap |*E*_HOMO_ − *E*_LUMO_|	3.115	3.410	3.643	3.893	3.043	3.325
Ionization potential (*I* = −*E*_HOMO_)	6.102	6.180	6.733	6.760	6.000	6.058
Electron affinity (*A* = −*E*_LUMO_)	2.986	2.770	3.090	2.866	2.957	2.734
Chemical hardness (*η* = (*I* − *A*)/2)	1.558	1.705	1.821	1.947	1.522	1.662
Chemical softness (*ζ* = 1/2*η*)	0.321	0.293	0.275	0.257	0.329	0.301
Electronegativity (*χ* = (*I* + *A*)/2)	4.544	4.475	4.912	4.813	4.479	4.396
Chemical potential (*μ* = −(*I* + *A*)/2)	−4.544	−4.475	−4.912	−4.813	−4.479	−4.396
Electrophilicity index *ω* = *μ*^2^/2*η*	6.628	5.871	6.622	5.949	6.592	5.813
Maximum charge transfer index (Δ*N_max_* = −*μ*/*η*)	2.917	2.624	2.697	2.472	2.944	2.644

**Table 2:** Minimum inhibitory concentration (MIC) and minimum bactericide concentration (MBC) (μg/mL) of the synthesized compounds **M1**–**M6** against pathogenic Gram-positive and Gram-negative bacteria ^a^.

Bacterial Strains			M1	M2	M3	M4	M5	M6	TET
Gram-positive	*S. aureus* ATCC 25923	MIC	15.62	125	3.90	31.25	250	3.90	15.62
		MBC	31.25	125	15.62	31.25	500	3.91	62.5
	*E. faecalis* ATCC 29212	MIC	15.62	62.5	125	7.812	125	62.5	15.62
		MBC	15.62	250	125	31.25	125	125	31.25
	*B. cereus* ATCC 14579	MIC	7.81	62.5	7.81	31.25	62.5	3.90	7.81
		MBC	31.25	625	31.25	125	500	7.81	15.62
	*L. plantarum* ATCC 14917	MIC	3.90	15.62	1.95	3.90	30.25	125	31.25
		MBC	3.90	62.5	7.812	15.63	30.25	125	62.5
Gram-negative	*S. enteritidis* ATCC 25928	MIC	250	31.25	250	7.81	62.5	3.90	7.81
		MBC	250	62.5	250	31.25	62.5	31.25	15.62
	*E. coli* ATCC 25922	MIC	250	250	62.5	3.90	31.25	15.62	7.81
		MBC	500	250	125	3.90	31.25	62.5	15.62
	*C. coli* ATCC 43478	MIC	7.81	250	31.25	62.5	7.81	250	3.90
		MBC	15.62	250	31.25	125	15.62	250	7.81
	*C. jejuni* ATCC 33560	MIC	1.95	125	3.90	15.62	31.25	125	15.62
		MBC	3.90	125	3.90	62.5	62.5	500	31.25

^a^—All values represent the experimentally determined endpoint concentrations. **TET**: Tetracycline (reference antibiotic control). MIC: Minimum Inhibitory Concentration. MBC: Minimum Bactericidal Concentration.

**Table 3:** Minimum inhibitory (MIC) and minimum fungicide (MFC) concentrations (μg/mL) of the synthesized compounds **M1**–**M6** against pathogenic yeasts ^a^.

Yeast Strains		M1	M2	M3	M4	M5	M6	AmB
*S. cerevisiae*	MIC	7.81	125	15.62	125	62.5	1.95	15.62
	MFC	7.81	250	62.5	125	62.5	7.81	62.5
*C. albicans*	MIC	31.25	125	15.62	250	125	7.81	7.81
	MFC	62.5	125	15.62	500	125	31.25	15.62
*C. tropicalis*	MIC	62.5	15.62	7.812	125	62.5	1.95	62.5
	MFC	125	31.25	31.25	125	125	7.81	250
*C. glabrata*	MIC	7.81	62.5	31.25	250	31.25	3.90	31.25
	MFC	15.62	62.5	125	250	31.25	3.90	62.5

^a^—All values represent the experimentally determined endpoint concentrations. **AmB**: Amphotericin B (reference antibiotic control). MIC: Minimum Inhibitory Concentration. MFC: Minimum Bactericidal Concentration.

**Table 4:** IFD and MM-GB/SA energetics results of the binding of candidate compounds against beta-ketoacyl-ACP synthase III protein.

Entry	Compound	Tot Q ^a^	XP G Score (kcal/mol)	Docking Score (kcal/mol)	Glide State Penalty (kcal/mol)	Glide eModel (kcal/mol)	IFD Score (kcal/mol)	MM-GB/SA ΔG_bind_ (kcal/mol)
1	**Ampicillin**	−1	−13.648	−13.464	0.184	−76.045	−1416.08	−62.92
2	**Tetracycline**	−1	−12.188	−10.844	1.344	−70.736	−1415.52	−5.95
3	**M3**	0	−8.597	−7.750	0.847	−90.859	−1410.57	−49.25
4	**M5**	0	−8.614	−8.612	0.002	−82.469	−1409.68	−82.85
5	**M4**	0	−8.275	−7.428	0.847	−79.922	−1409.45	−62.27
6	**M1**	0	−8.087	−8.085	0.002	−80.482	−1409.01	−28.34
7	**M2**	0	−8.151	−8.149	0.002	−75.915	−1408.8	−51.54
8	**M6**	0	−7.634	−7.632	0.002	−70.091	−1408.75	−63.53

^a^—Tot Q refers to the formal charge of the molecule.

**Table 5:** List of bacterial and fungal strains employed to evaluate the for antimicrobial properties of compounds **M1**–**M6**.

Microbial Strains			Source/Reference
Bacteria Strains	Gram +	*S. aureus*	ATCC 25923
		*E. faecalis*	ATCC 29212
		*B. cereus*	ATCC 14579
		*L. plantarum*	ATCC 14917
	Gram −	*S. enteritidis*	ATCC 25928
		*E. coli*	ATCC 25922
		*C. coli*	ATCC 43478
		*C. jejuni*	ATCC 33560
Yeast species		*S. cerevisiae*	ATCC 9763
		*C. albicans*	Cutaneous infection
		*C. tropicalis*	Cutaneous infection
		*C. glabrata*	Cutaneous infection

## Data Availability

The original contributions presented in this study are included in the article/Supplementary Material. Further inquiries can be directed to the corresponding author(s).

## Supplementary Materials

The following supporting information can be downloaded at: https://www.mdpi.com/article/10.3390/antibiotics14111150/s1, Figures S1–S50 present the NMR spectroscopy and mass spectrometry data of compounds **3a**–**d** and **M1**–**M6**. Figures S51 and S52 show the MM-GB/SA-refined IFD configurations of ampicillin (**AmB**) and tetracycline (**TET**), respectively, within the active site cavity of 1HNJ. Tables S1 describe the diameters of the inhibition zones (mm) [86] for compounds **M1**–**M6** against pathogenic Gram-positive and Gram-negative bacteria, while Table S2 presents their inhibition against pathogenic yeasts.
