# Supplementary material for: Synthesis, Characterization, Antimicrobial Activity and Molecular Modeling Studies of Novel Indazole-Benzimidazole Hybrids
Source: Antibiotics (Basel). 2025 Nov 13;14(11):1150. doi: 10.3390/antibiotics14111150 (PMC12649635; doi:10.3390/antibiotics14111150)
Supplement: Supplementary file 1 [file antibiotics-14-01150-s001.zip › antibiotics-3948424-supplementary.pdf]

Supporting Information

Synthesis, characterization, antimicrobial activity and molecular modeling studies of novel indazole-benzimidazole hybrids

Redouane Er-raqioui [1,2,3], Sara Roudani [1,3], Imane El Houssni [4], Njabulo J. Gumede [5,*], Yusuf Sert [6], Ricardo F. Mendes [7], Dimitry Chernyshov [8], Filipe A. A. Paz [7], José A. S. Cavaleiro [2], M. Amparo F. Faustino [2], Rakib El Mostapha [1,3,*], Said Abouricha,[1] Khalid Karrouchi [9,*], M. Graça P. M. S. Neves [2], Nuno M. M. Moura [2,*]

[1] Laboratory of Molecular Chemistry Materials and Catalysis, Faculty of Sciences and Technics, Sultan Moulay Slimane University, B.P. 523, 23000, Beni-Mellal, Morocco.

[2] LAQV-REQUIMTE, Department of Chemistry, University of Aveiro, Campus Universitário de Santiago, 3810-193 Aveiro, Portugal.

[3] Higher School of Technology, Sultan Moulay Slimane University, B.P.336, 23200, Fkih Ben Salah 23000, Morocco.

[4] BVPRT, BER Center, Faculty of Sciences, Mohammed V University, Rabat, Morocco.

[5] Department of Chemical and Physical Sciences, Faculty of Natural Sciences, Walter Sisulu University (WSU), Private Bag X01, Mthatha, Eastern Cape 4099.

[6] Bozok University Department of Physics Yozgat, Turkey.

[7] CICECO – Aveiro Institute of Materials, Department of Chemistry, University of Aveiro, Campus Universitário de Santiago, 3810-193 Aveiro, Portugal.

[8] European Synchrotron Radiation Facility, SNBL CS40220 38043 Grenoble CEDEX 9, France.

[9] Laboratory of Analytical Chemistry and Bromatology, Faculty of Medicine and Pharmacy, Mohammed V University in Rabat, Morocco.

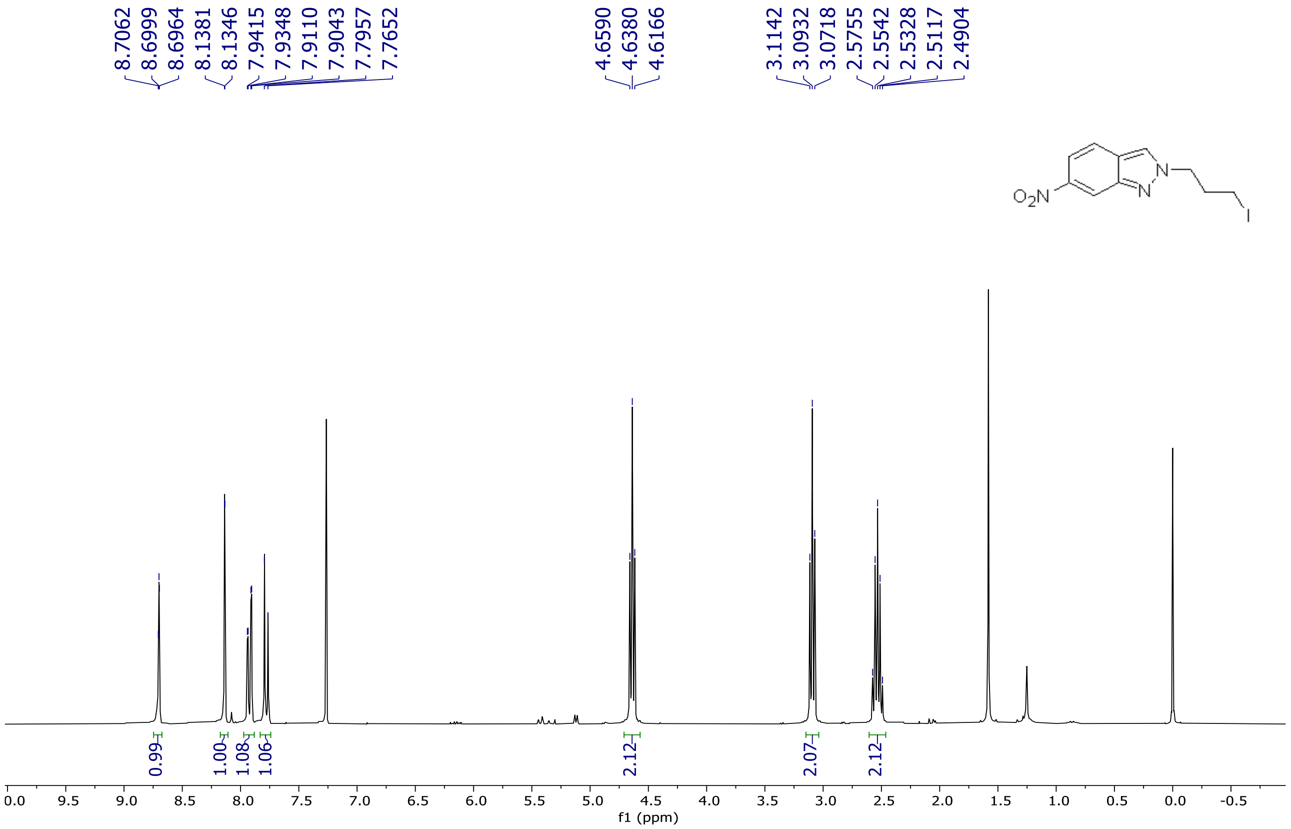

Figure S1. ^{1}H NMR spectrum of compound **3a** in $CDCl_3$.

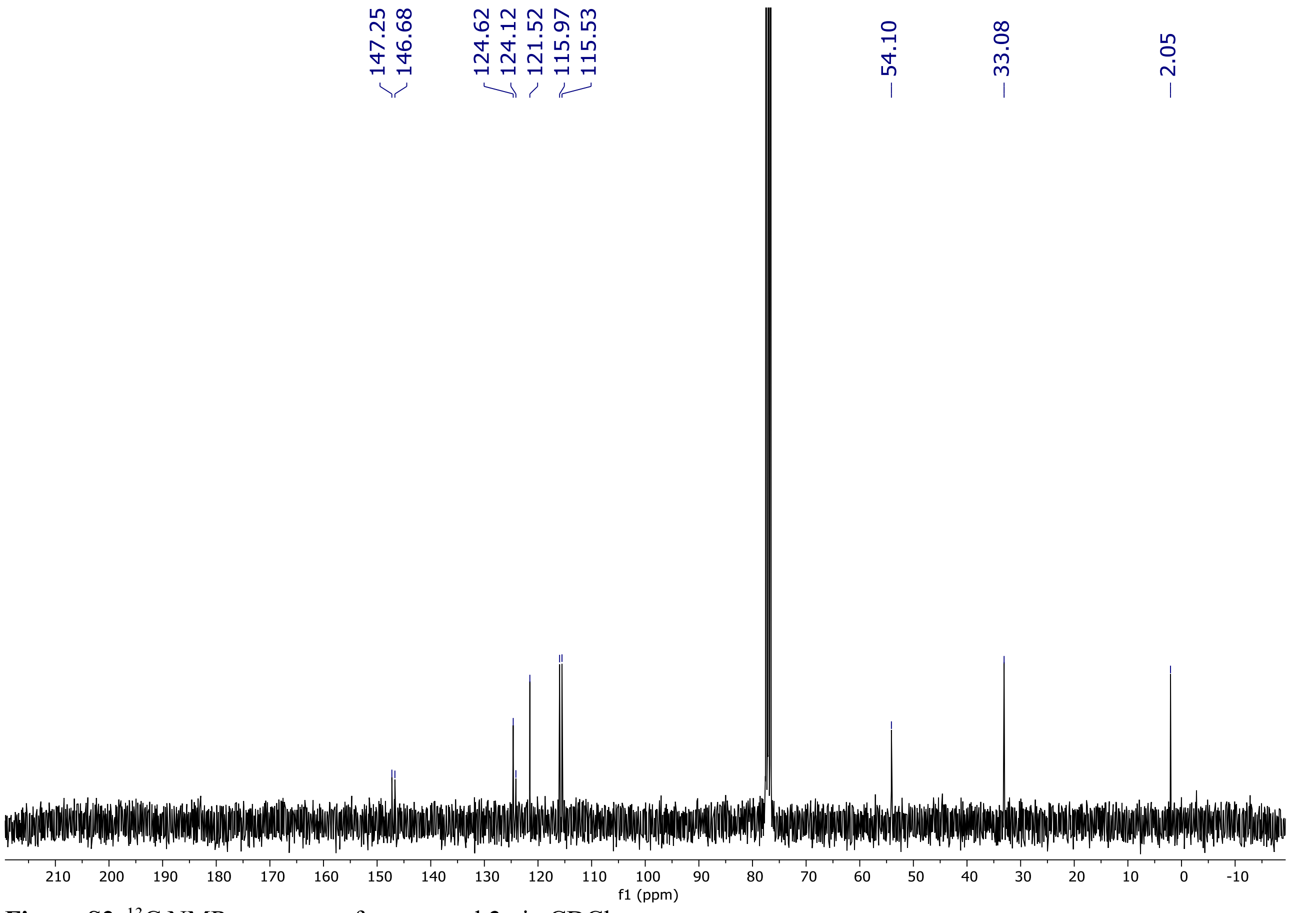

Figure S2. ^{13}C NMR spectrum of compound **3a** in $CDCl_3$.

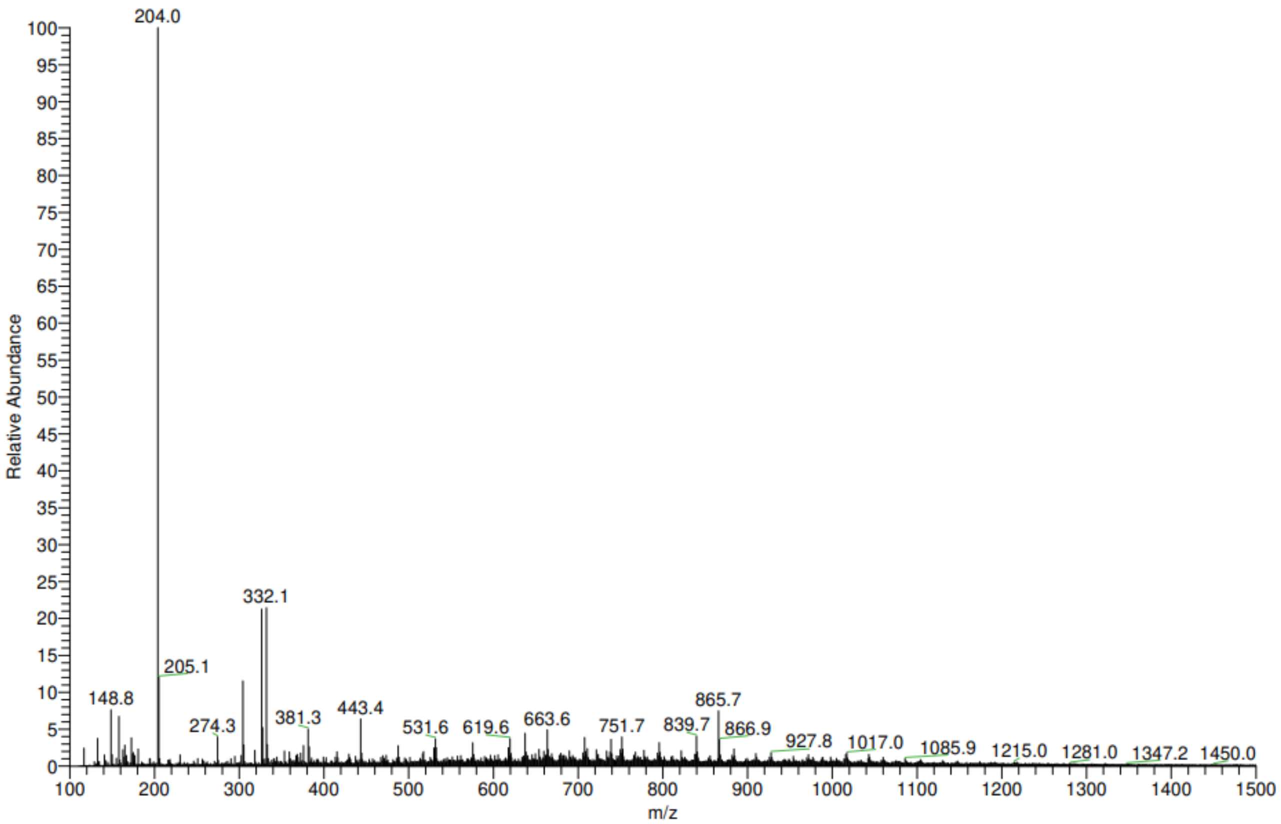

Figure S3. MS-ESI(+) spectrum of compound **3a**.

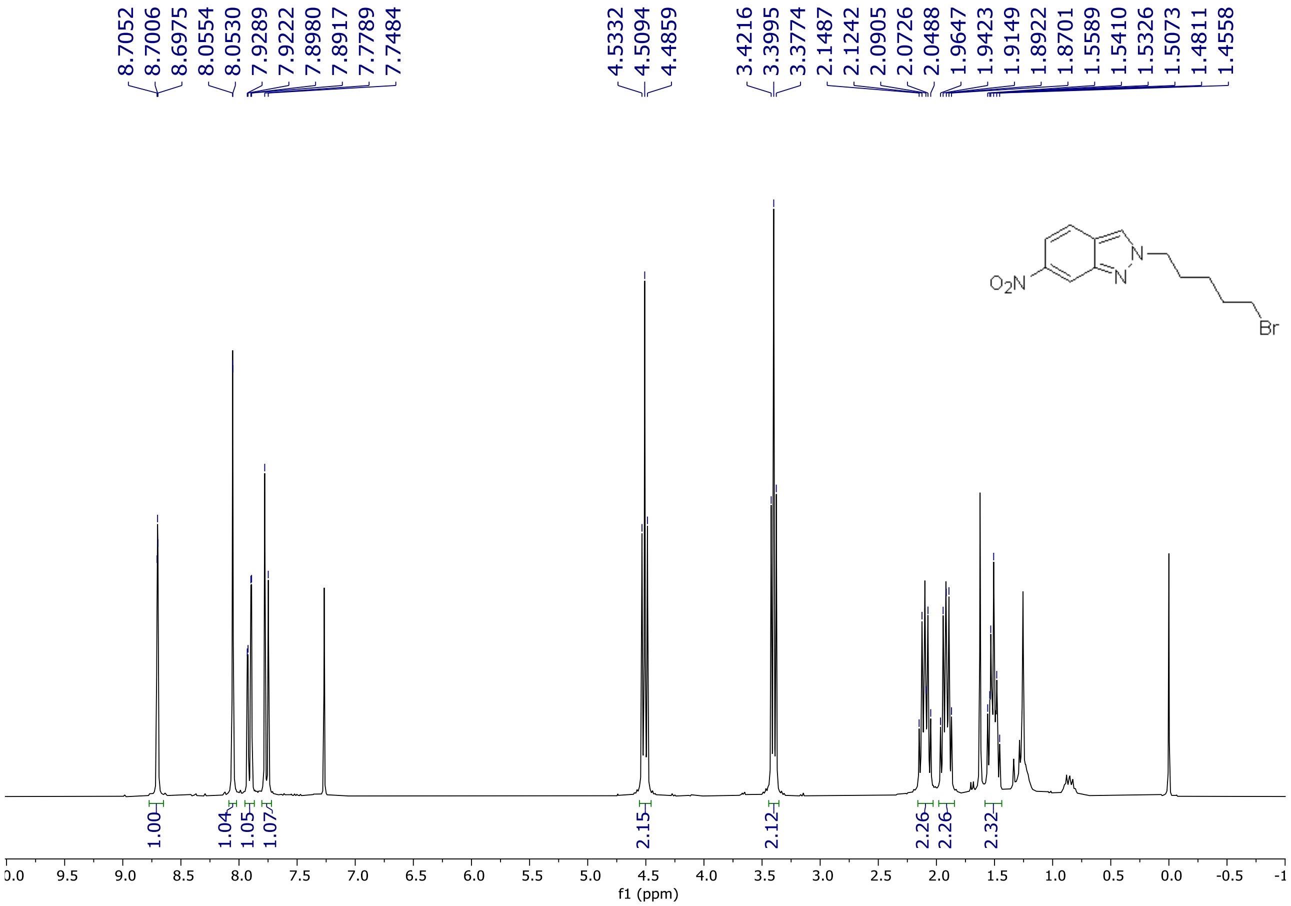

Figure S4. ^{1}H NMR spectrum of compound **3b** in $CDCl_3$.

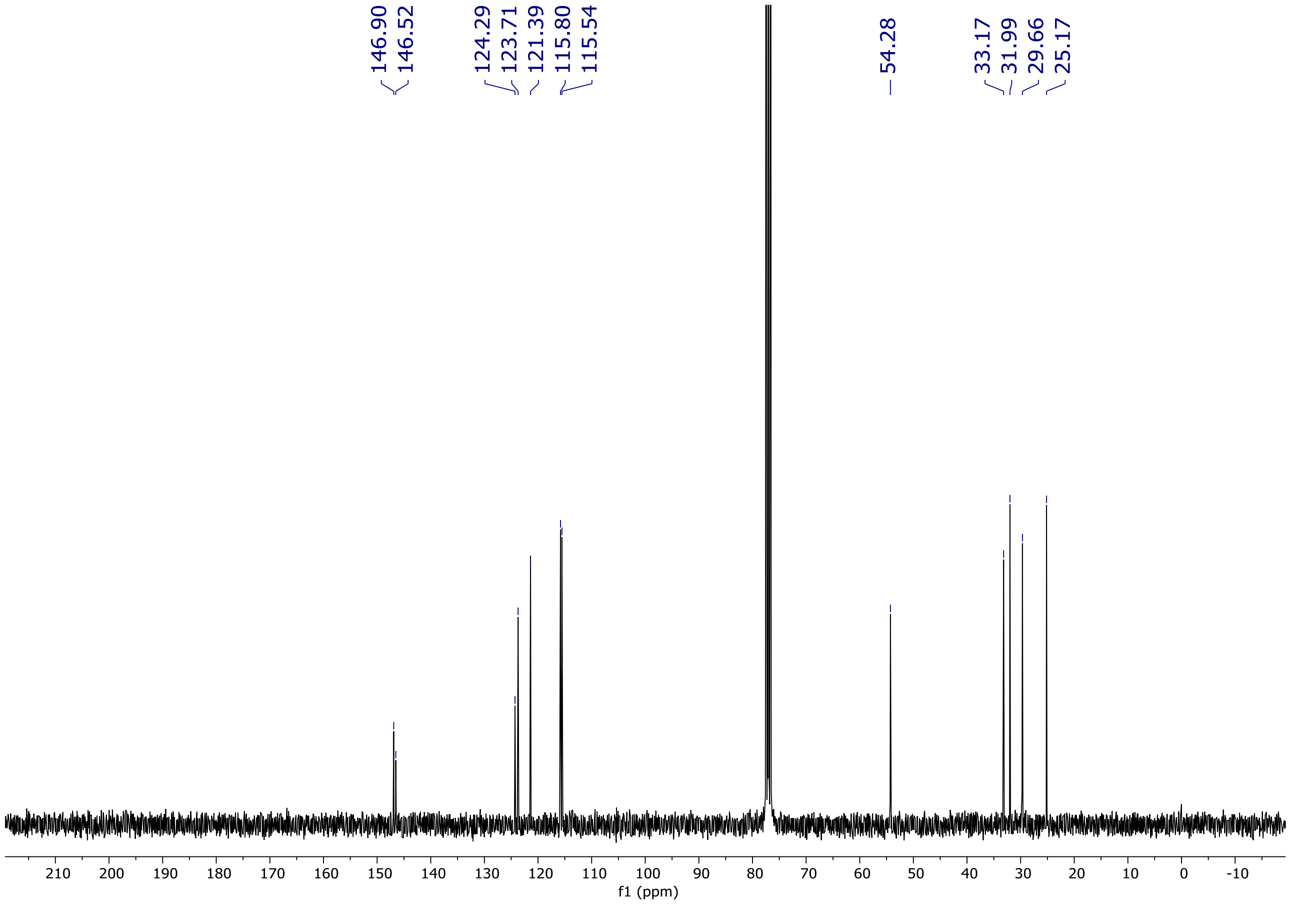

Figure S5. ^{13}C NMR spectrum of compound **3b** in $CDCl_3$.

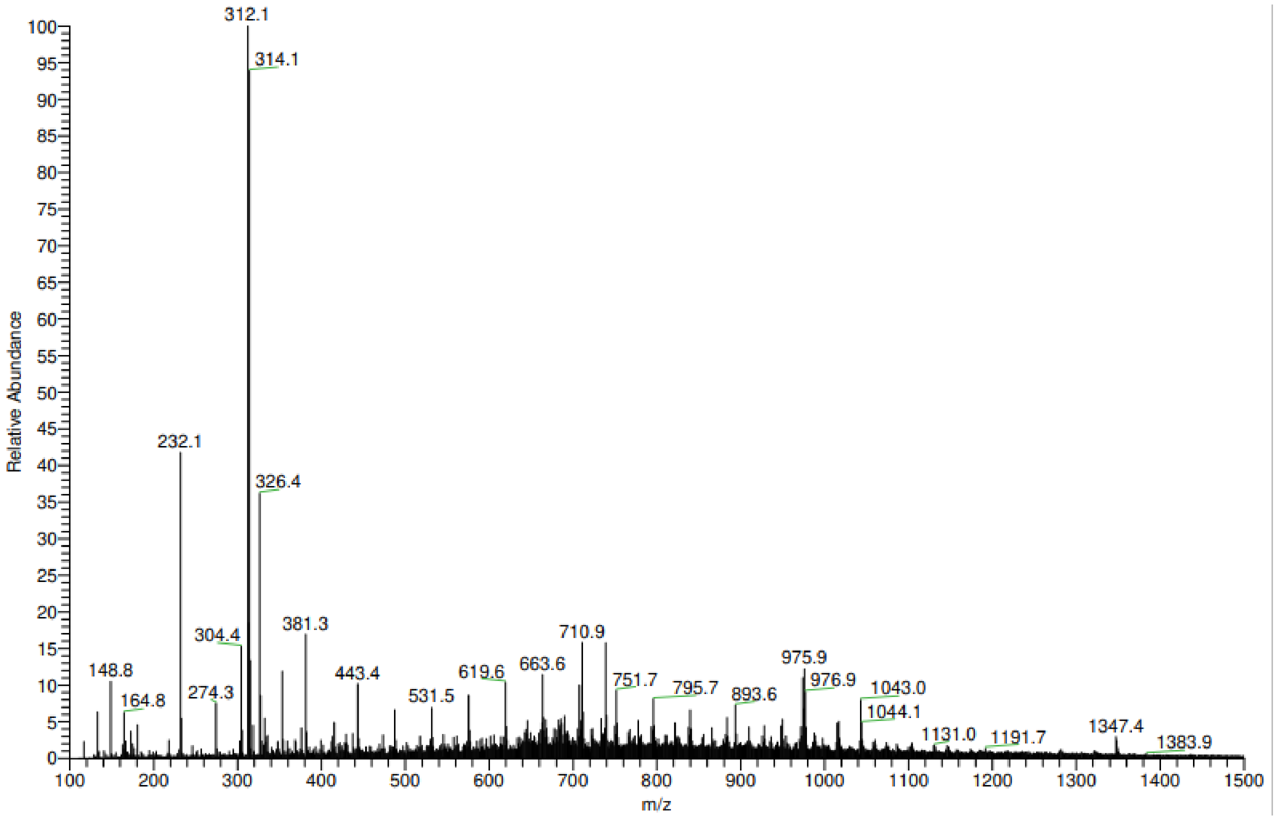

Figure S6. MS-ESI(+) spectrum of compound **3b**.

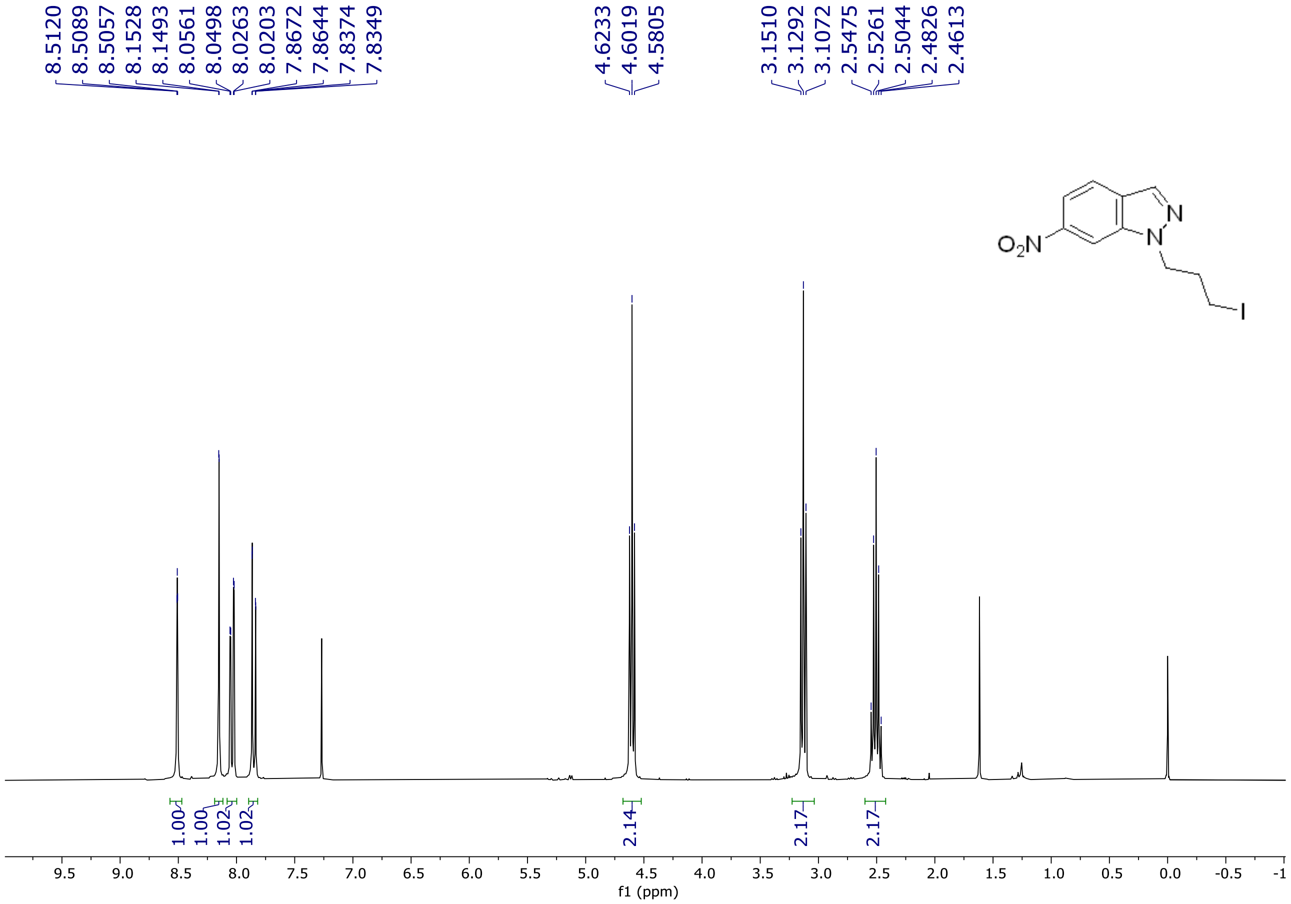

Figure S7. ^{1}H NMR spectrum of compound **3c** in $CDCl_3$.

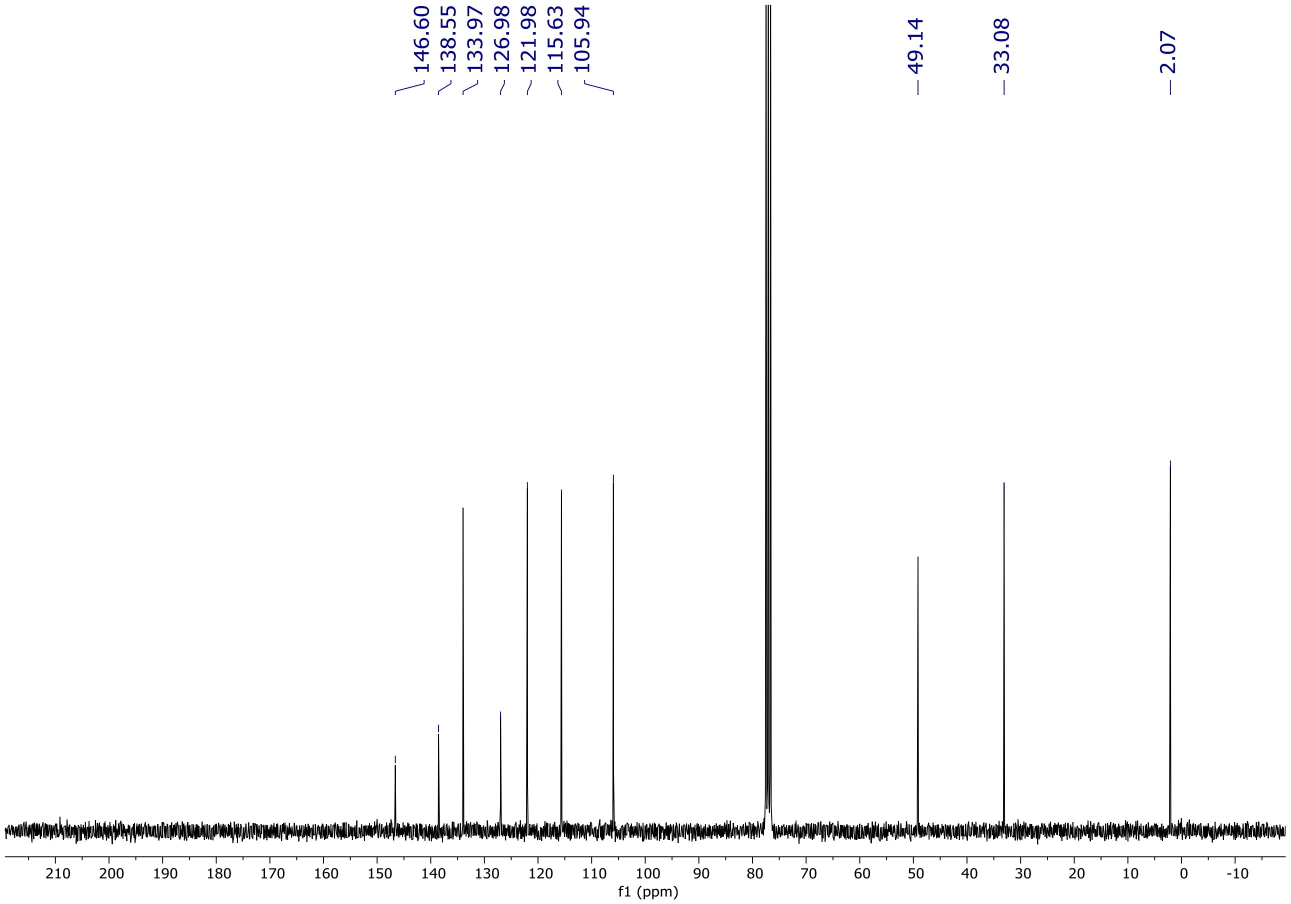

Figure S8. ^{13}C NMR spectrum of compound **3c** in $CDCl_3$.

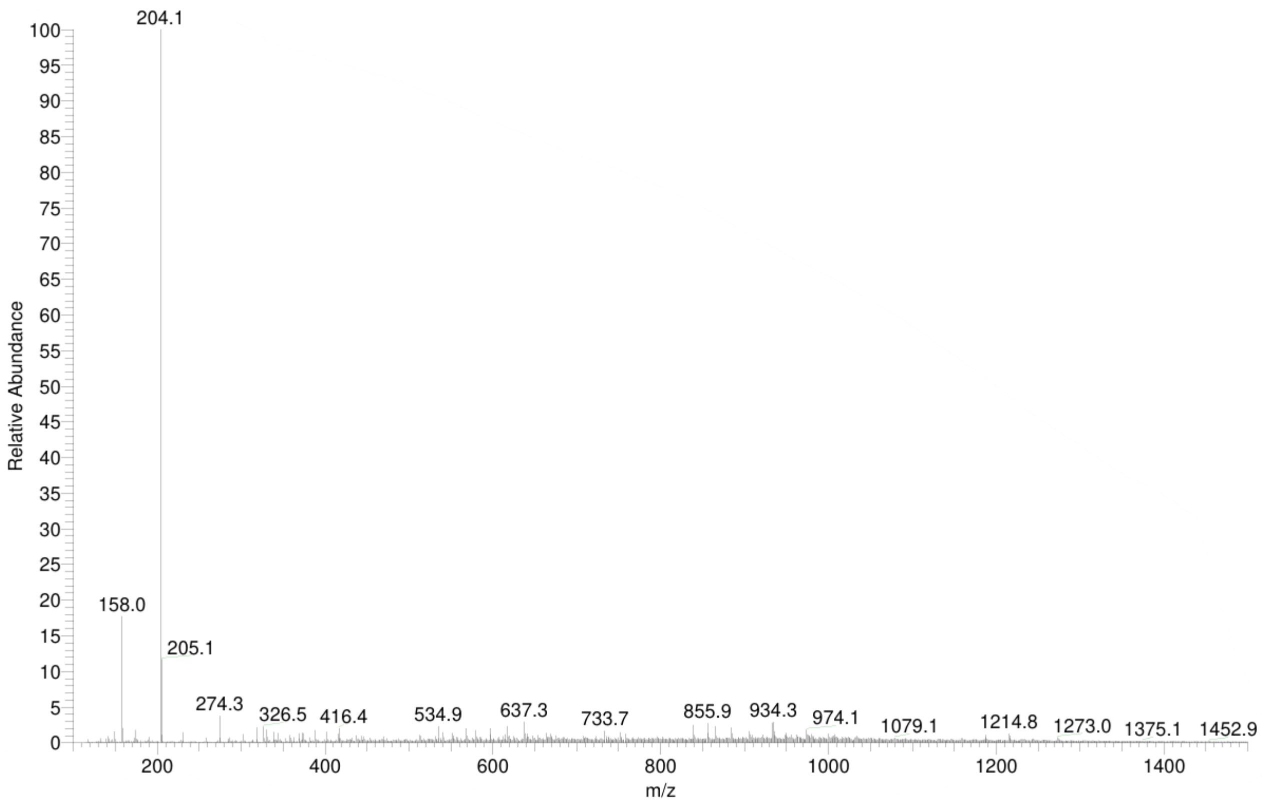

Figure S9. MS-ESI(+) spectrum of compound **3c**.

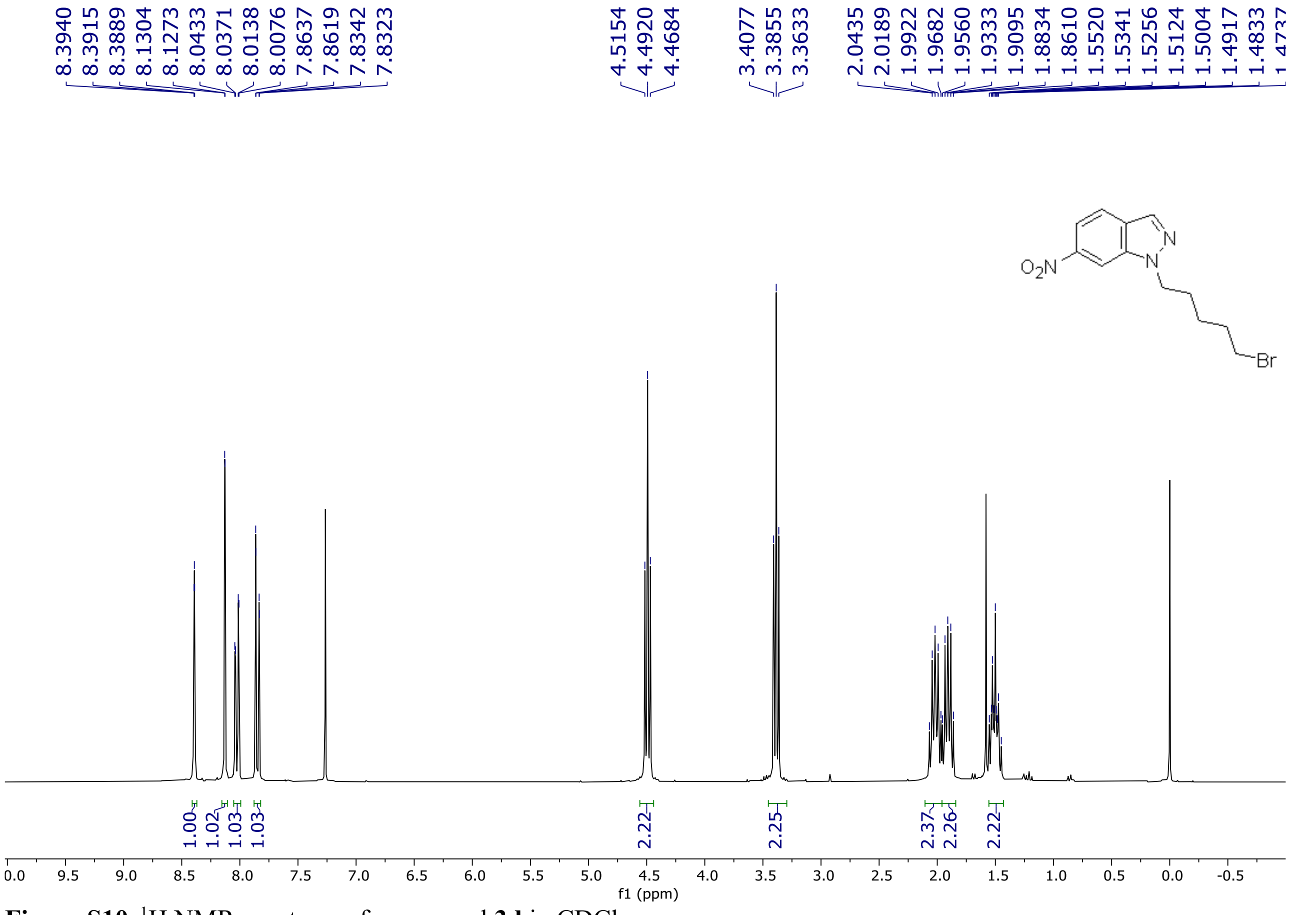

Figure S10. ^{1}H NMR spectrum of compound **3d** in $CDCl_3$.

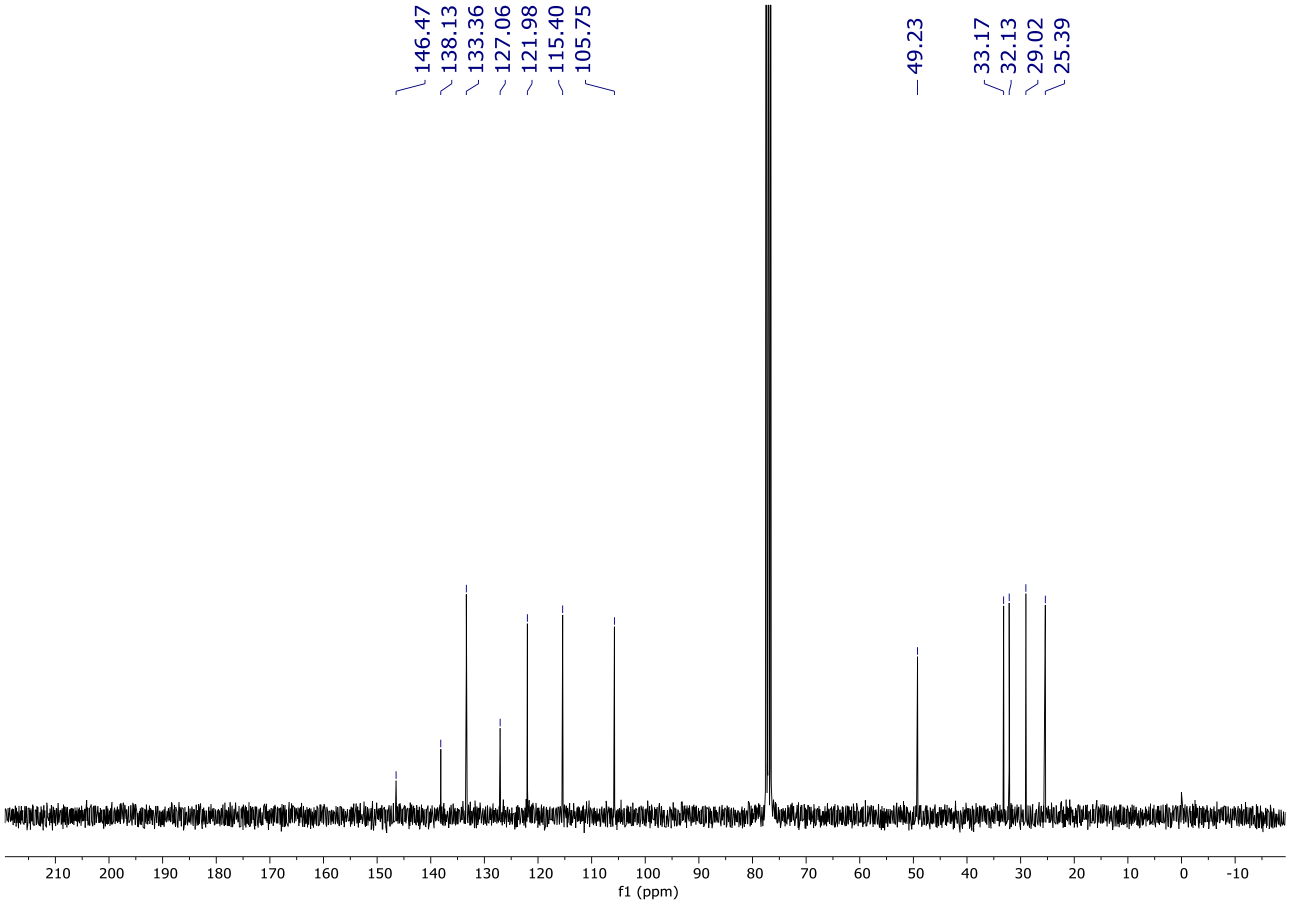

Figure S11. ^{13}C NMR spectrum of compound **3d** in $CDCl_3$.

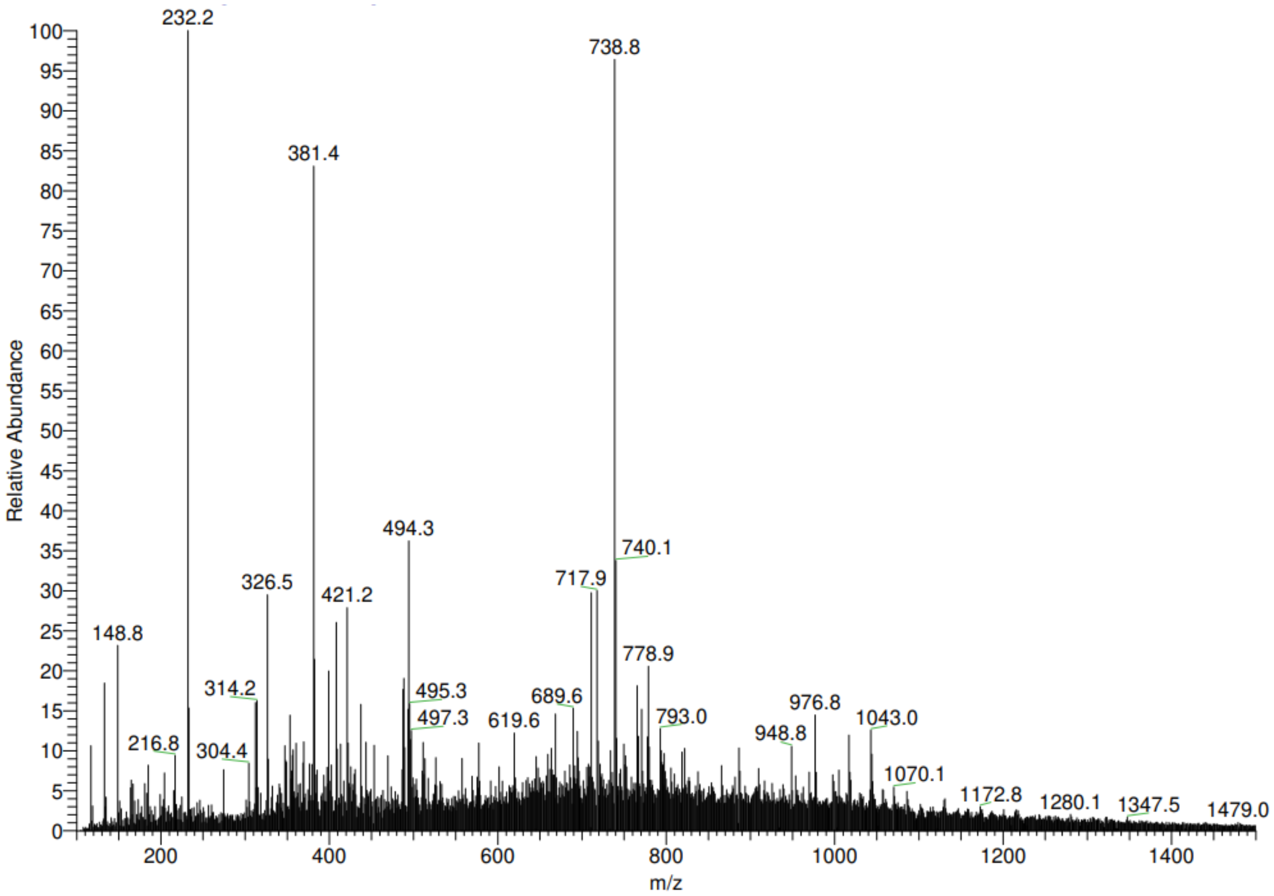

Figure S12. MS-ESI(+) spectrum of compound **3d**.

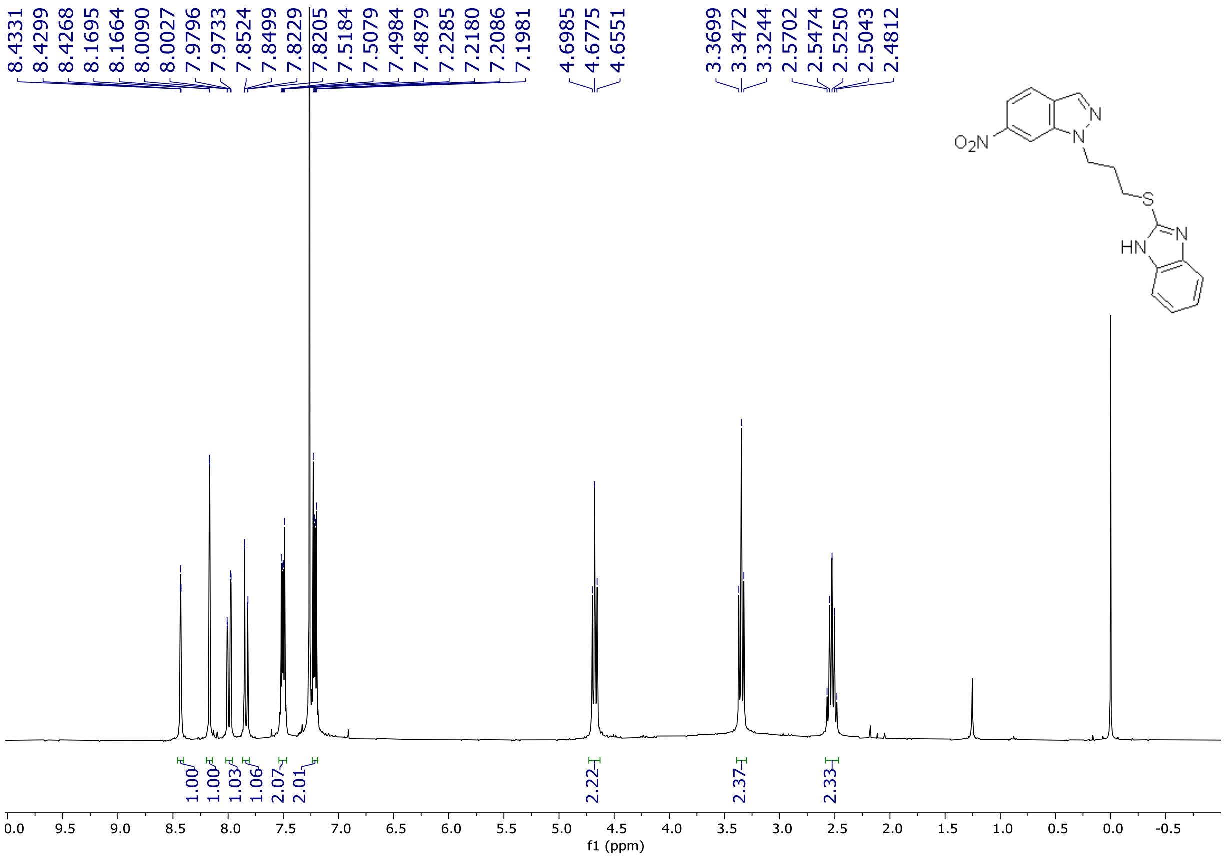

Figure S13. ^{1}H NMR spectrum of compound **M1** in $CDCl_3$.

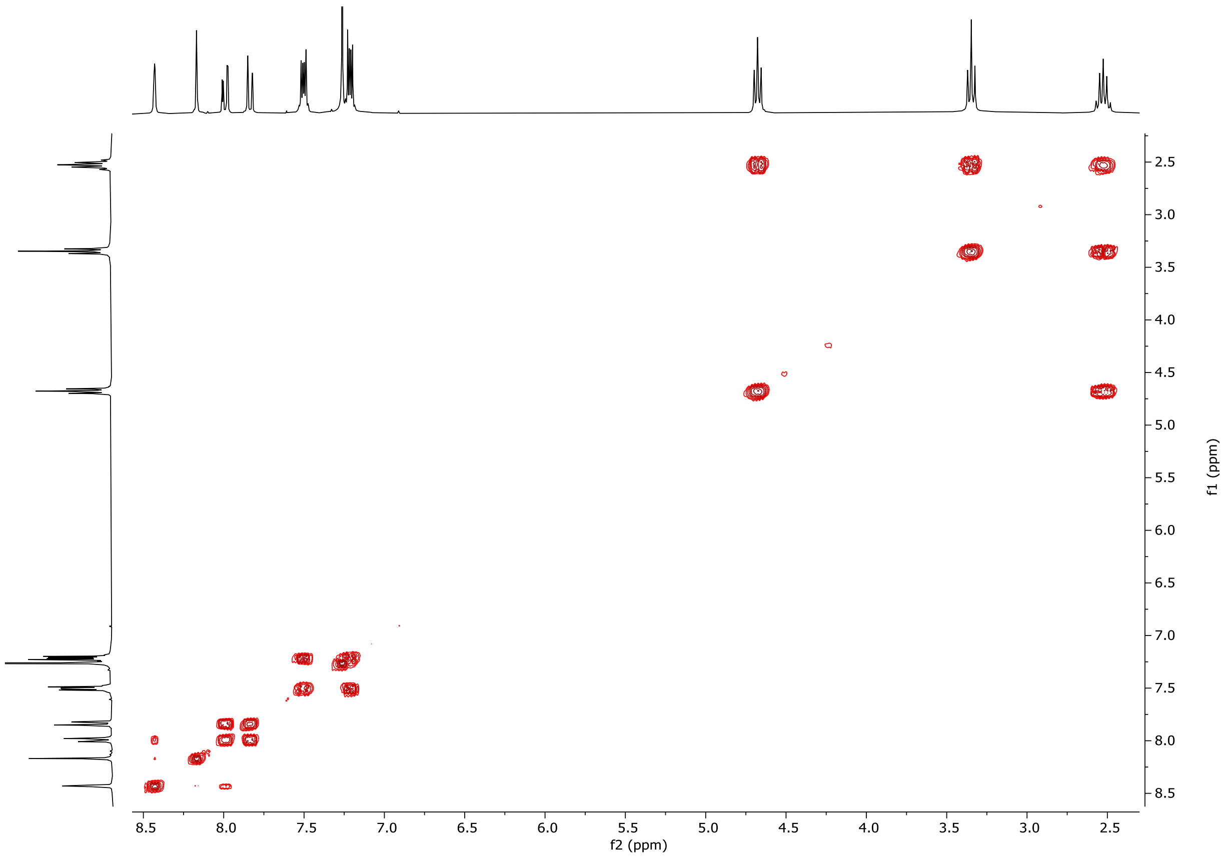

Figure S14. ^{1}H/^{1}H COSY NMR spectrum of compound **M1** in $CDCl_3$.

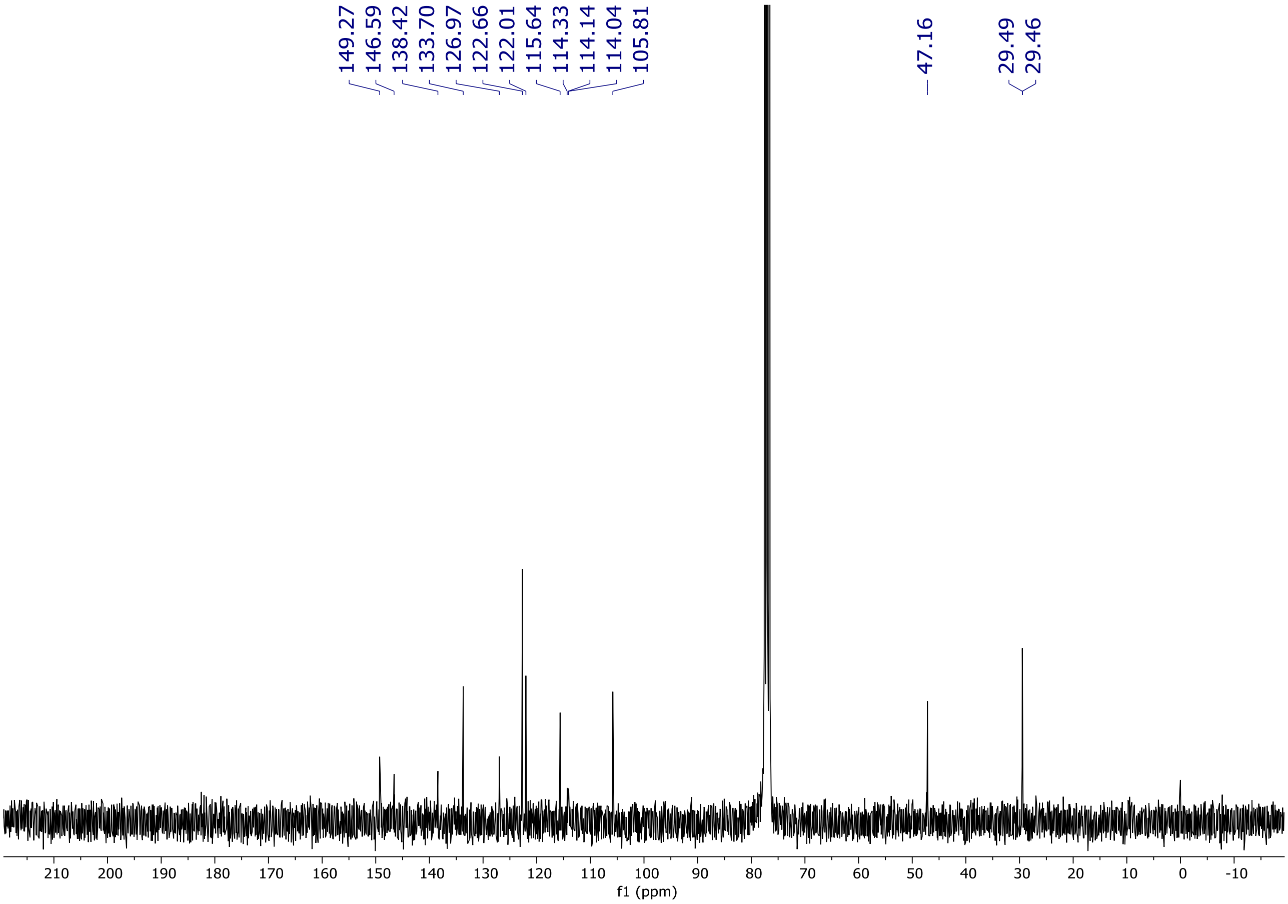

Figure S15. ^{13}C NMR spectrum of compound **M1** in $CDCl_3$.

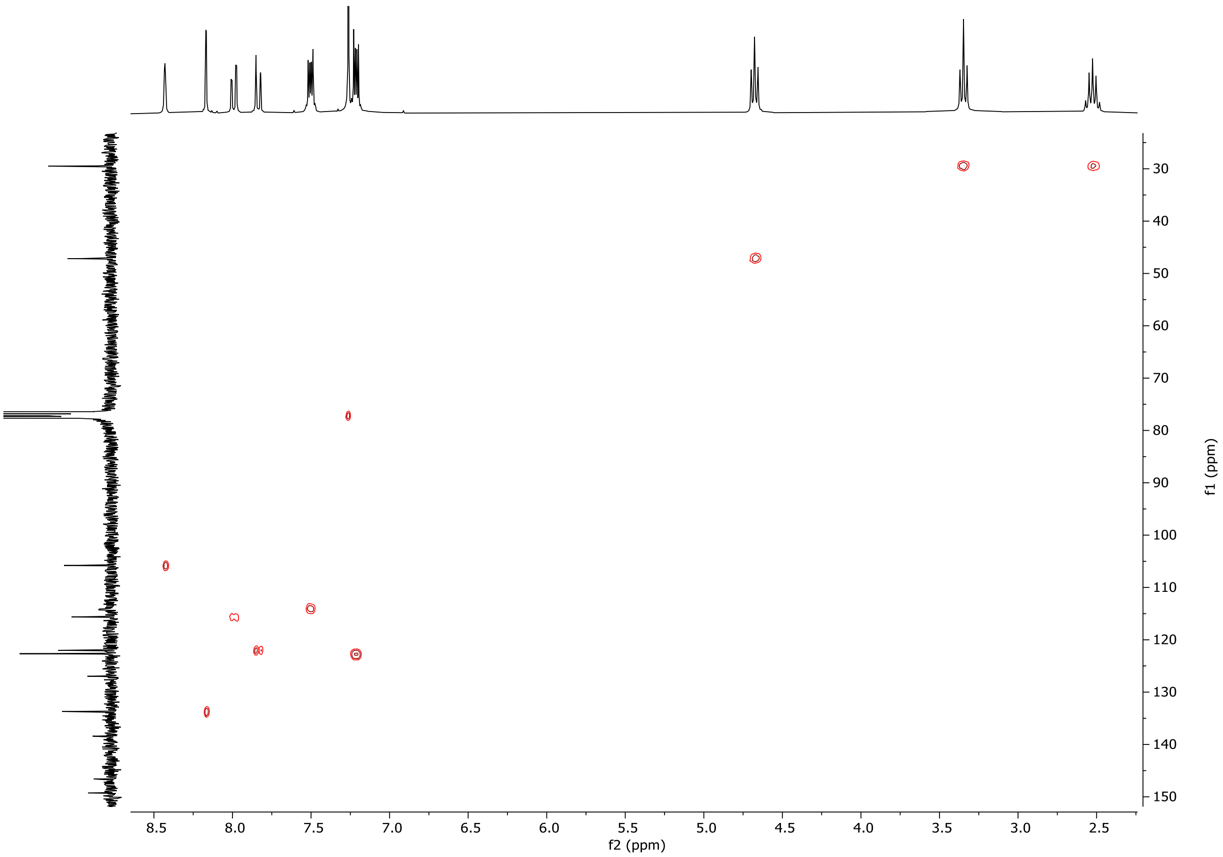

Figure S16. $^{1}H/^{13}C$ HSQC NMR spectrum of compound **M1** in $CDCl_3$.

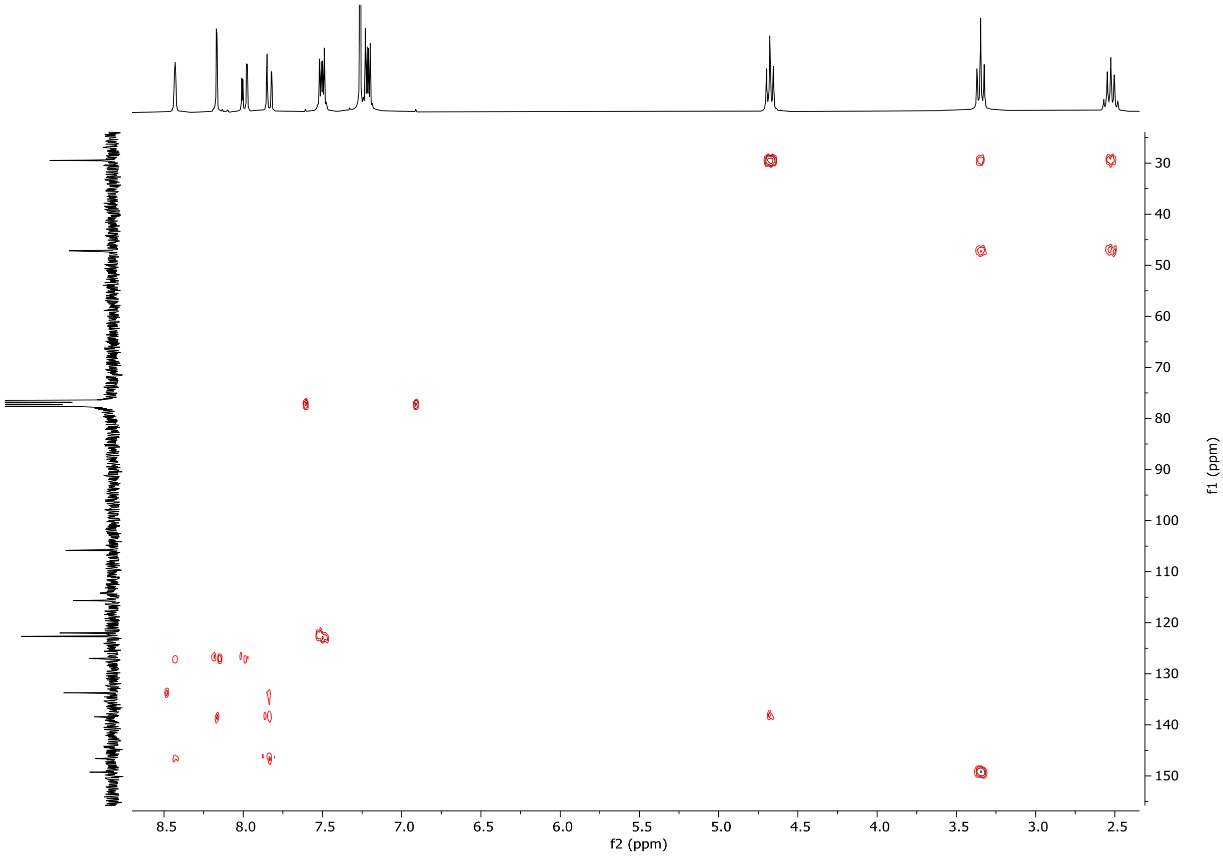

Figure S17. $^{1}H/^{13}C$ HMBC NMR spectrum of compound **M1** in $CDCl_3$.

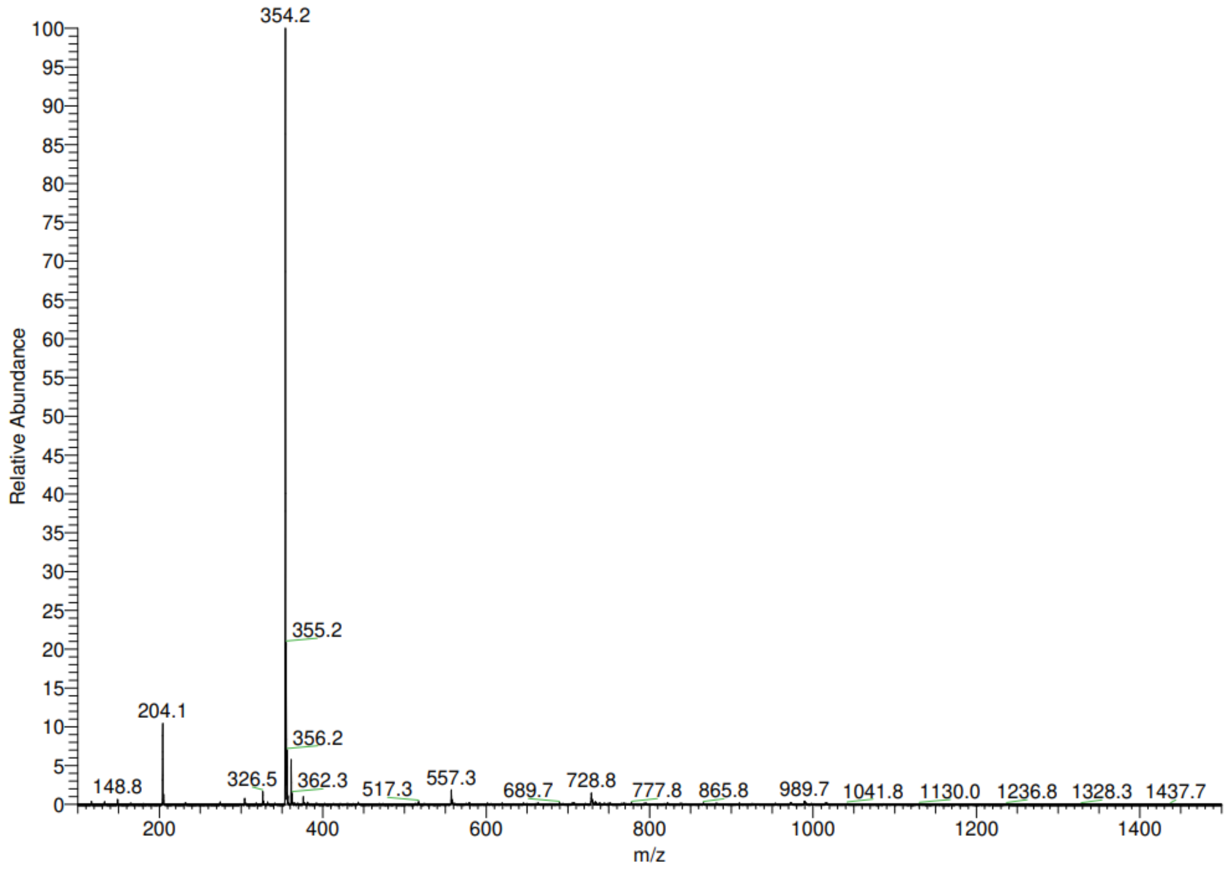

Figure S18. MS-ESI(+) spectrum of compound **M1**.

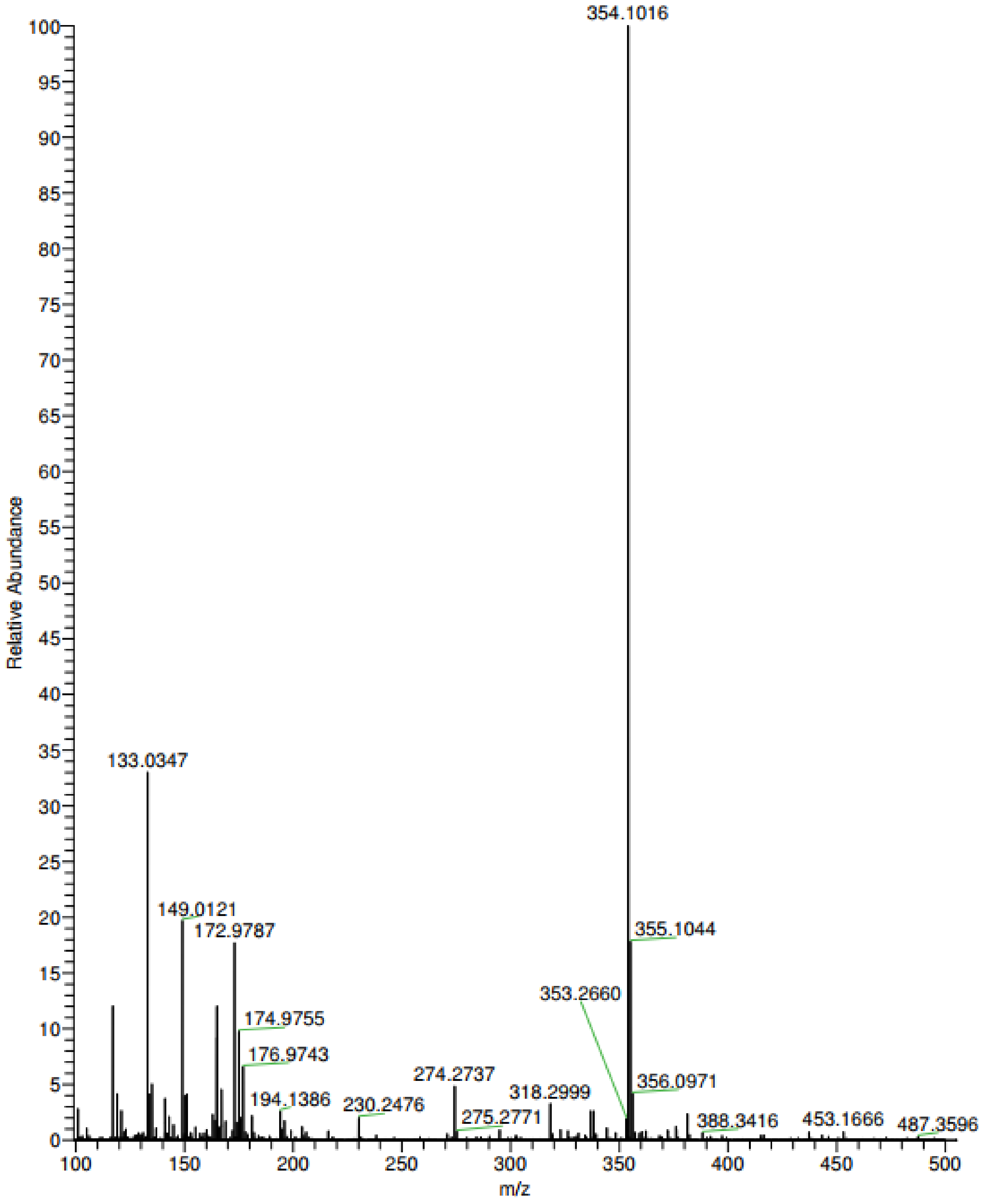

Figure S19. HRMS-ESI(+) spectrum of compound **M1**.

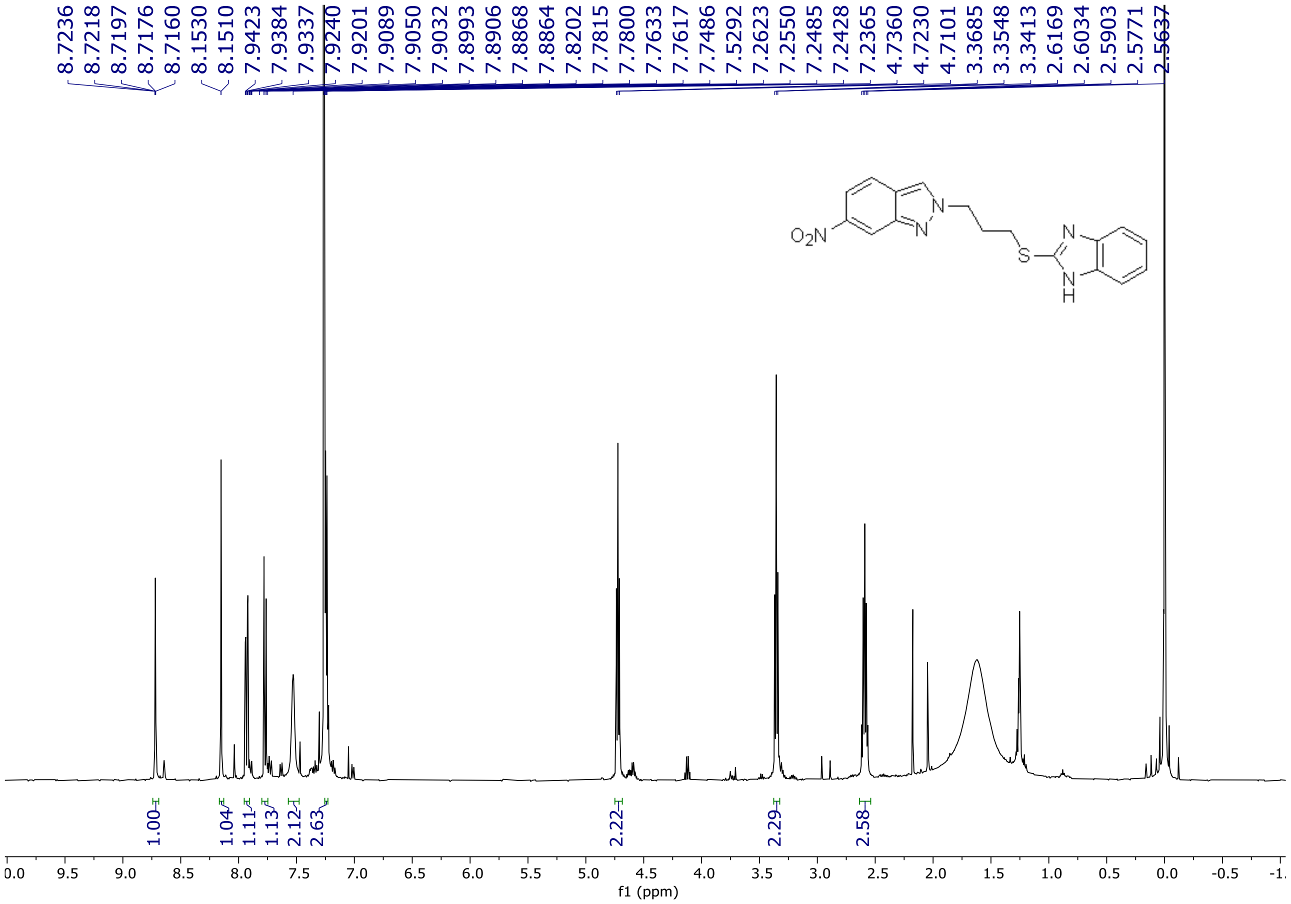

Figure S20. ^{1}H NMR spectrum of compound **M2** in $CDCl_3$.

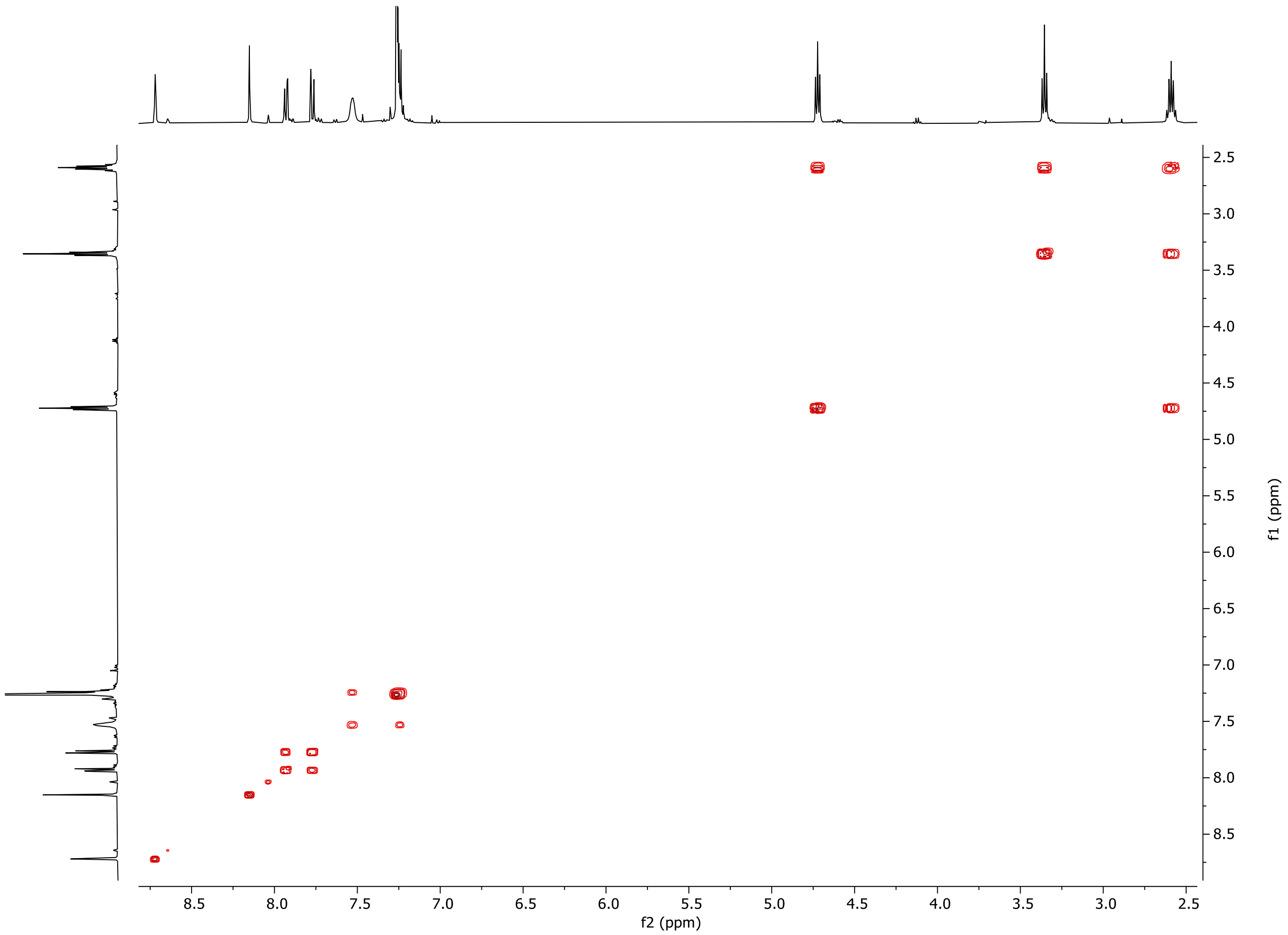

Figure S21. $^{1}H/^{1}H$ COSY NMR spectrum of compound **M2** in $CDCl_3$.

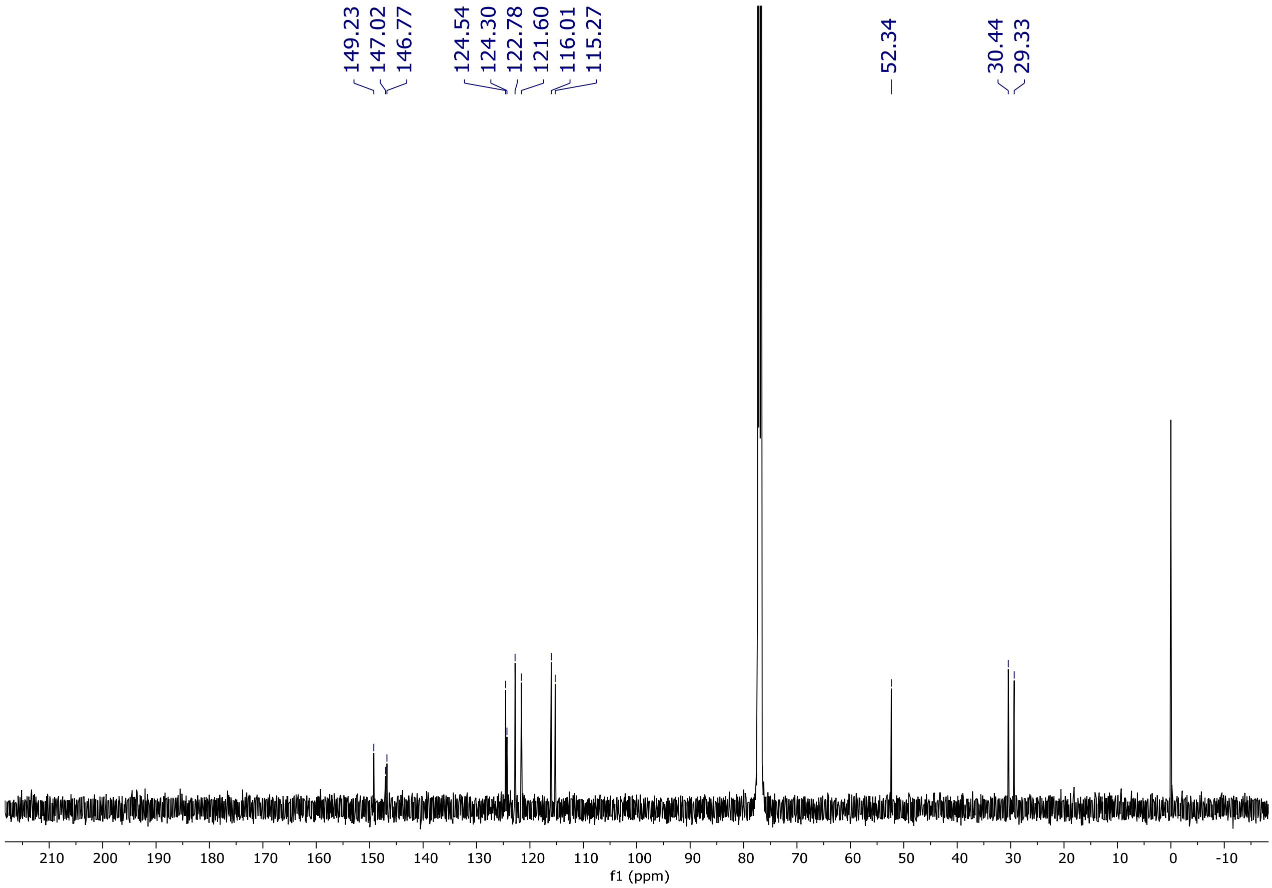

Figure S22. ^{13}C NMR spectrum of compound **M2** in $CDCl_3$.

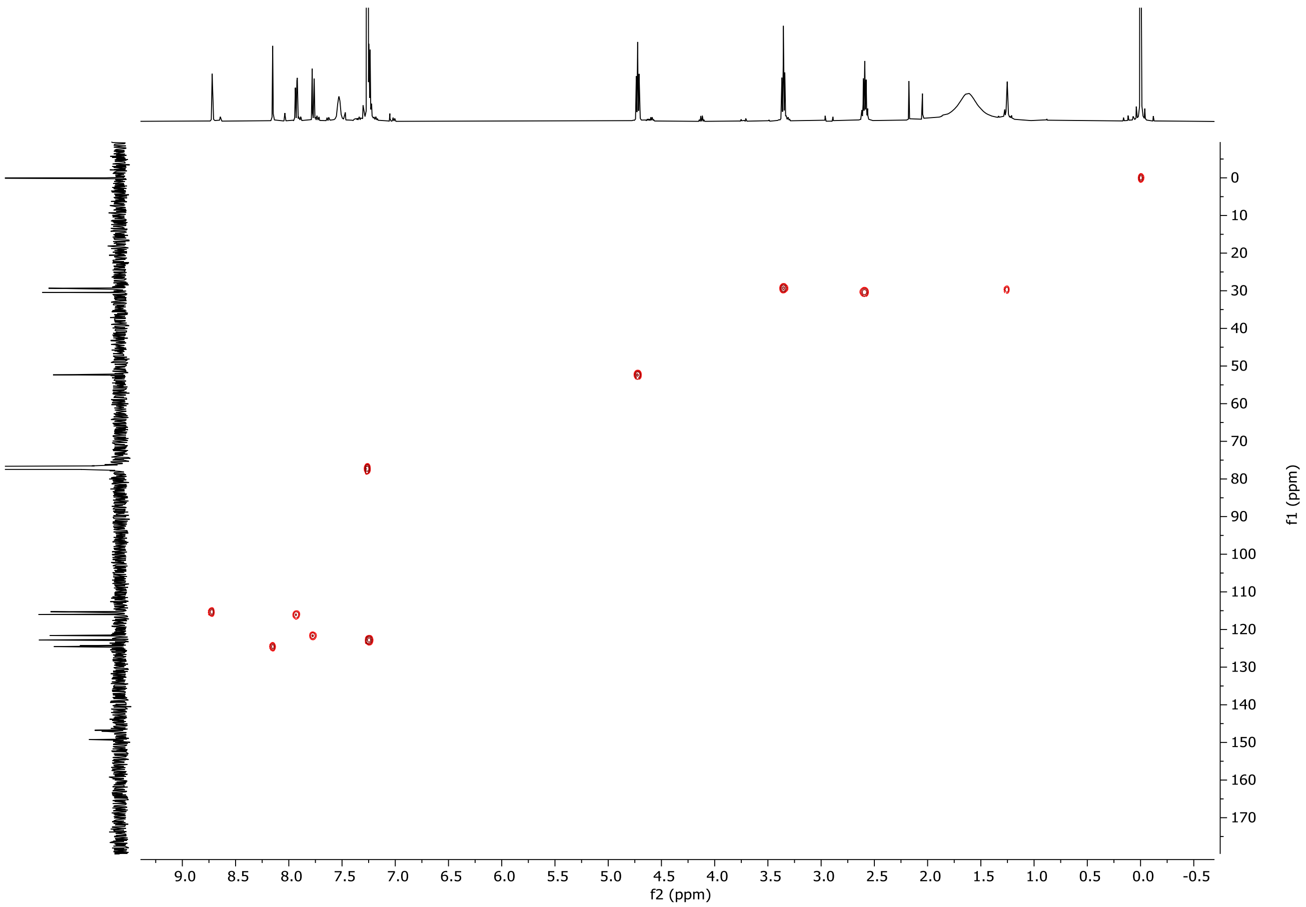

Figure S23. $^{1}H/^{13}C$ HSQC NMR spectrum of compound **M2** in $CDCl_3$.

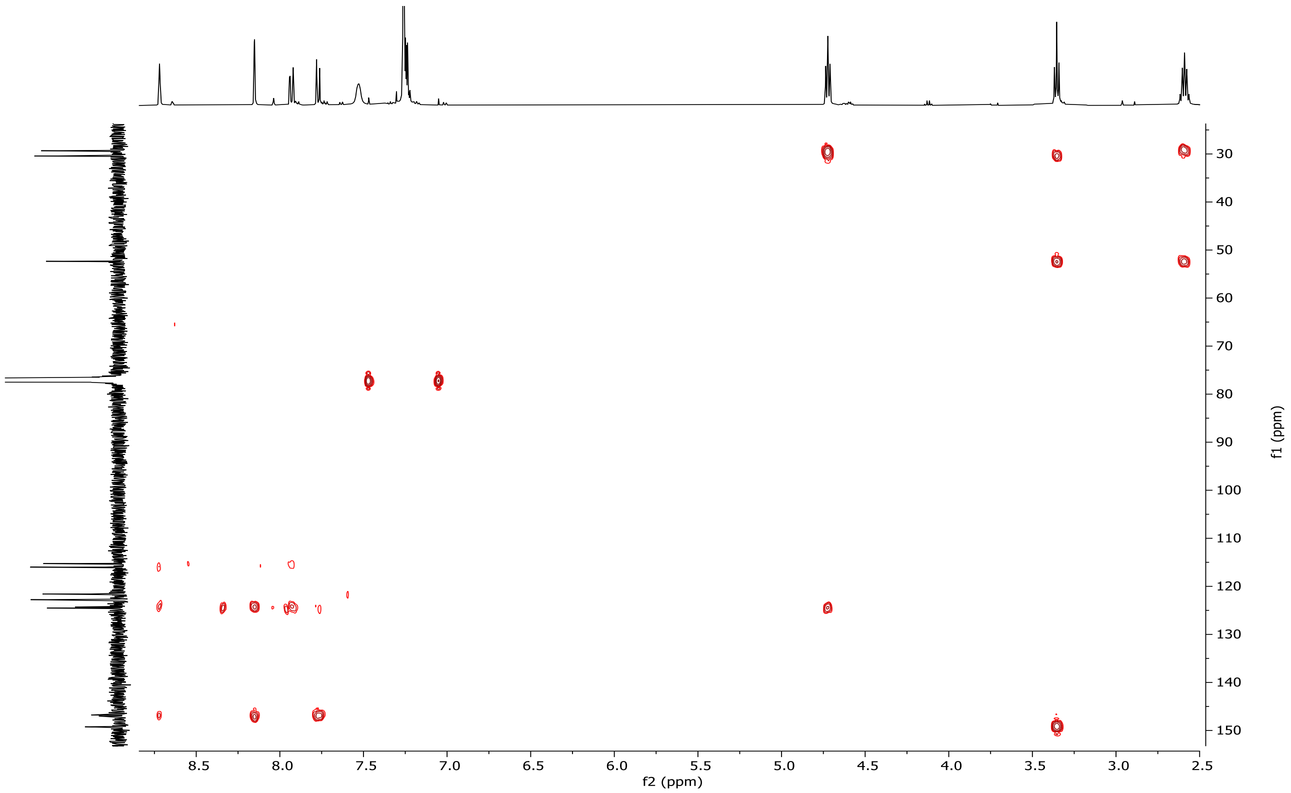

Figure S24. $^{1}H/^{13}C$ HMBC NMR spectrum of compound **M2** in $CDCl_3$.

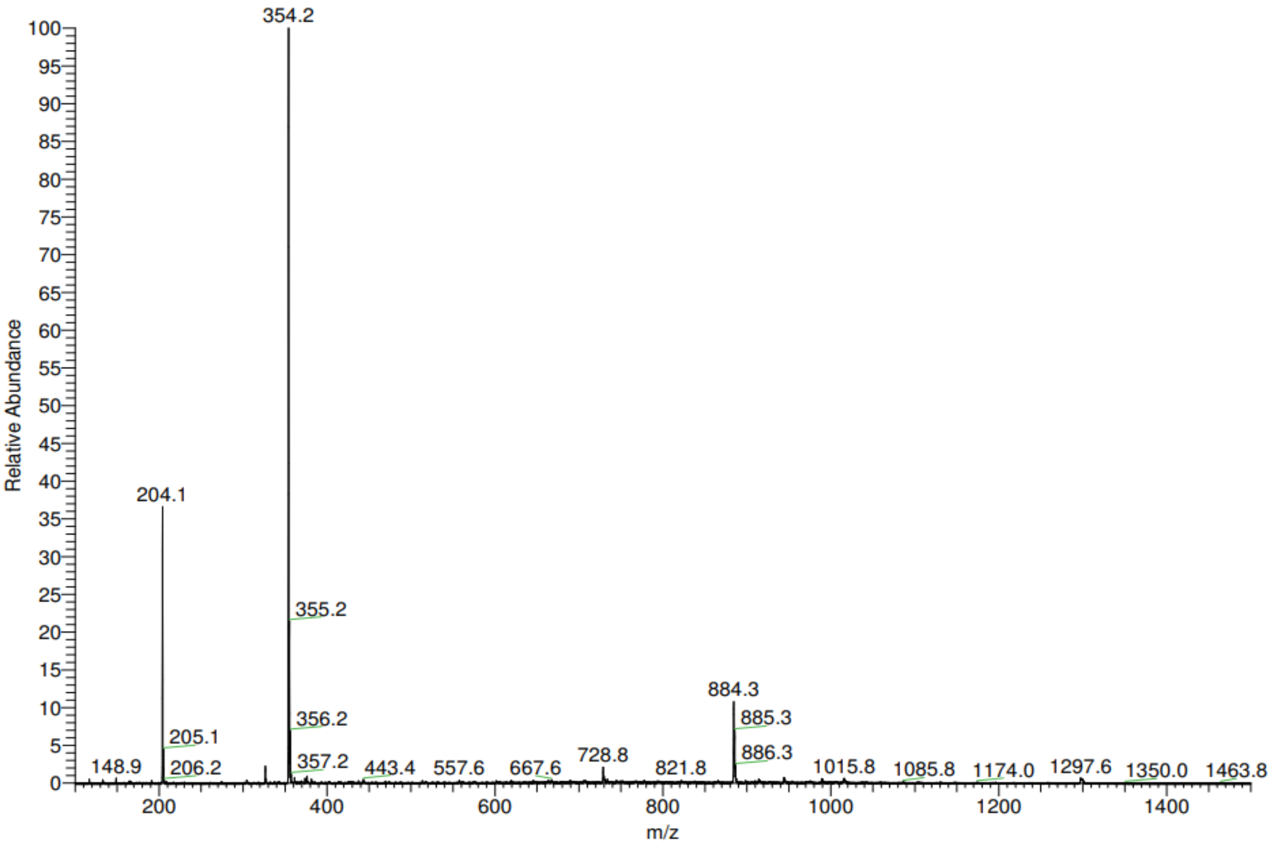

Figure S25. MS-ESI(+) spectrum of compound **M2**.

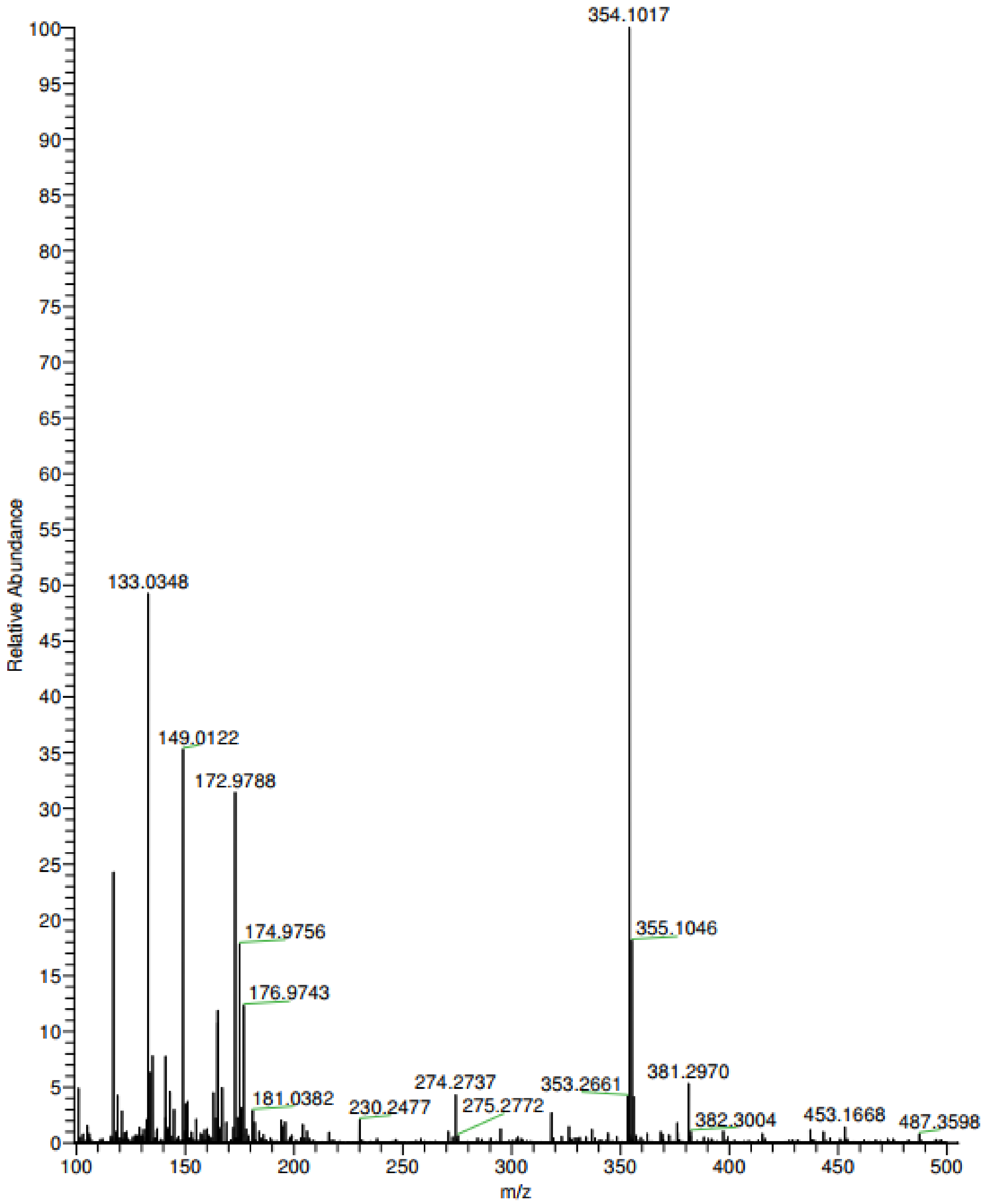

Figure S26. HRMS-ESI(+) spectrum of compound **M2**.

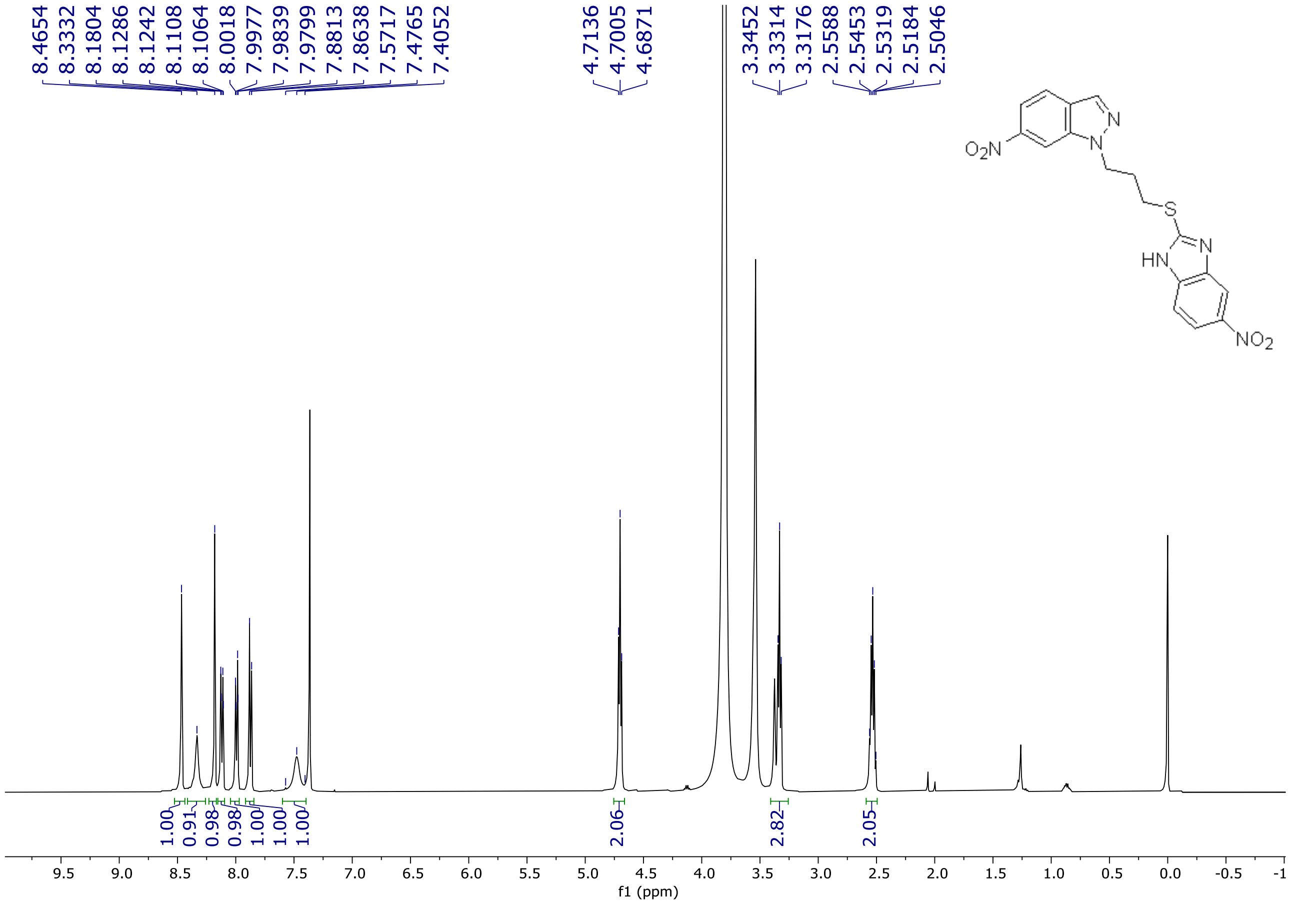

Figure S27. ^{1}H NMR spectrum of compound **M3** in $CDCl_3/CD_3OD$.

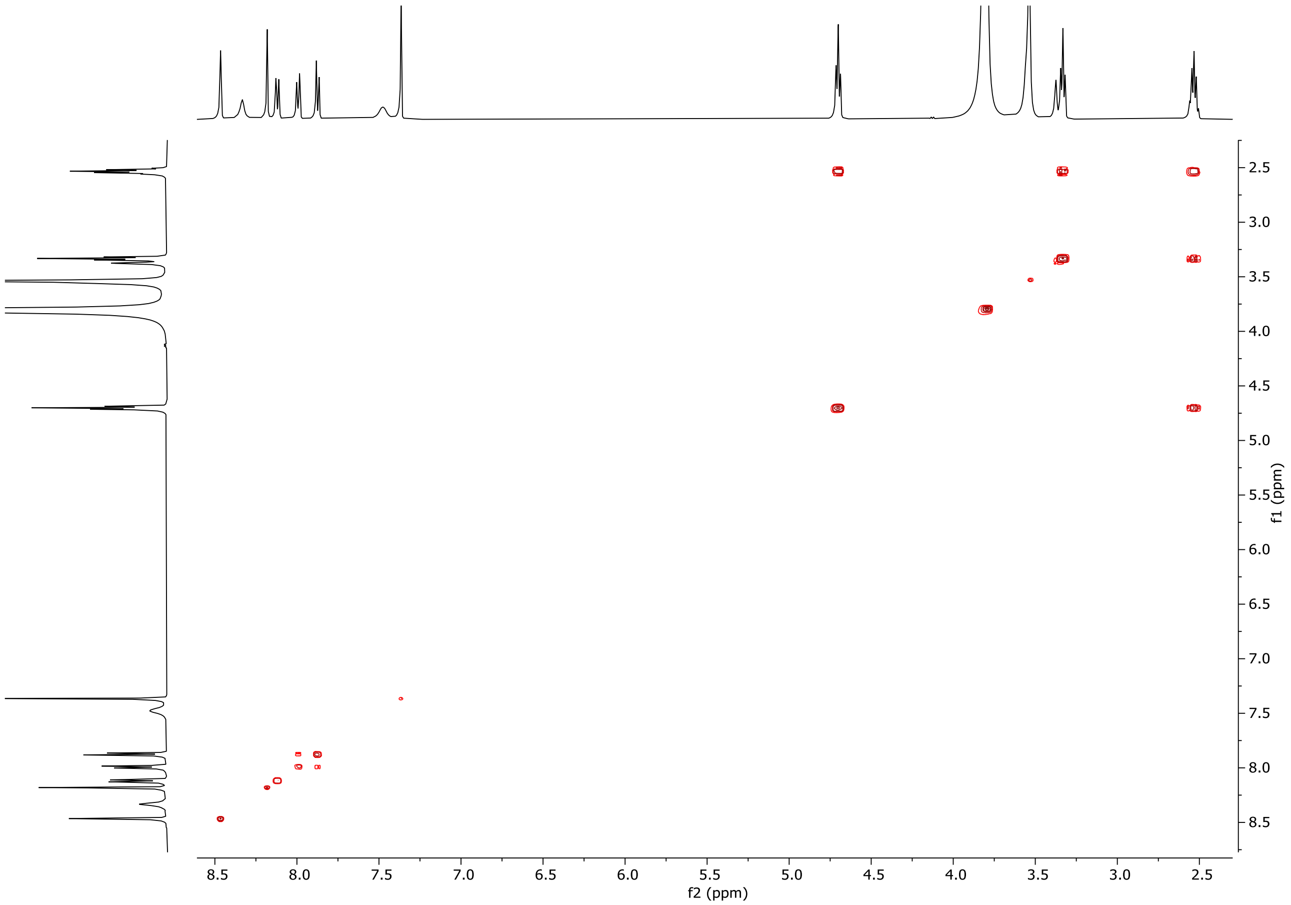

Figure S28. $^{1}H/^{1}H$ COSY NMR spectrum of compound **M3** in $CDCl_3/CD_3OD$.

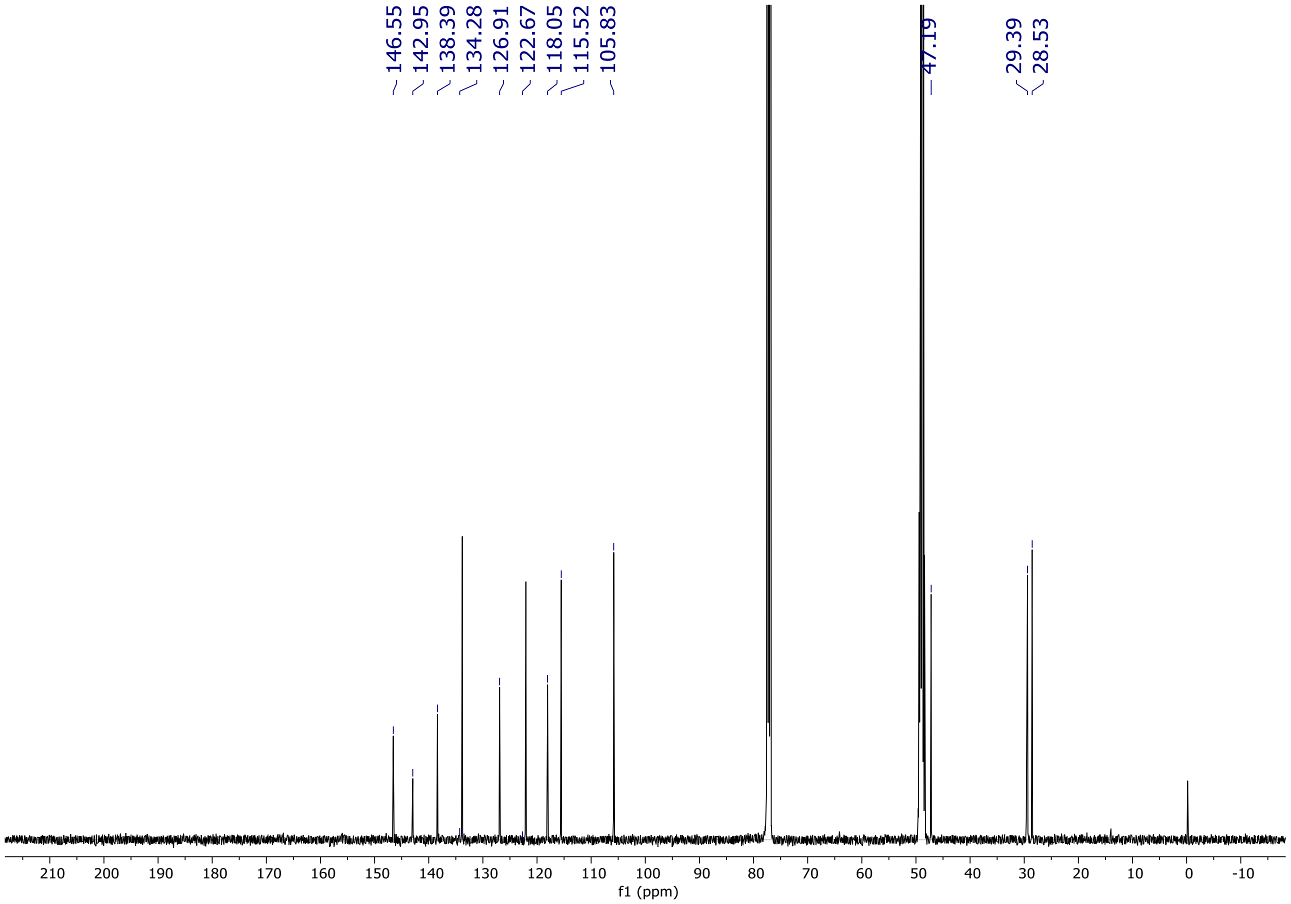

Figure S29. ^{13}C NMR spectrum of compound **M3** in $CDCl_3/CD_3OD$.

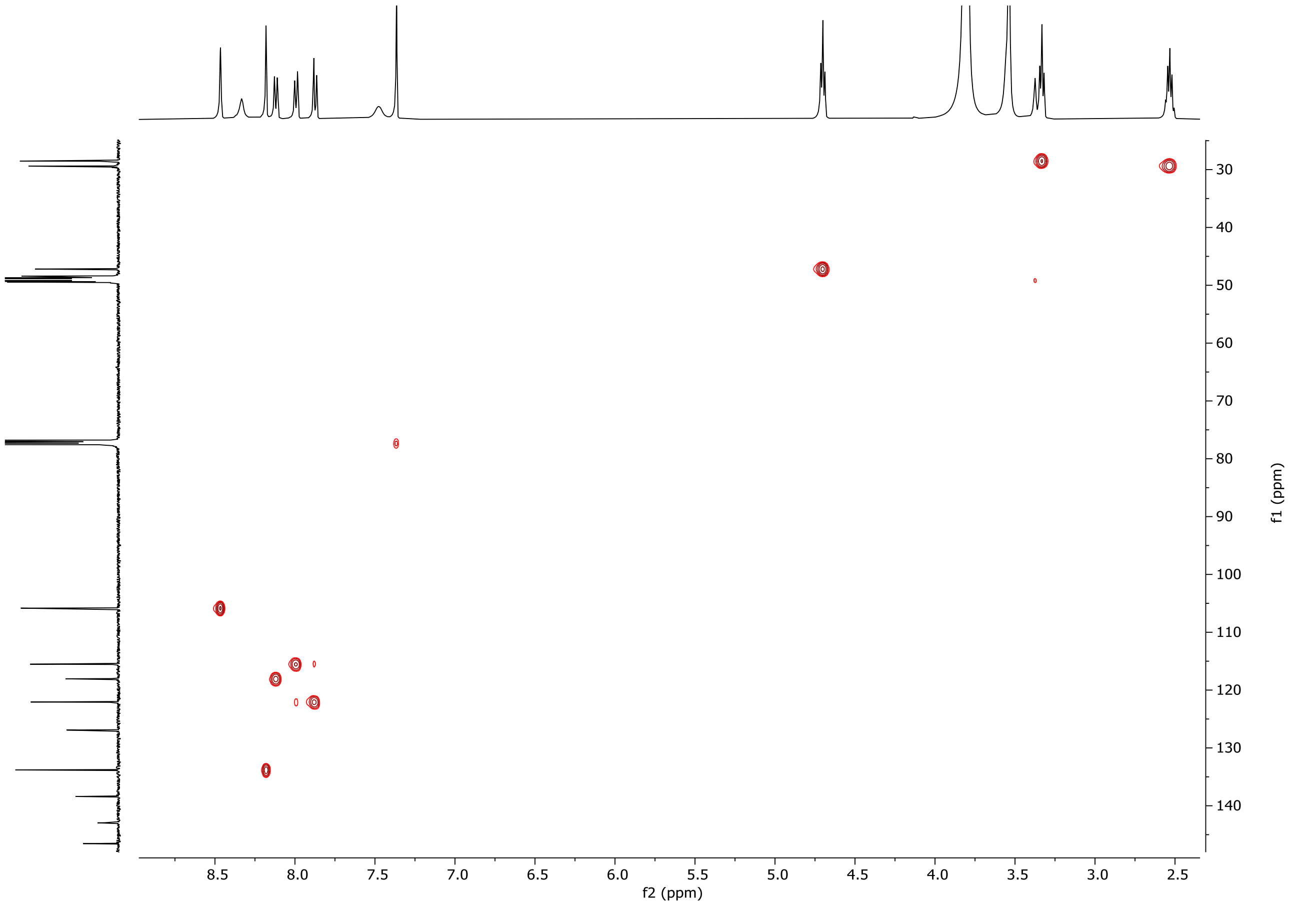

Figure S30. $^{1}H/^{13}C$ HSQC NMR spectrum of compound **M3** in $CDCl_3/CD_3OD$.

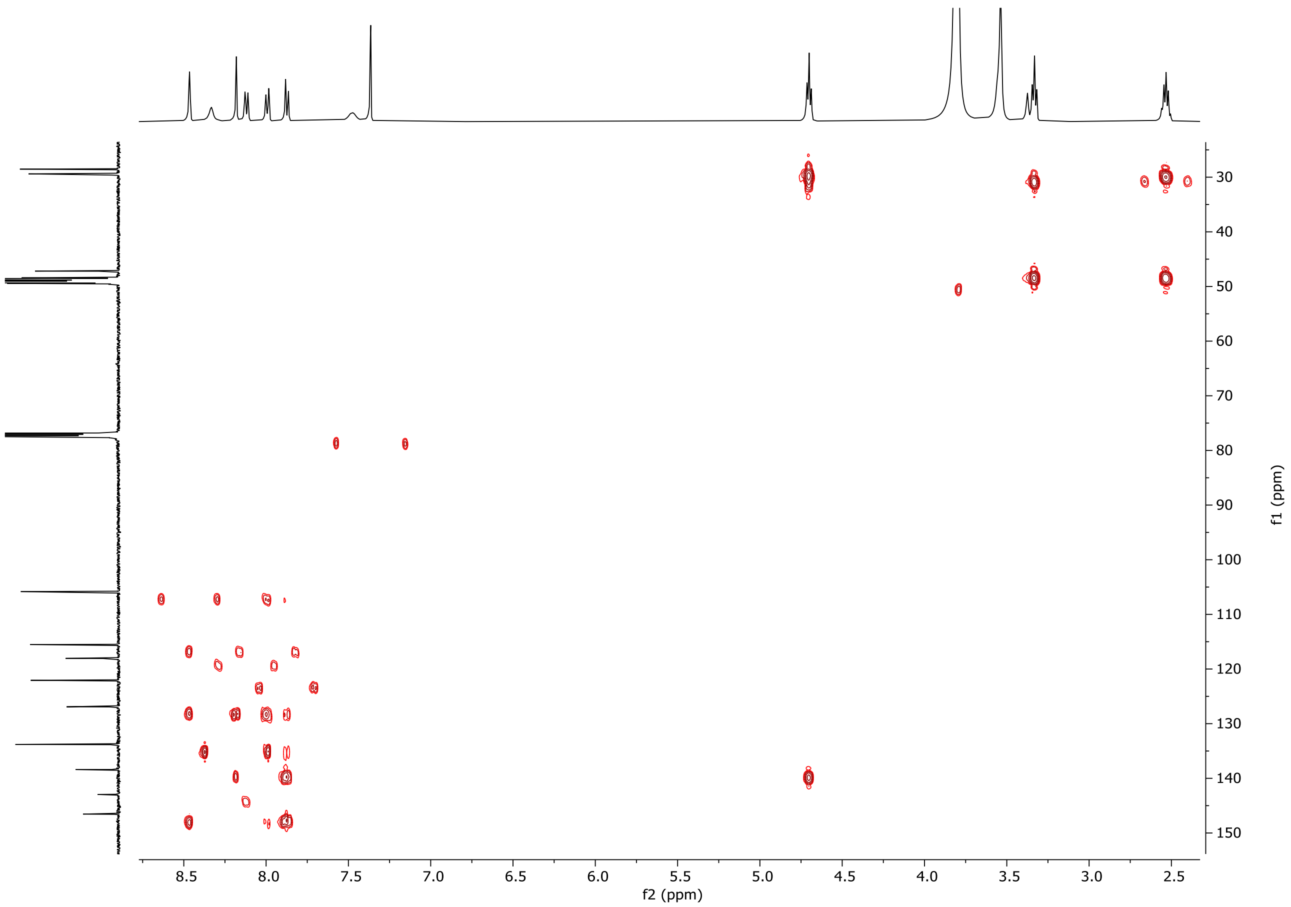

Figure S31. $^{1}H/^{13}C$ HMBC NMR spectrum of compound **M3** in $CDCl_3/CD_3OD$.

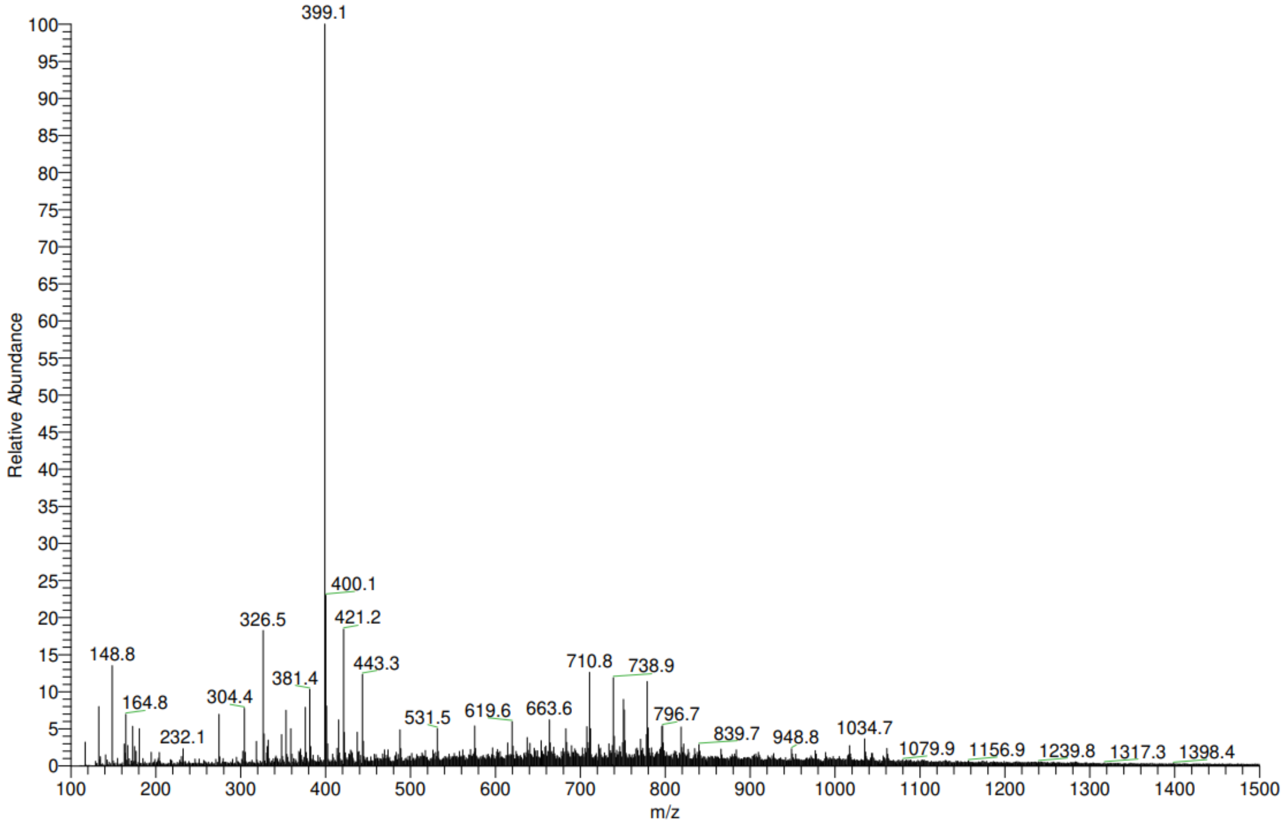

Figure S32. MS-ESI(+) spectrum of compound **M3**.

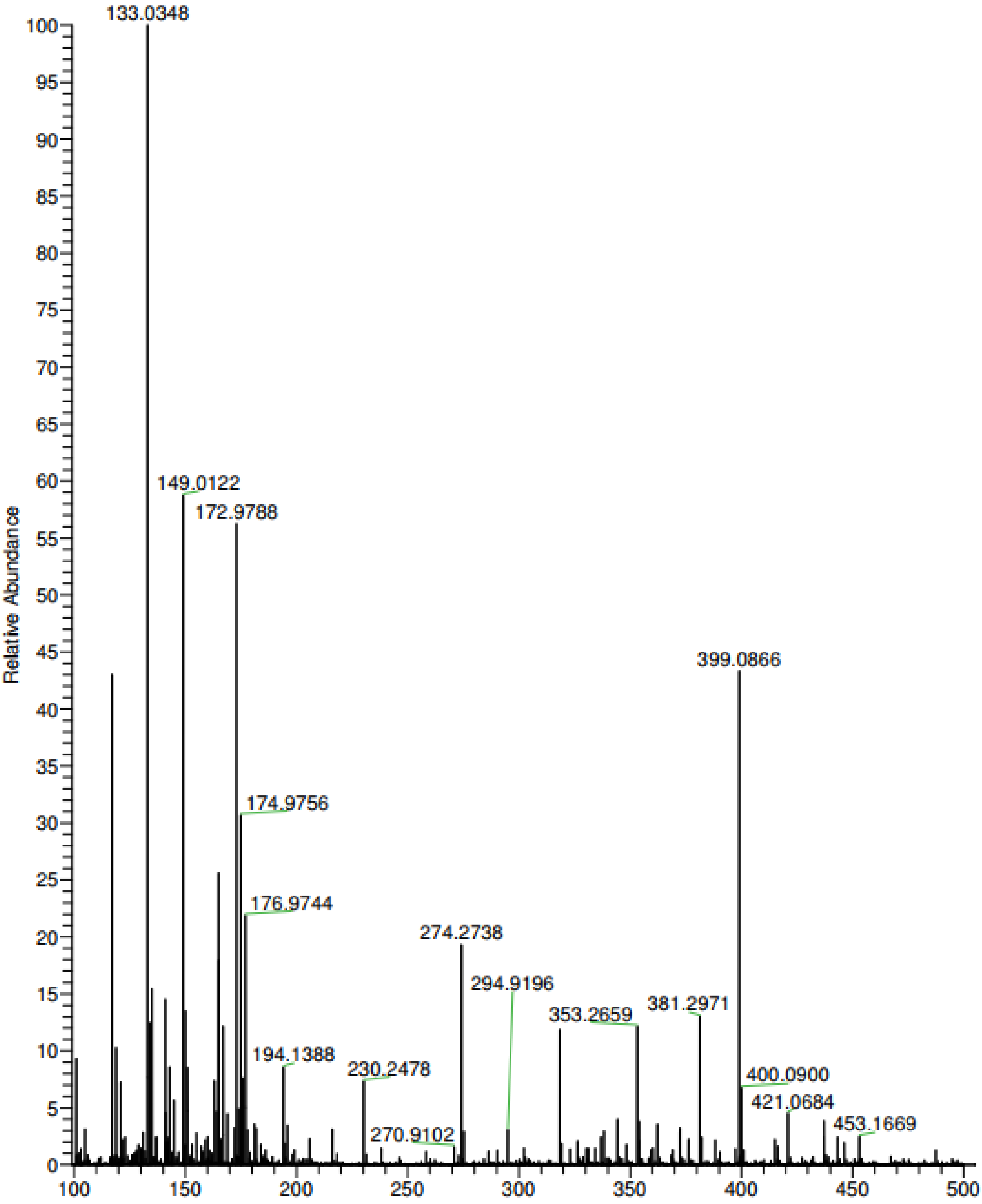

Figure S33. HRMS-ESI(+) spectrum of compound **M3**.

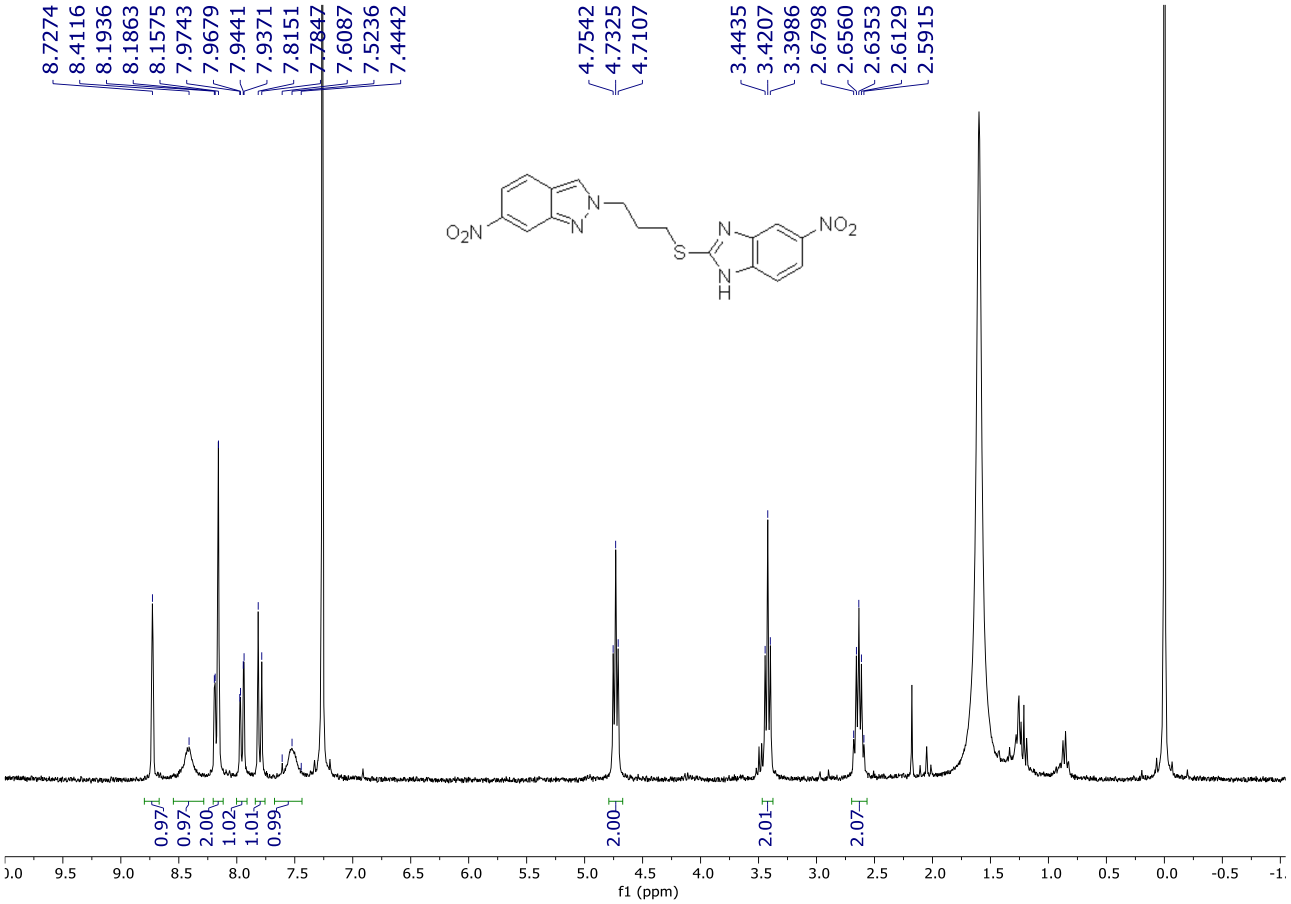

Figure S34. ^{1}H NMR spectrum of compound **M4** in $CDCl_3$.

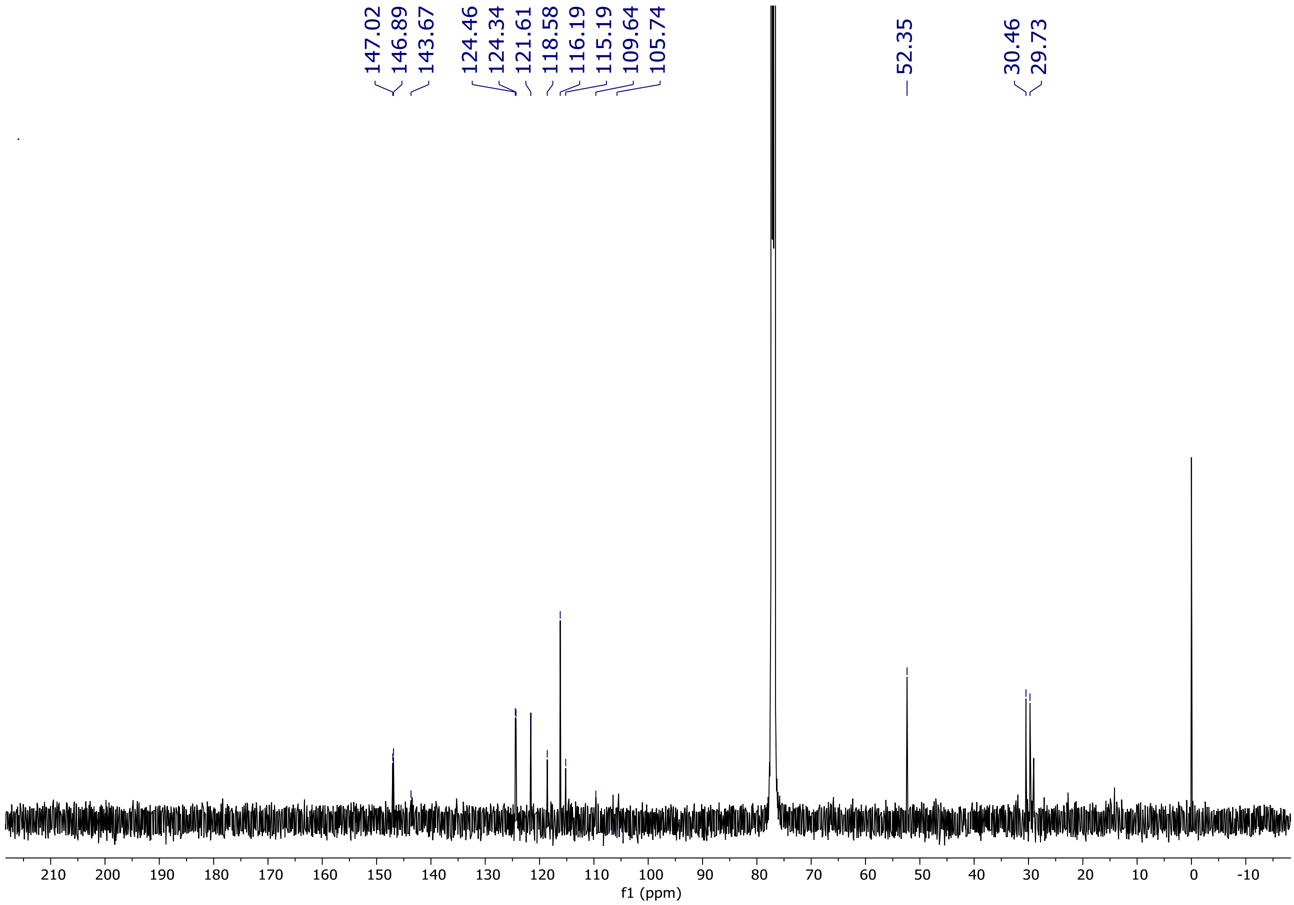

Figure S35. ^{13}C NMR spectrum of compound **M4** in $CDCl_3$.

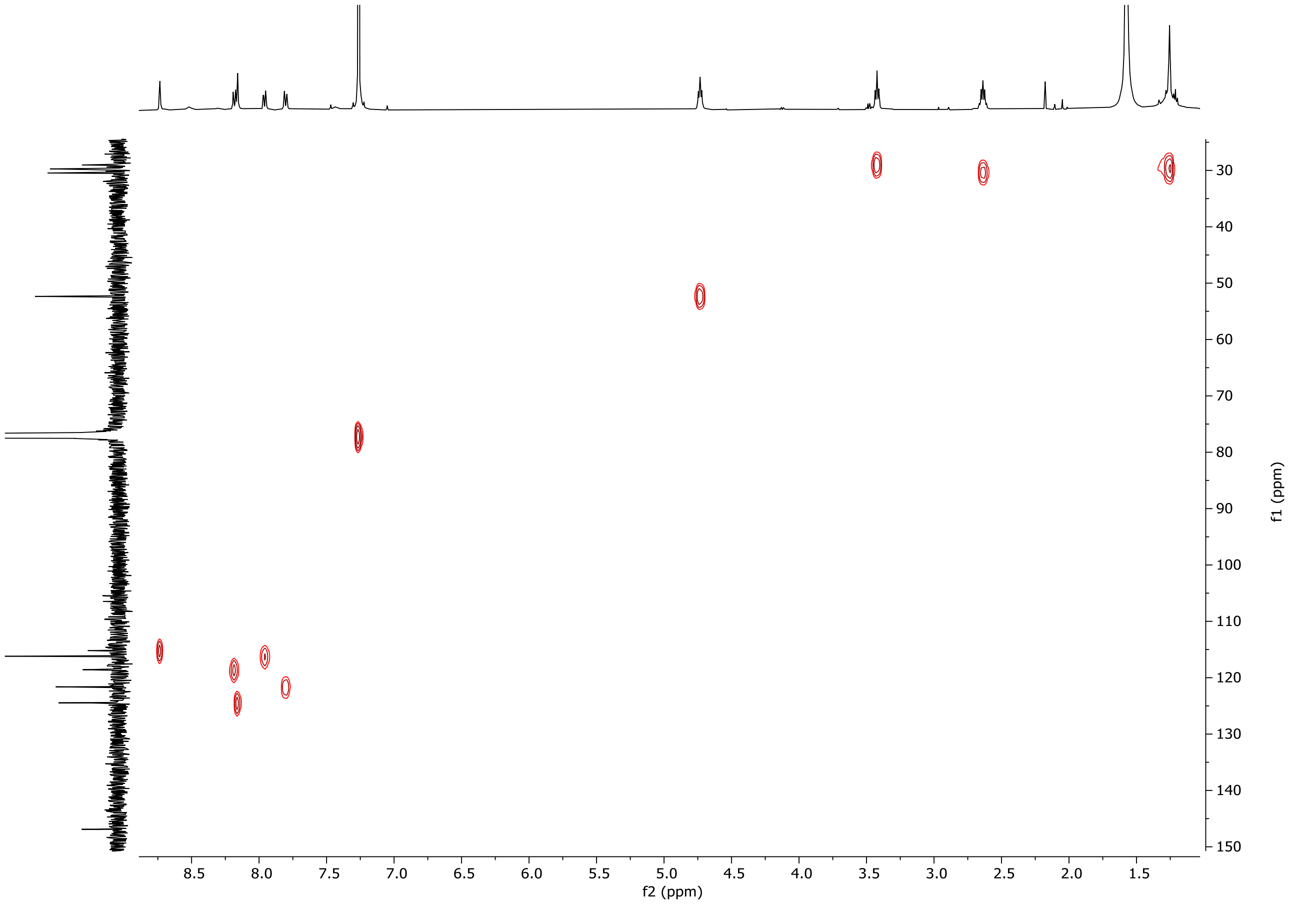

Figure S36. $^{1}H/^{13}C$ HSQC NMR spectrum of compound **M4** in $CDCl_3$.

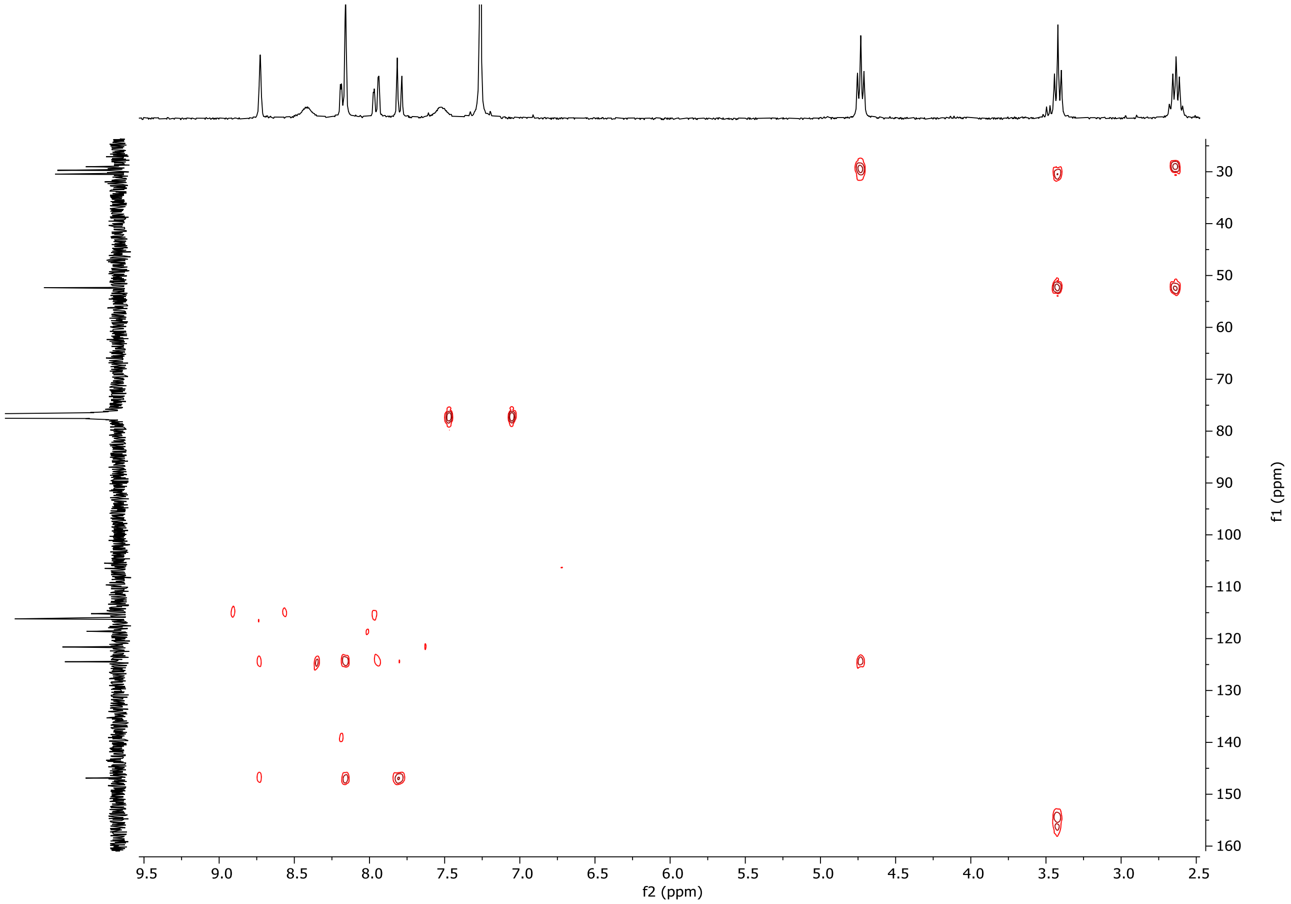

Figure S37. $^{1}H/^{13}C$ HMBC NMR spectrum of compound **M4** in $CDCl_3$.

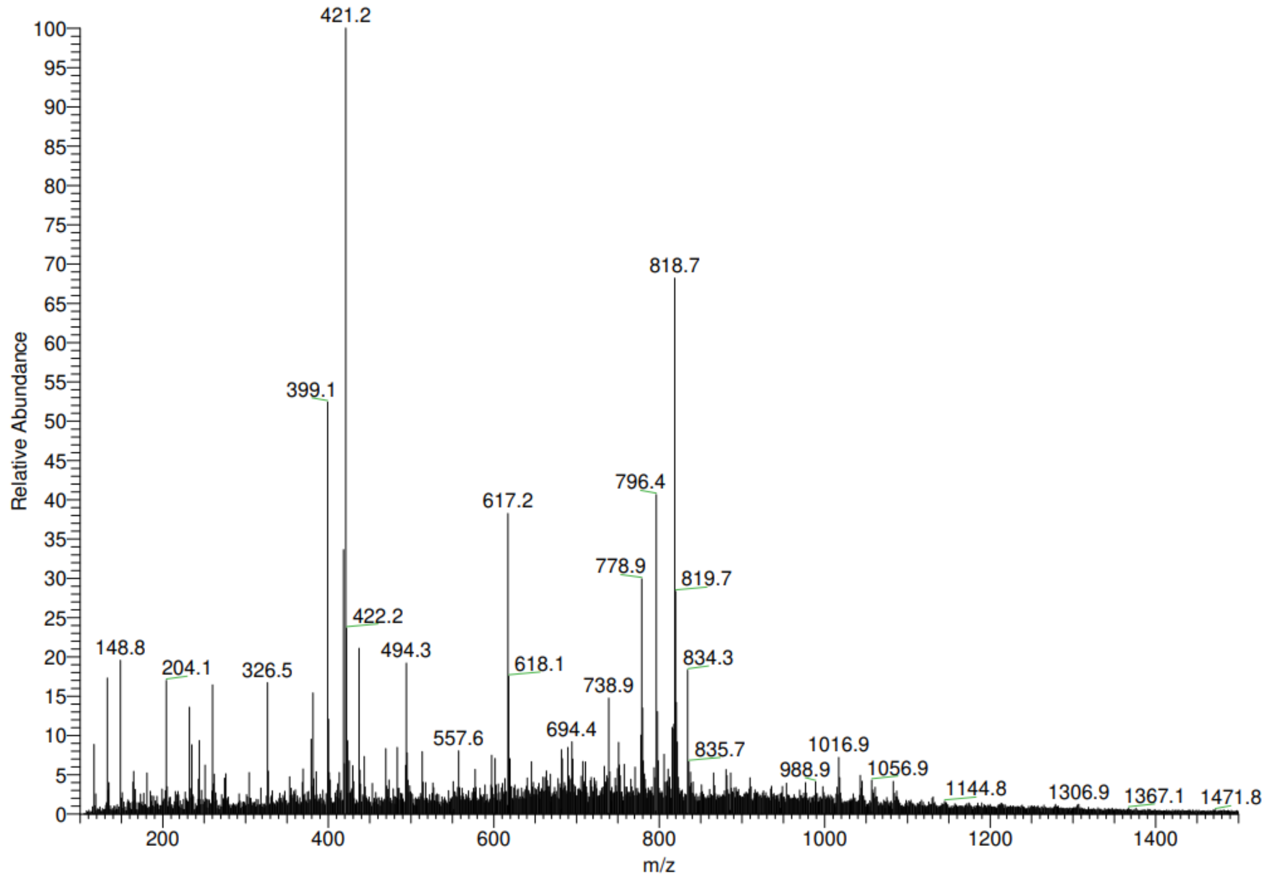

Figure S38. MS-ESI(+) spectrum of compound **M4**.

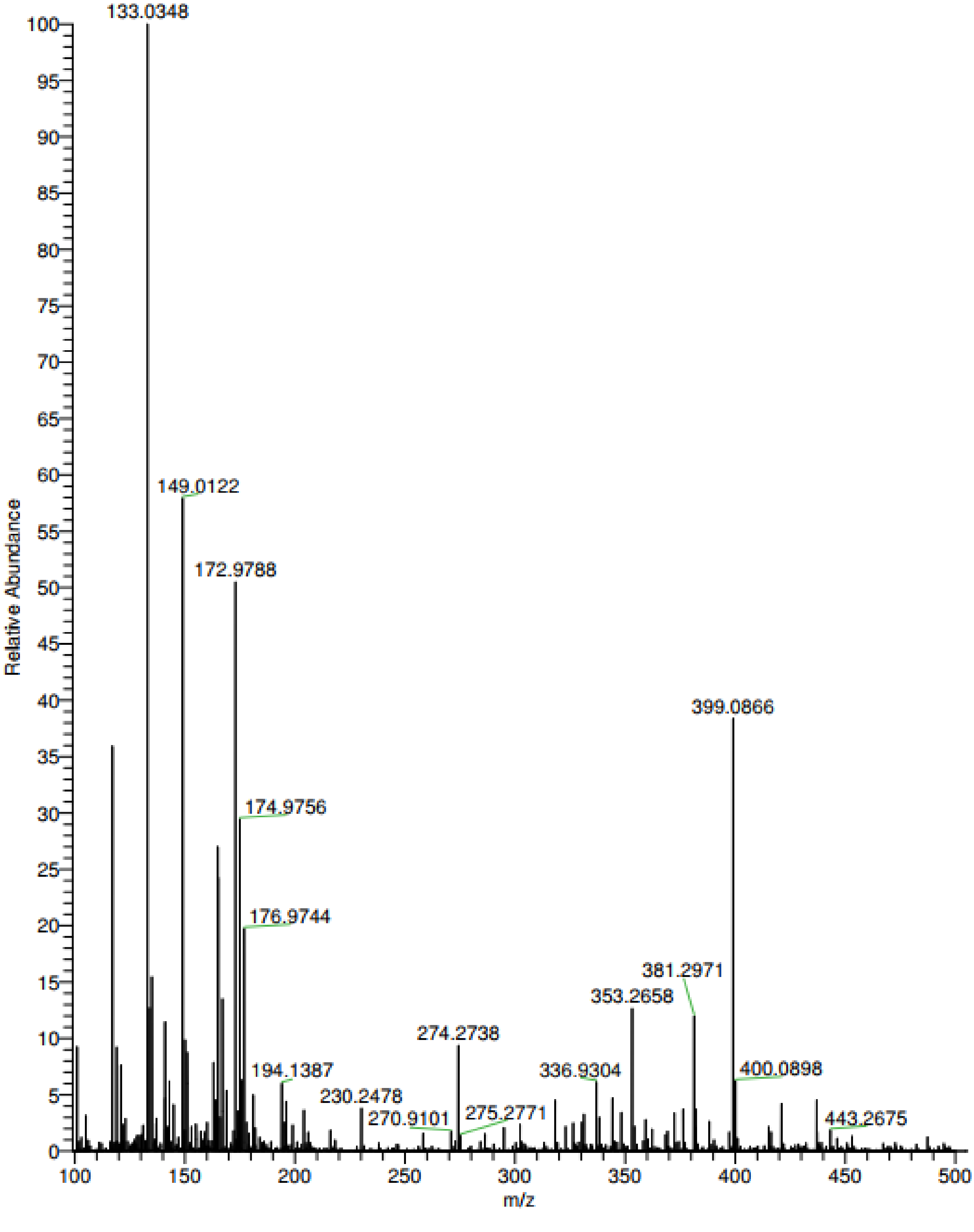

Figure S39. HRMS-ESI(+) spectrum of compound **M4**.

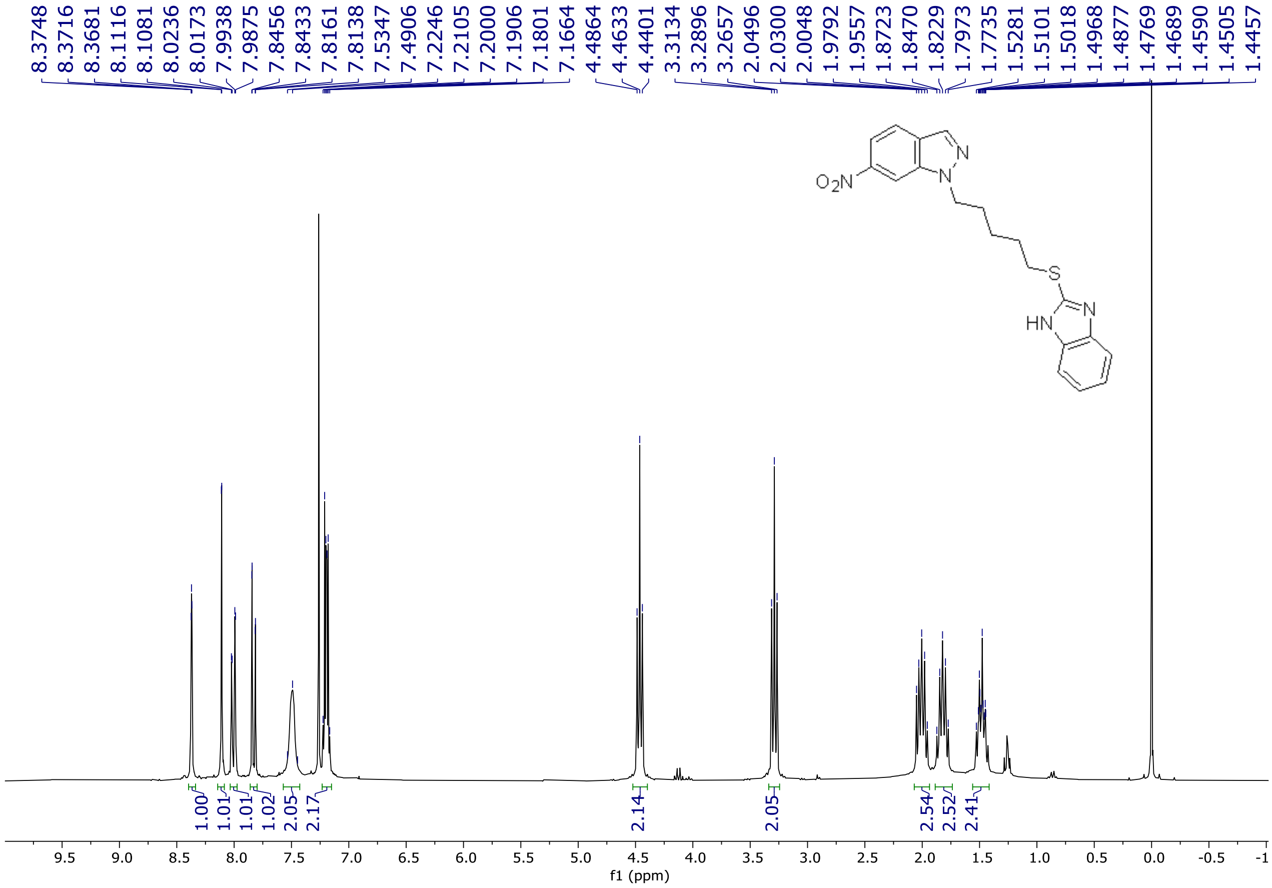

Figure S40. ^{1}H NMR spectrum of compound **M5** in $CDCl_3$.

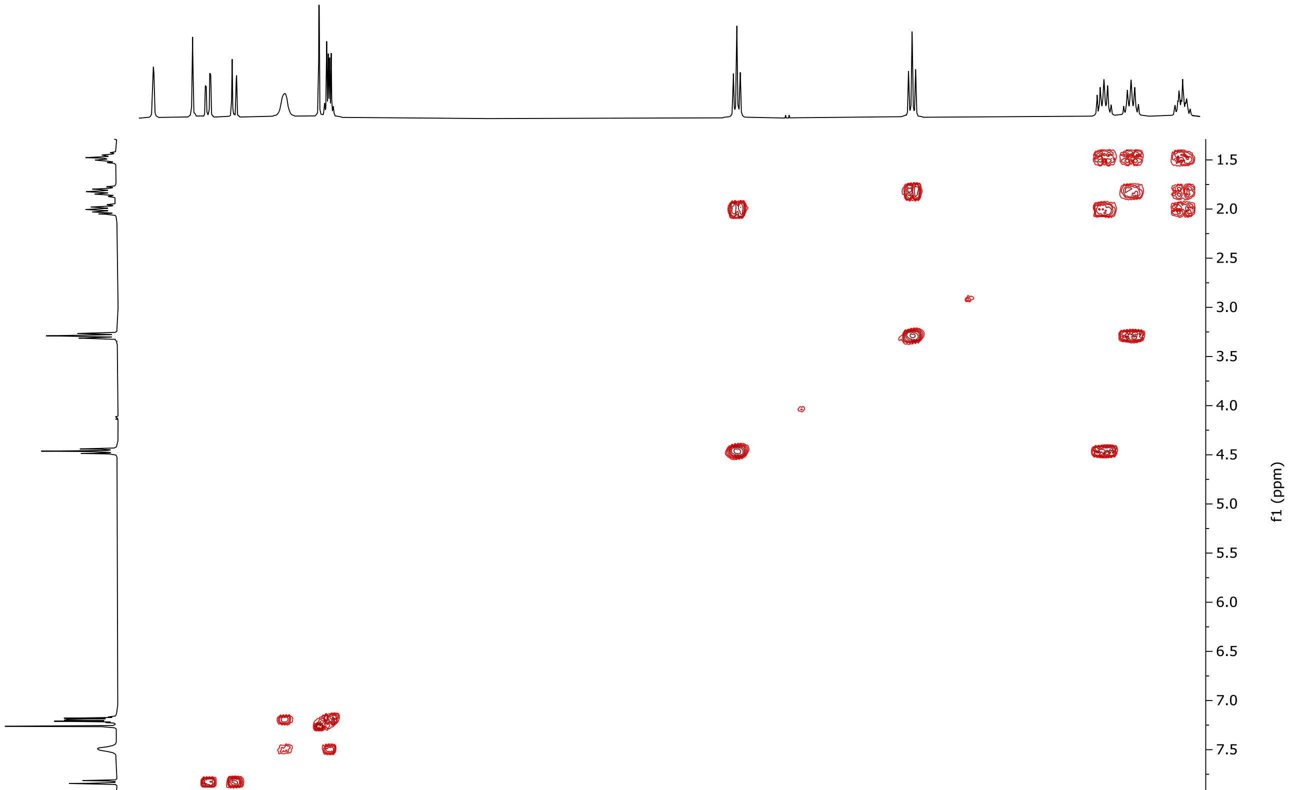

Figure S41. ^{1}H/^{1}H COSY NMR spectrum of compound **M5** in $CDCl_3$.

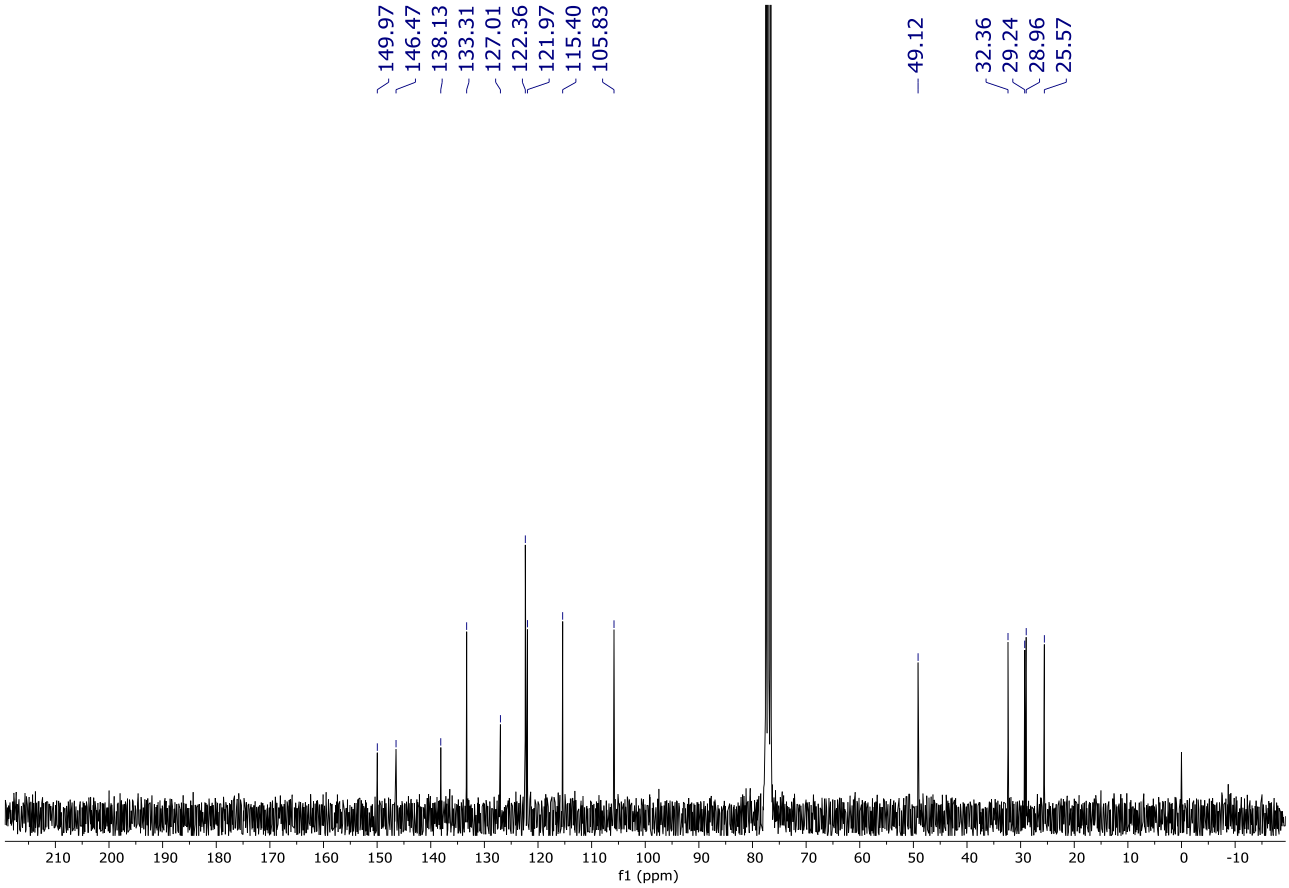

Figure S42. ^{13}C NMR spectrum of compound **M5** in $CDCl_3$.

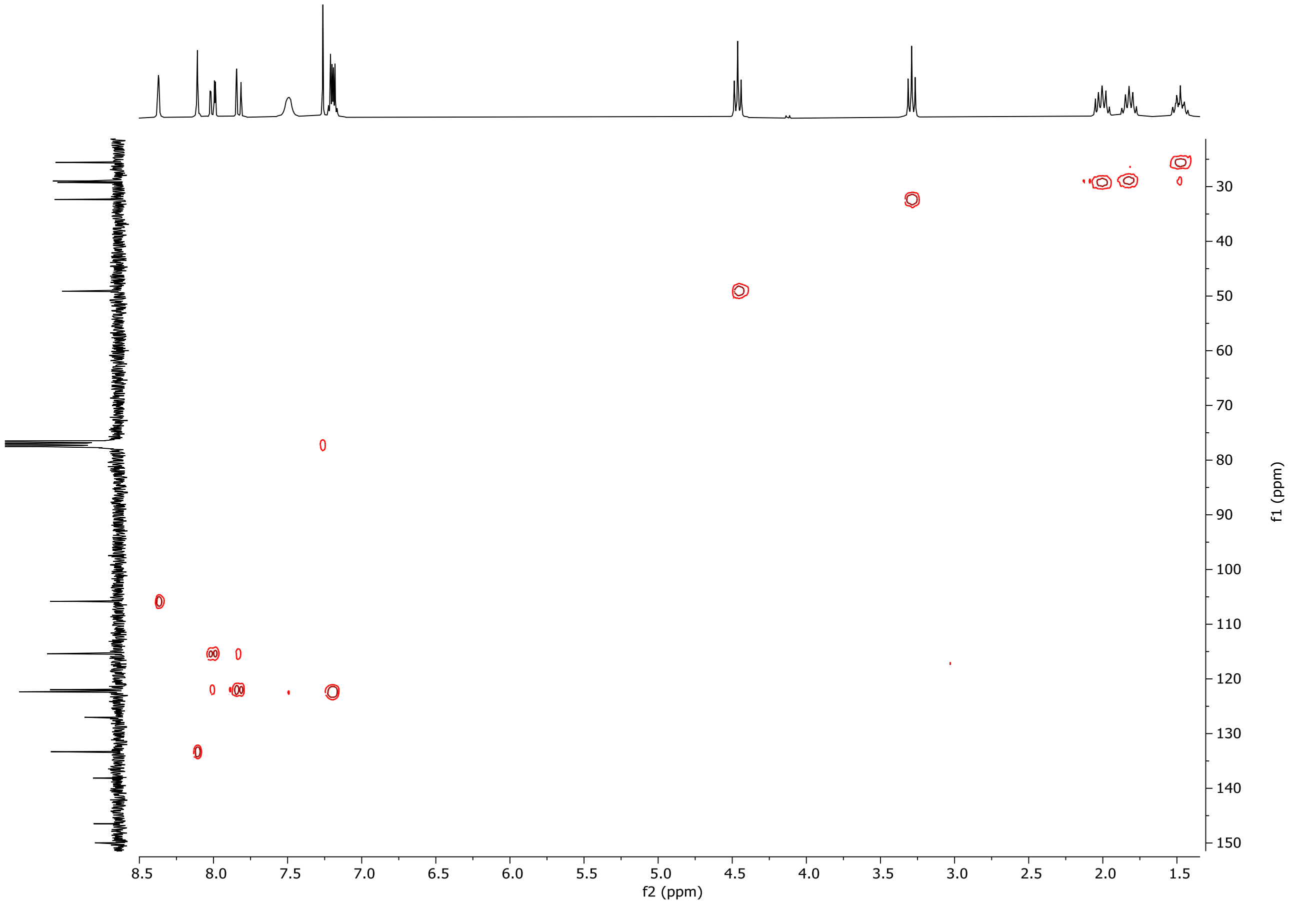

Figure S43. $^{1}H/^{13}C$ HSQC NMR spectrum of compound **M5** in $CDCl_3$.

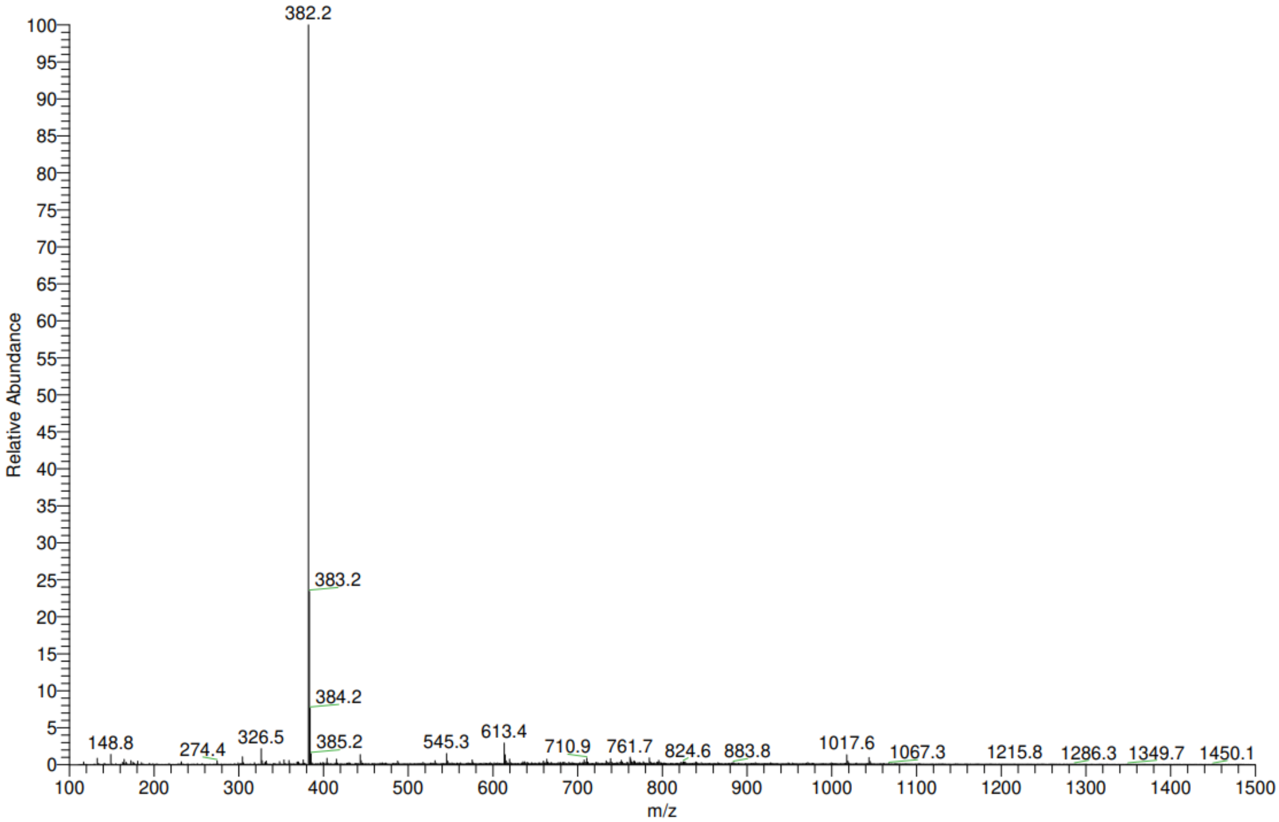

Figure S44. MS-ESI(+) spectrum of compound **M5**.

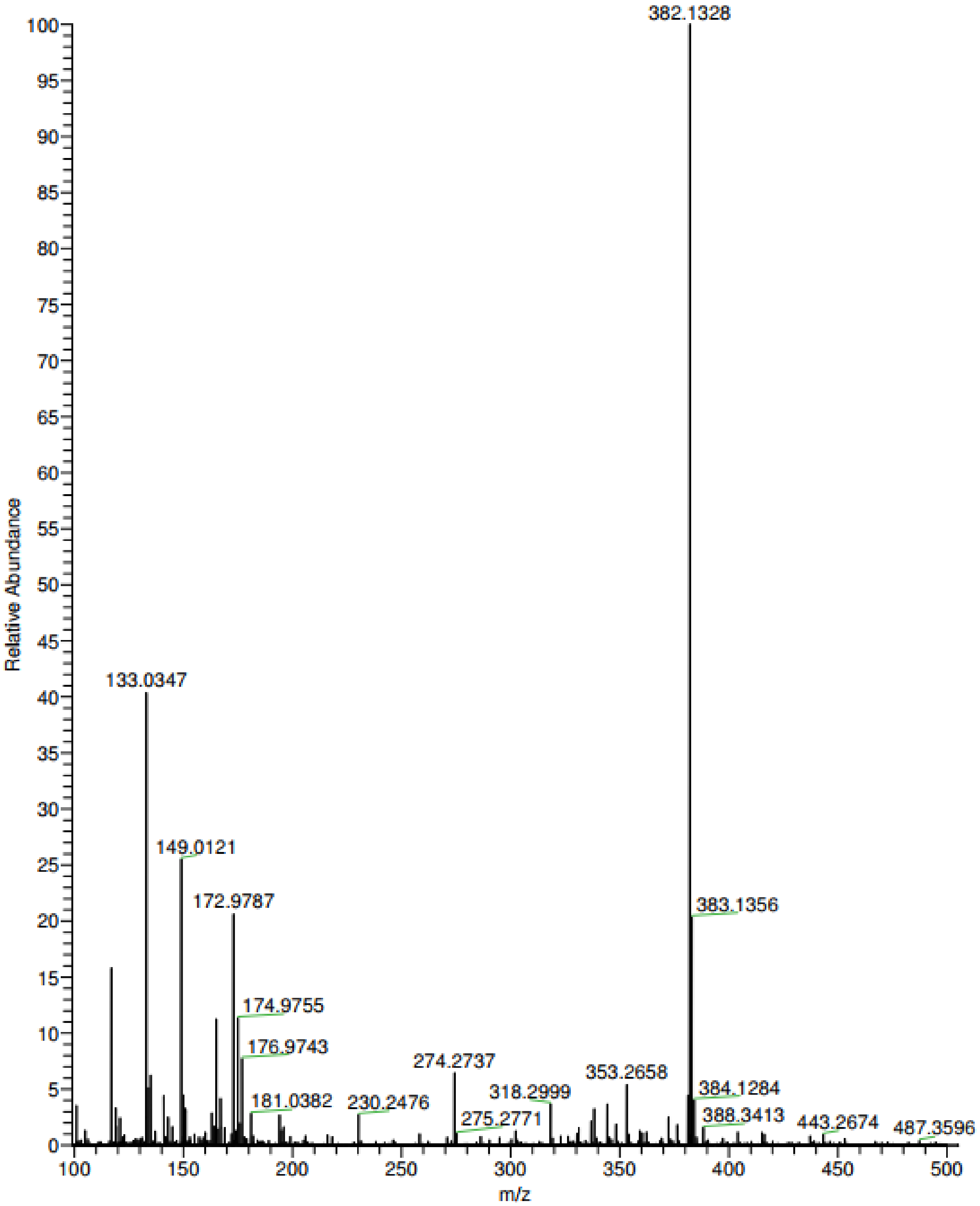

Figure S45. HRMS-ESI(+) spectrum of compound **M5**.

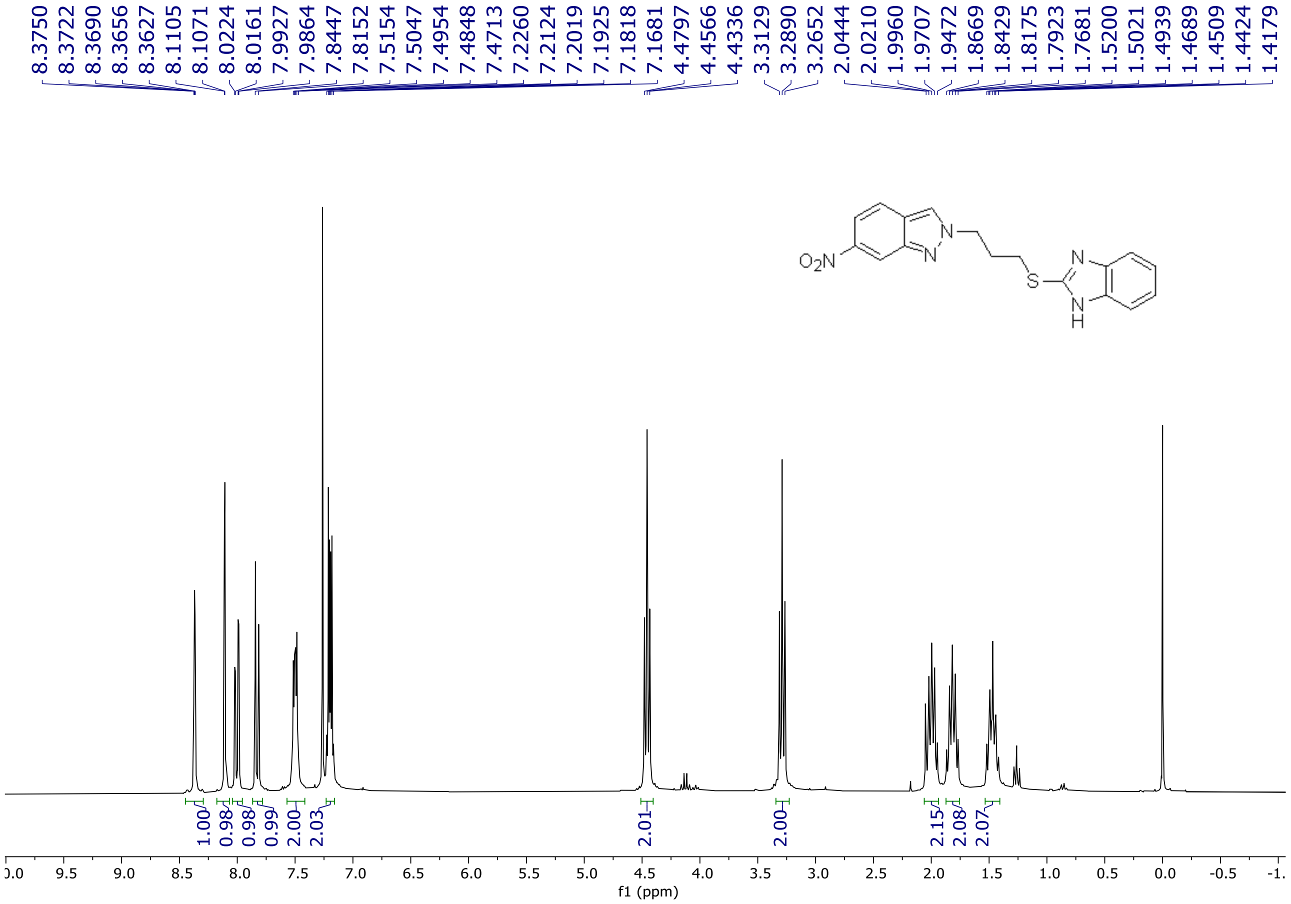

Figure S46. ^{1}H NMR spectrum of compound **M6** in $CDCl_3$.

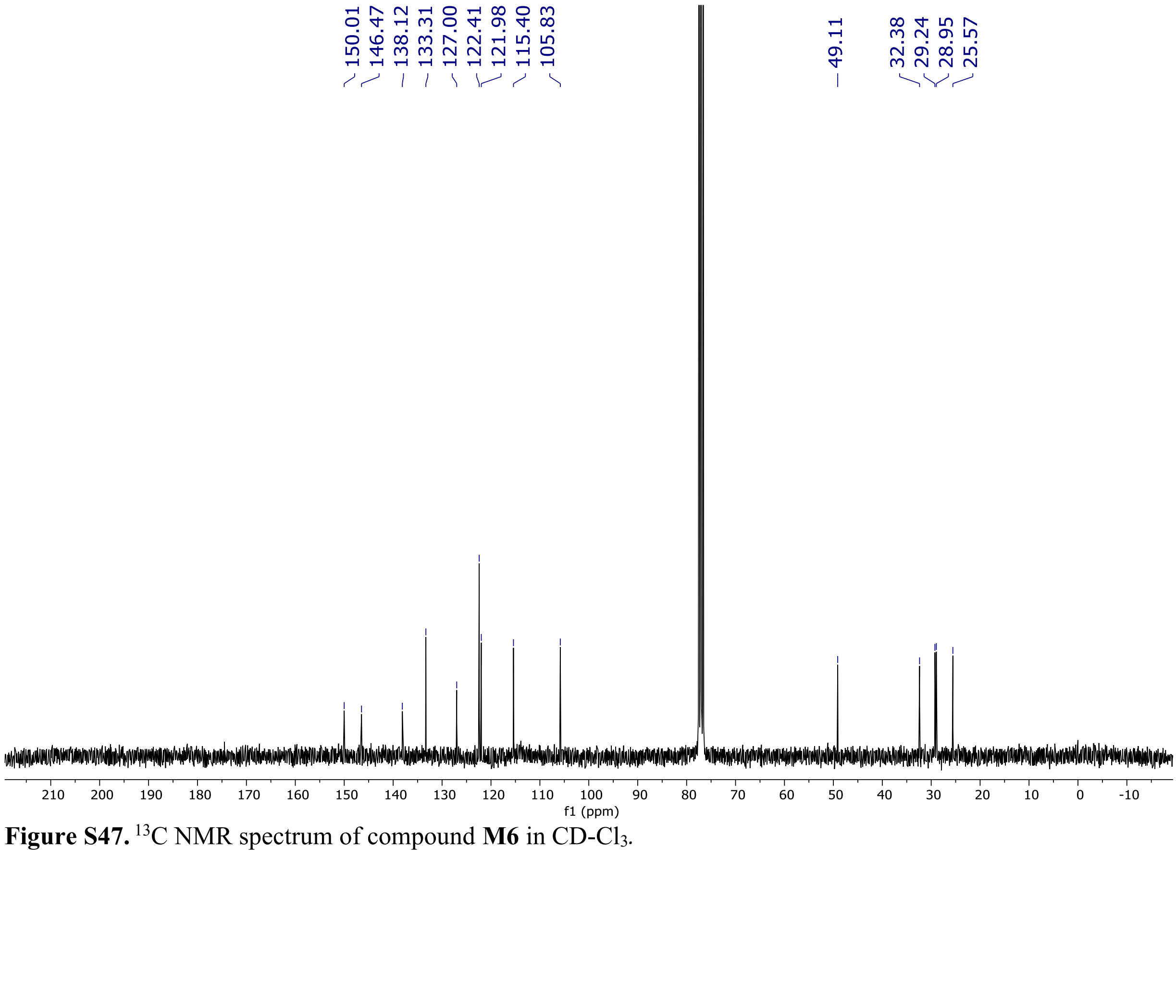

Figure S47. ^{13}C NMR spectrum of compound **M6** in CD-Cl_3.

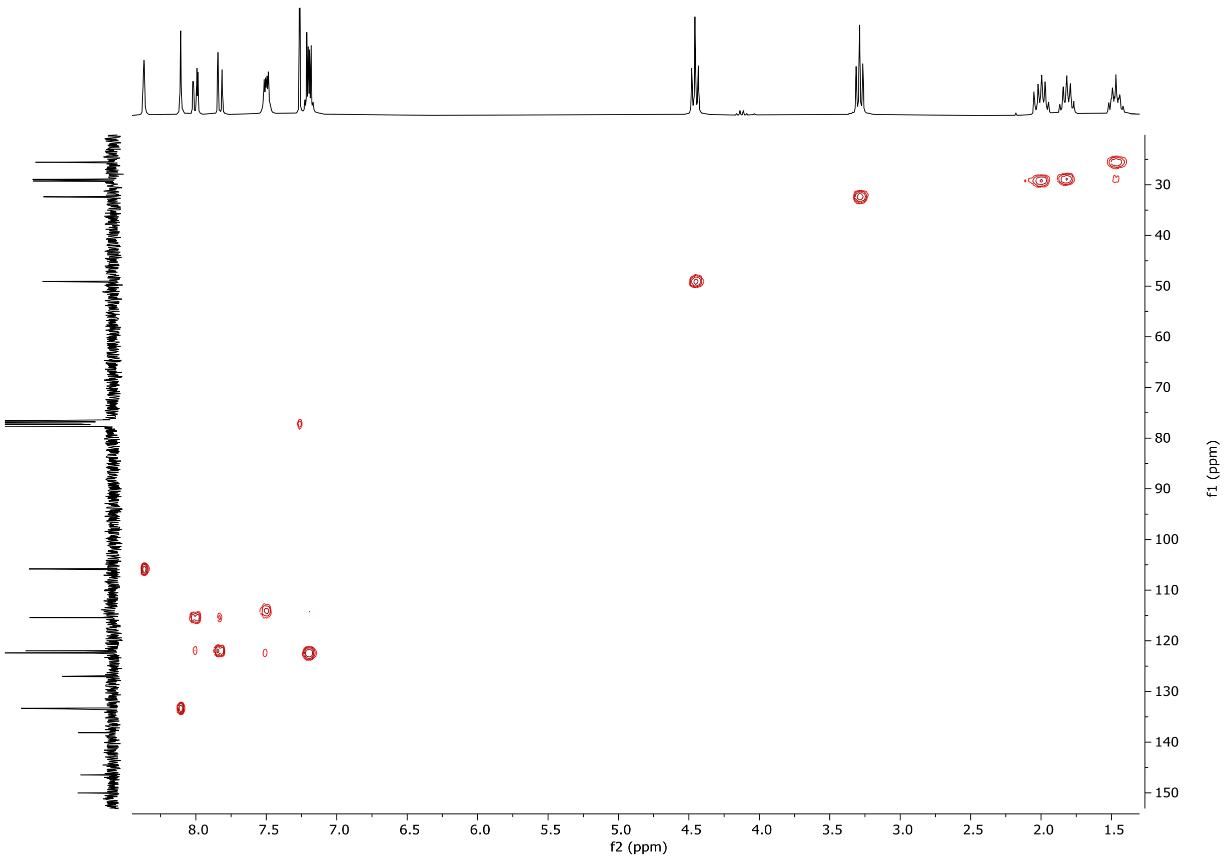

Figure S48. $^{1}H/^{13}C$ HSQC NMR spectrum of compound **M6** in $CDCl_3$.

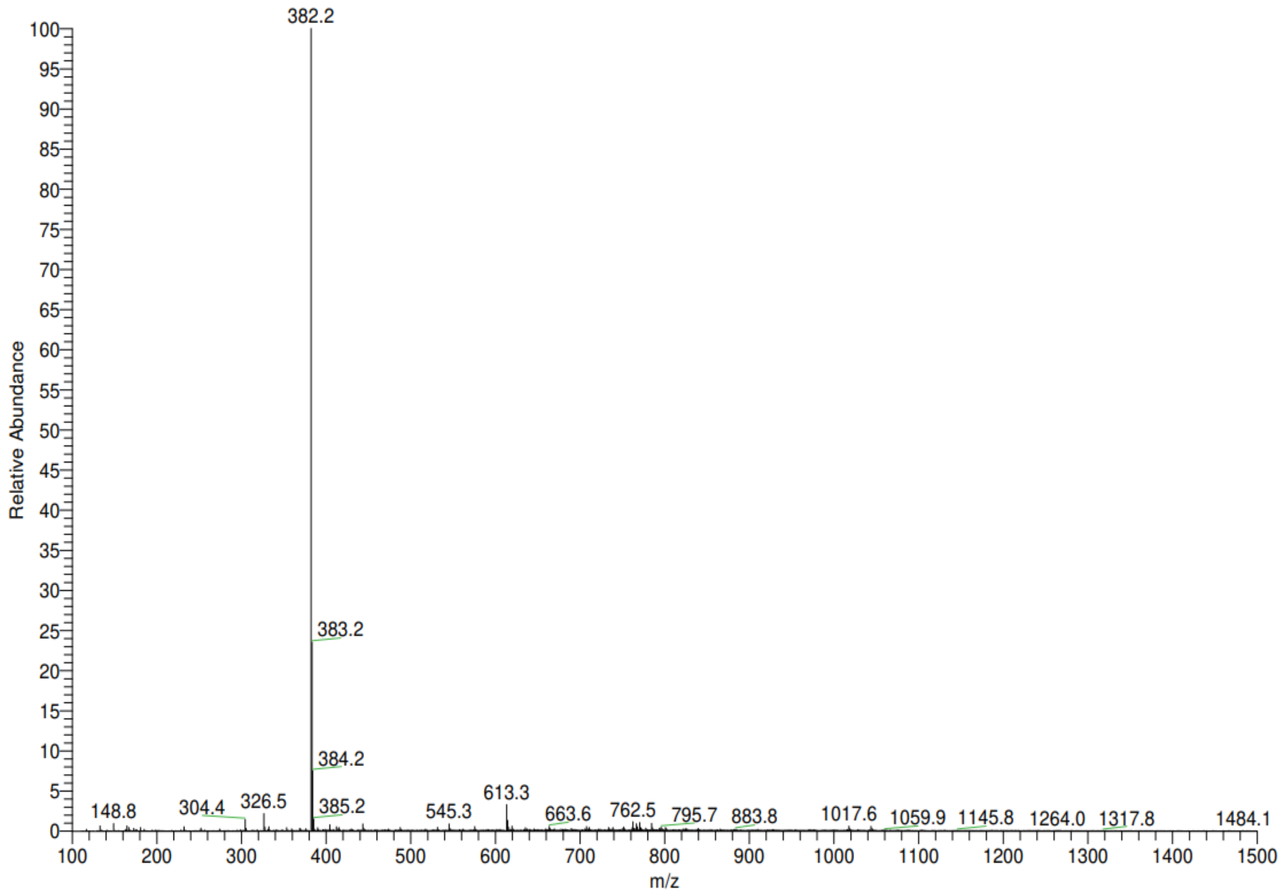

Figure S49. MS-ESI(+) spectrum of compound **M6**.

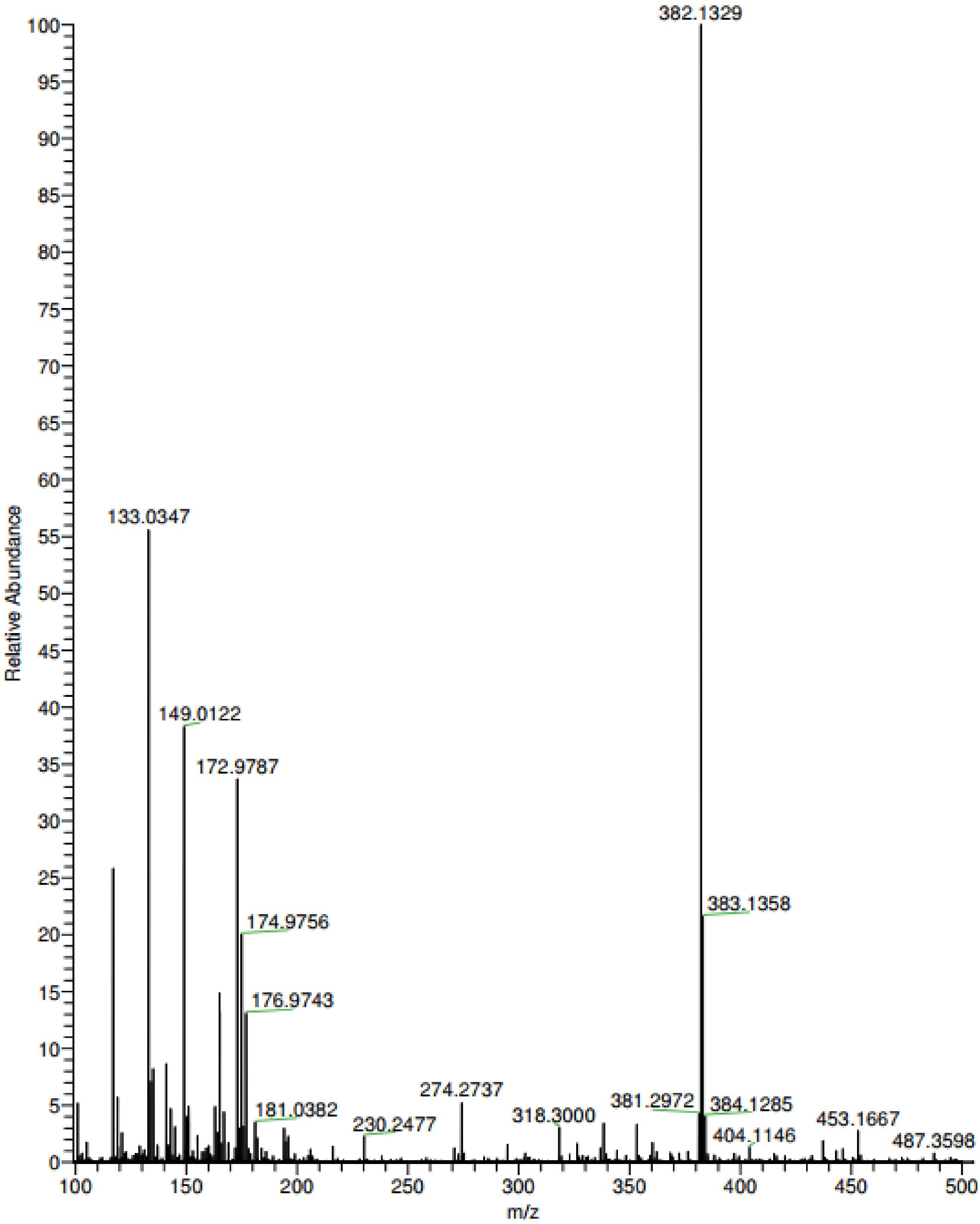

Figure S50. HRMS-ESI(+) spectrum of compound **M6**.

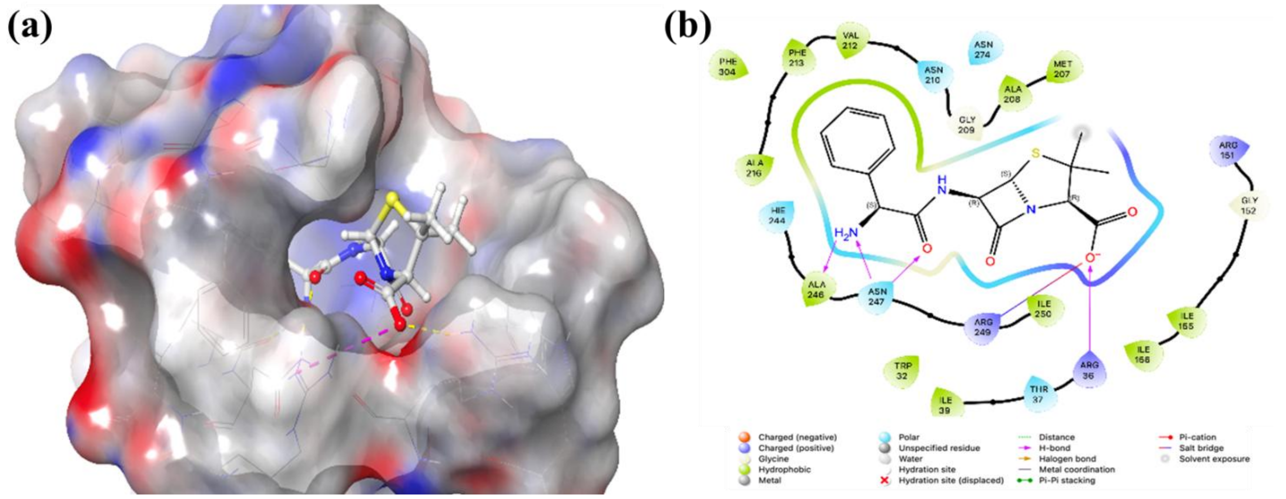

Figure S51. Representation of the MM-GB/SA refined IFD configuration of ampicillin within the active site cavity of 1HNJ. The electrostatic potential map of the protein's active site is depicted, where blue spheres denote regions of low electronic density, red spheres indicate regions of high electronic density, and grey spheres represent hydrophobic regions. The ligands' bound conformations are displayed using a ball and stick model, with grey denoting carbon, blue representing nitrogen, red for oxygen, and yellow for sulfur atoms. **(a)** Depiction of the electrostatic potential map of the protein's active site alongside the bound conformation of the ligand, illustrated in ball-and-stick format. **(b)** Diagram illustrating the interactions of the ampicillin-protein complex docking pose, highlighting significant hydrogen bonds, solvent-accessible regions, salt bridges, and hydrophobic interactions.

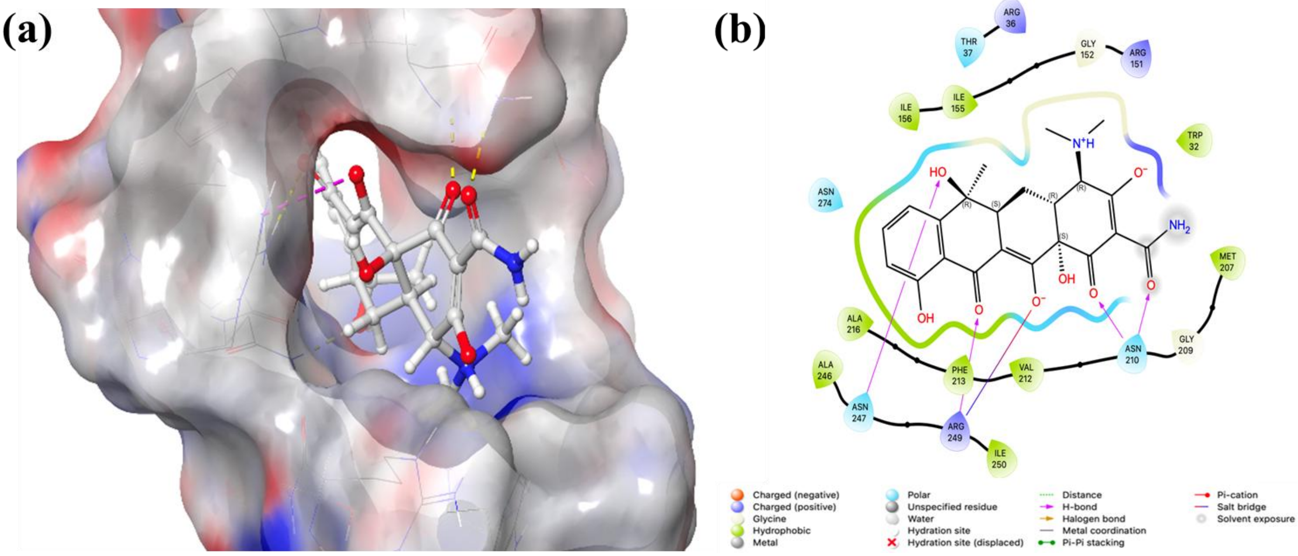

Figure S52. Representation of the IFD configuration refined by MM-GB/SA of tetracycline within the active site cavity of 1HNJ. The electrostatic potential map of the protein's active site is depicted, where blue spheres denote regions of low electronic density, red spheres indicate regions of high electronic density, and grey spheres represent hydrophobic regions. The ligands' bound conformations are displayed using a ball and stick model, with grey denoting carbon, blue representing nitrogen, red for oxygen, and yellow for sulfur atoms. **(a)** The electrostatic potential map of the protein's active site alongside the conformation of the bound ligand, depicted in ball-and-stick representation. **(b)** Ligand interaction diagram illustrating the docking pose of the tetracycline-protein complex, highlighting key hydrogen bonds, the solvent-exposed region, and hydrophobic interactions.

Table S1. Diameters of inhibition zones (mm) for compounds **M1-M6** against pathogenic Gram-positive and Gram-negative bacteria.

	**Bacterial Strains**	**M-1**	**M-2**	**M-3**	**M-4**	**M-5**	**M-6**	**TET**
Gram positive	*Staphylococcus aureus* ATCC 25923	30 mm (+++)	26 mm (+++)	34 mm (+++)	29 mm (+++)	21 mm (++)	35 mm (+++)	25 mm (+++)
	Enterococcus faecalis ATCC 29212	29 mm (+++)	18 mm (++)	16 mm (++)	34 mm (+++)	14 mm (+)	18 mm (++)	29 mm (+++)
	Bacillus cereus ATCC 14579	25 mm (++)	19 mm (++)	29 mm (+++)	23 mm (+++)	19 mm (++)	30 mm (+++)	23 mm (+++)
	Lactobacillus plantarum ATCC 14917	29 mm (+++)	19 mm (+++)	37 mm (+++)	26 mm (+++)	19 mm (+++)	13 mm (+)	28 mm (+++)
Gram negative	*Salmonella enteritidis* ATCC 25928	16 mm (++)	20 mm (+++)	15 mm (++)	24 mm (+++)	19 mm (++)	26 mm (+++)	28 mm (+++)
	Escherichia coli ATCC 25922	20 mm (+++)	20 mm (+++)	24 mm (+++)	29 mm (+++)	26 mm (+++)	27 mm (+++)	26 mm (+++)
	Campylobacter coli ATCC 43478	29 mm (+++)	16 mm (+++)	24 mm (+++)	22 mm (+++)	28 mm (+++)	15 mm (+++)	31 mm (+++)
	Campylobacter jejuni ATCC 33560	35 mm (+++)	18 mm (+++)	35 mm (+++)	31 mm (+++)	29 mm (+++)	19 mm (+++)	27 mm (+++)

Sensitivity Scale: −: Non-Sensitive (IZD < 8 mm); **+:** Weakly Sensitive (9 < IZD < 14 mm); **++:** Moderately Sensitive (15 < IZD < 19 mm); **+++:** Highly sensitive (IZD > 20 mm) **Ponce et al. (2008)**

Table S2. Diameters of inhibition zones (mm) for compounds **M1-M6** against pathogenic yeasts.

Yeast Strains	**RM-1**	**RM-2**	**RM-3**	**RM-4**	**RM-5**	**RM-6**	**AmB**
Saccharomyces cerevisiae ATCC 9763	27 mm (+++)	21 mm (+++)	23 mm (+++)	14 mm (+)	22 mm (++)	29 mm (+++)	26 mm (+++)
Candida albicans	25 mm (+++)	21 mm (+++)	28 mm (++)	14 mm (+)	18 mm (+++)	31 mm (+++)	23 mm (+++)
Candida tropicalis	20 mm (+++)	16 mm (++)	25 mm (+++)	13 mm (+)	21 mm (+++)	31 mm (+++)	21 mm (+++)
Candida glabrata	29 mm (+++)	21 mm (+++)	26 mm (+++)	12 mm (+)	27 mm (+++)	35 mm (+++)	21 mm (+++)

Sensitivity Scale: −: Non-Sensitive (IZD < 8 mm); **+:** Weakly Sensitive (9 < IZD < 14 mm); **++:** Moderately Sensitive (15 < IZD < 19 mm); **+++:** Highly sensitive (IZD > 20 mm) **Ponce et al. (2008)**
